# The Role of PANoptosis-Related Genes in Predicting Breast Cancer Survival and Immune Prospect

**DOI:** 10.1155/bmri/3423698

**Published:** 2025-05-28

**Authors:** Yuxi Zhang, Zheming Liu, Yixuan Zhang, Xue Zhang, Yi Yao, Chi Zhang

**Affiliations:** ^1^Department of Radiation Oncology, The First Affiliated Hospital With Nanjing Medical University, Nanjing, China; ^2^Cancer Center, Renmin Hospital of Wuhan University, Wuhan, China; ^3^Department of Breast, Renmin Hospital of Wuhan University, Wuhan, China

**Keywords:** breast cancer, machine learning, medicine efficiency, PANoptosis, prediction pattern, tumor immune microenvironment

## Abstract

**Background:** The function of PANoptosis in breast cancer (BC) remains indistinct. We constructed a nomogram model to predict the prognosis of BC to identify high-risk patients and help them receive more accurate treatment.

**Method:** We used Cox regression and least absolute shrinkage and selection operator (LASSO) algorithm to select PANoptosis-related genes (PRGs) and calculated the PANoptosis-related score (PRS) by LASSO coefficient. Through functional enrichment, somatic mutation, and tumor microenvironment (TME) analysis, we completed the identification of PANoptosis-related immune cells and difference analysis of drug sensitivity and then verified key genes by performing survival analysis.

**Results:** Patients were divided into low- and high-risk cohorts depending on PRS, and the negative association between risk scores and overall survival was disclosed. Analysis showed that differentially expressed genes in the two risk cohorts were mainly concentrated among pathways related to the immune system. Moreover, we detected distinguished differences in immune checkpoints, tumor mutation load, and TME in the two cohorts. Furthermore, KLHDC7B, GNG8, IGKV1OR2-108, and IGHD were identified as key genes. We also found that hub genes were highly expressed in tumor tissues, while B cells, CD4+, and CD8+ T cells pretended to be positive among the hub gene–negative cohort. Prognosis analysis showed that pivotal genes had adverse effects on survival over time.

**Conclusion:** We built a precise prediction model based on risk scores and proved the significance of PRGs in BC TME and medicine sensitivity regulation, providing key perception for subsequent molecular mechanism studies and contributing to more personalized treatment decisions in clinical practice.

## 1. Introduction

According to recent findings from the International Agency for Research on Cancer, breast cancer (BC) stands as the most prevalent malignant tumor among women globally and ranks second only to lung cancer in causing cancer-related fatalities, thereby constituting a significant threat to the physical and mental well-being of women worldwide [1, 2]. The therapeutic arsenal for BC encompasses a range of modalities including surgery, radiation therapy, chemotherapy, endocrine therapy, and targeted therapy [3, 4]. Continuous strides in medical research have heralded the advent of innovative treatment technologies and pharmaceuticals, expanding the therapeutic options and instilling hope in BC patients. Nevertheless, given the intricacy and heterogeneity of BC, it is imperative to identify high-risk individuals and tailor treatment strategies in accordance with their pathological attributes [5, 6].

PANoptosis represents a biological paradigm that extends beyond the conventional apoptotic framework, encapsulating a wider array of cellular demise mechanisms [7]. This concept not only includes traditional apoptosis but also encompasses other forms of cell death such as necrosis, autophagic cell death, and ferroptosis [8, 9]. These varied modes of cell death, while differing in their molecular mechanisms, morphological traits, and biological functions, collectively contribute to the cell's adaptive response to diverse internal and external environmental stresses [10]. Research has shown that PANoptosis influences tumorogenesis in colorectal cancer and modulates immunotherapy responses in gastric cancer [11–14]. Moreover, He et al. have identified a correlation between PANoptosis and the survival rates of BC patients [15]. However, to fully elucidate its function in progression and therapeutic responses of BC and to explore its potential as a prognostic indicator in conjunction with other clinical data, more extensive studies are required.

This study endeavors to fill this research gap by conducting an in-depth analysis of BC samples sourced from TCGA-BRCA and GEO datasets. Through the construction of a nomogram integrating PANoptosis-related genes (PRGs) and clinical features, complemented by a suite of machine learning techniques, we have pinpointed pivotal PRGs that influence BC outcomes. Leveraging these discoveries, we have crafted a novel prognostic model that stratifies BC patients into distinct risk categories, thereby offering a fresh instrument for survival prediction and treatment guidance. Our model has undergone rigorous validation across multiple datasets, and we have additionally delved into the molecular and immunological implications of PANoptosis risk in BC.

## 2. Materials and Methods

### 2.1. Data Acquisition

The clinical and genomic information pertaining to BC patients were meticulously acquired from TCGA (https://portal.gdc.cancer.gov) and GEO (https://www.ncbi.nlm.nih.gov/geo/, IDs: GSE20685, GSE20711, GSE42568, and GSE88770) repositories. A detailed catalog of 65 genes associated with PANoptosis, as identified in previous studies [14], was assembled. The datasets from TCGA and GEO were harmonized, with rigorous correction of batch effects implemented through the Combat algorithm within the R environment (V 4.3.1). Following integration, transcriptomic profiles from the two databases were meticulously synchronized with their respective survival outcomes, enabling the use of 1185 samples from TCGA BC cohort for model construction and internal validation. In parallel, 636 samples from the BC cohort in GEO were utilized for external test purposes. Individuals without follow-up statistics or those with incomplete clinical documentation were systematically omitted from the analysis. Ultimately, we involved a total of 1054 patients.

### 2.2. PRG Selection and Consistent Cluster Analysis

We utilized package “limma” to pinpoint differentially expressed genes (DEGs) linked to PANoptosis across diverse BC subtypes, filtering for genes that satisfied the conditions of adjusted *p* value < 0.05. Following this, we identified prognosis-related DEGs by the Kaplan–Meier analysis and Cox regression. Consensus clustering revealed distinct gene expression profiles by employing prognosis-related DEGs in BC. The most suitable clustering approach was chosen, and its prognostic relevance was assessed using Kaplan–Meier. We utilized principal component analysis (PCA) to evaluate the expression profiles. Clinical characteristics were compared via Wilcoxon test, and DEGs distinguishing between clusters were demonstrated based on the standard of *p* value < 0.05. Gene set variation analysis (GSVA) was used to evaluate differences in biological processes. We employed single-sample gene set enrichment analysis (ssGSEA) to evaluate immune infiltration cells and functional activities linked with the immune system. Gene Ontology (GO) was performed to investigate the biological function, and Kyoto Encyclopedia of Genes and Genomes (KEGG) analyses were utilized to investigate pathways related to DEGs related to PANoptosis (PRDEGs), with significance thresholds established at the value of *p* and *q* < 0.05.

### 2.3. Verification of PANoptosis-Related Premonitory Signature

To develop a prognostic character associated with PRGs, we first identified genes with significant differential expression between Clusters A and B. All in all, 1050 individuals were segregated into Cluster A and Cluster B depending on the expression of PRDEGs, with Cluster A comprising 548 patients and Cluster B consisting of 502 patients. The genes were then utilized to randomly allocate TCGA samples into a 1:1 ratio for the training set and the internal validation set. By integrating LASSO regression analysis with Cox regression analysis, our study constructed a prognostic signature consisting of four genes. This procedure was facilitated by R software packages of survival, survival minimum, and glmnet. Samples from GEO were assigned as an external validation set. PRS was calculated depending on the expression levels of these four genes, while individuals were categorized into low- and high-risk cohorts according to the median PRS. The survival discrepancy between the two risk groups was evaluated using the Kaplan–Meier analysis. The predictive performance of PRS was assessed through the receiver operating characteristic (ROC) curve and its corresponding area under the curve (AUC).

### 2.4. Construction of a Predictive PANoptosis-Related Nomogram

Our team has constructed a nomogram model combining PRS through various prognostic features with a comprehensive set of clinical–pathological and treatment factors. To improve clarity and accuracy, we have thoughtfully designed calibration plots that visually depict the agreement or discrepancies between the prognostic predictions of our model and survival outcomes in patients diagnosed with BC. These visual aids emphasize the reliability and accuracy of our predictive model, thereby enhancing its applicability in clinical contexts.

### 2.5. Immunogenomic Landscape Analysis

To clarify the relationship between tumor microenvironment (TME) and PRS, we employed CIBERSORT to assess the levels of immune infiltrating cells in various samples in low-risk and high-risk cohorts [16]. The scores of TME, which included ESTIMATE, immune, and stromal scores, were compared between the low-risk and high-risk cohorts through the Wilcoxon test. Furthermore, we calculated patients' tumor mutation burden (TMB) scores, and association between TMB and PRS was examined by Spearman's correlation method [17].

### 2.6. Estimation of Therapeutic Effect

Our team employed “oncoPredict” package in R software to ascertain the half maximal inhibitory concentration (IC_50_) values, leveraging the GDSC2 dataset sourced from the Genomics of Drug Sensitivity in Cancer (GDSC) database [18]. Moreover, we assessed different levels of expression of genes implicated in drug sensitivity by consulting the DrugBank database. This analysis revealed distinct patterns of drug sensitivity between the low-risk and high-risk groups [19].

## 3. Results

### 3.1. Identification of PRGs in BC Patients

**Figure 1:**
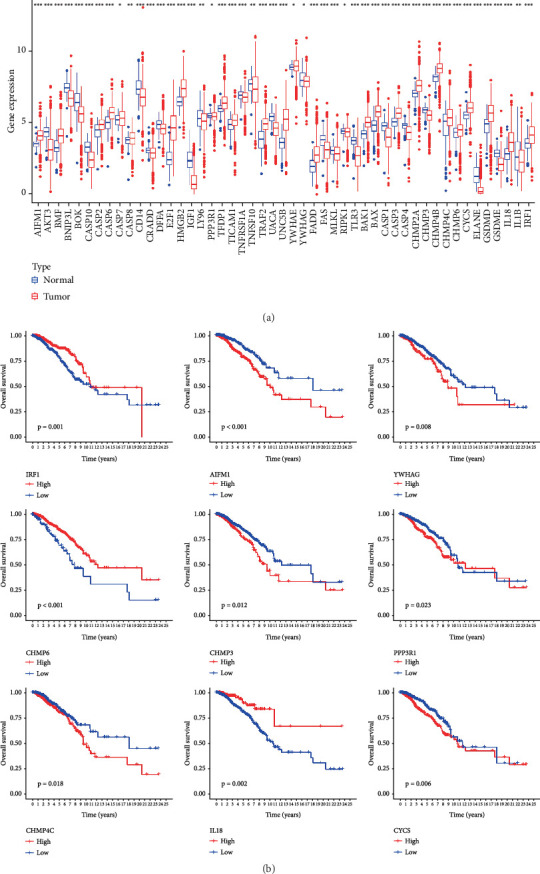
Identification of the PANoptosis-related signature. (a) The expression of DEGs in normal and tumor tissues; (b) Kaplan–Meier analysis of 9 DEGs with statistically different survival rates between the high- and low-expression groups.

Expression data derived from 1185 BC samples were acquired from TCGA dataset, encompassing 112 normal and 1073 tumor samples for comparative analysis. Sixty-five PRGs were verified based on prior reports, as presented in Supporting Information 1. Among them, 49 DEGs demonstrated significant different expression in BC samples compared with normal tissue, as illustrated in Figure 1a. Notably, a total of 49 DEGs were upregulated in tumor samples (*p* < 0.05). To assess the prognostic impact of the genes, survival curves were utilized to visualize the correlation between DEGs and BC patients' prognosis. Nine DEGs revealed statistically distinct survival rates between low- and high-expression cohorts, as depicted in Figure 1b.

### 3.2. Verification of Prognostic PRG Clusters in BC

**Figure 2:**
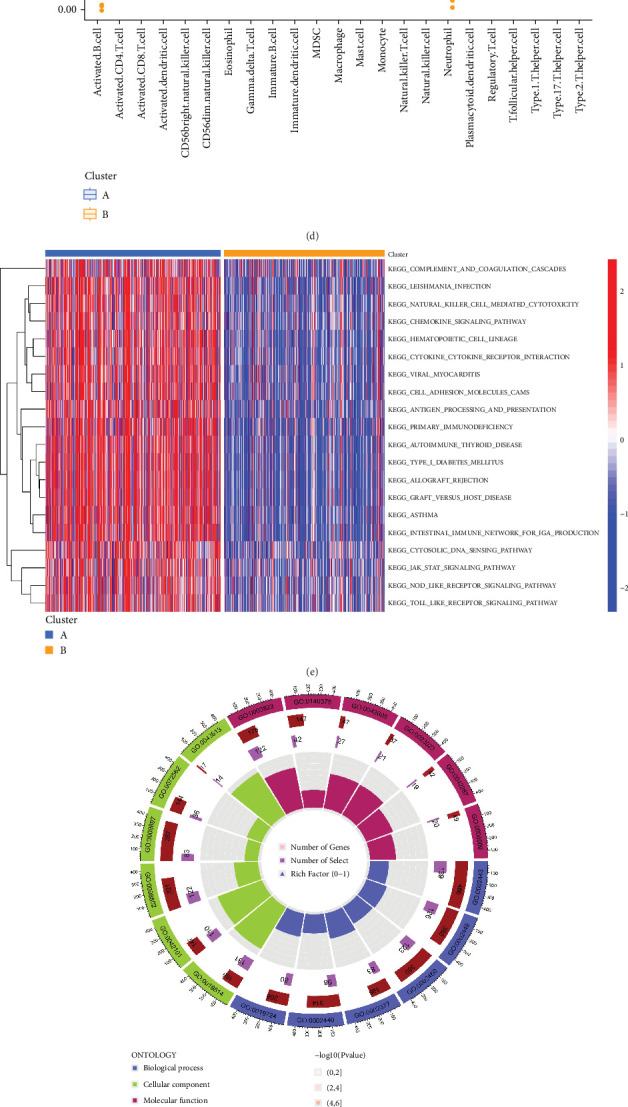
Verification of PANoptosis-related gene clusters. (a) The optimal cluster is two (*k* = 2). (b) Kaplan–Meier analysis of patients in Clusters A and B (*p* = 0.001). (c) PCA between Clusters A and B. (d) ssGSEA of immune infiltration between Clusters A and B. (e) GSVA of immune-related pathways between Clusters A and B. (f) Circle plot of GO enrichment analysis. (g) Bubble plot of GO enrichment analysis. (h) Bubble plot of KEGG enrichment analysis.

PRDEGs were identified through the Kaplan–Meier analysis and univariate Cox regression, identifying nine genes significantly associated with overall survival (OS): AIFM1 (HR = 1.77, 95%CI = 1.28–2.46, *p* = 0.0006), PPP3R1 (1.51, 1.01–2.26, 0.0446), YWHAG (1.39, 1.04–1.85, 0.0238), CHMP3 (1.55, 1.11–2.17, 0.0096), CHMP4C (1.21, 1.01–1.44, 0.0401), CHMP6 (0.79, 0.62–0.99, 0.0471), CYCS (1.31, 1.03–1.68, 0.0273), IL-18 (0.80, 0.69–0.95, 0.0083), and IRF1 (0.77, 0.64–0.93, 0.0058). Our investigation explored the correlation between prognostic PRDEGs' consensus clustering and BC patients' survival outcomes and characteristics. Through incrementally adjusting the variable of clustering (*k*), we established that a *k* value of 2 provided a satisfactory classification (Figure 2a and Supporting Information 2). Patients in Cluster A exhibited notably prolonged survival compared to those in Cluster B (*p* = 0.001), as shown in Figure 2b. It showed clear difference between Clusters A and B by PCA (Figure 2c). To assess variations of immune cell infiltration, ssGSEA was conducted, revealing that Cluster A exhibited higher levels of diverse immune cell types than Cluster B (Figure 2d). GSVA assessment demonstrated that Cluster A was notably enriched in pathways related to immunity, including natural killer cell–mediated antigen processing, presentation and cytotoxicity, and signaling pathways associated with primary immunodeficiency (Figure 2e). Utilizing these findings, KEGG and GO analyses were implemented to outline the pertinent biological processes, cellular components, molecular functions, and pathways (Figures 2f, 2g, and 2h).

### 3.3. Validation of Prognostic Signatures for PRGs

**Figure 3:**
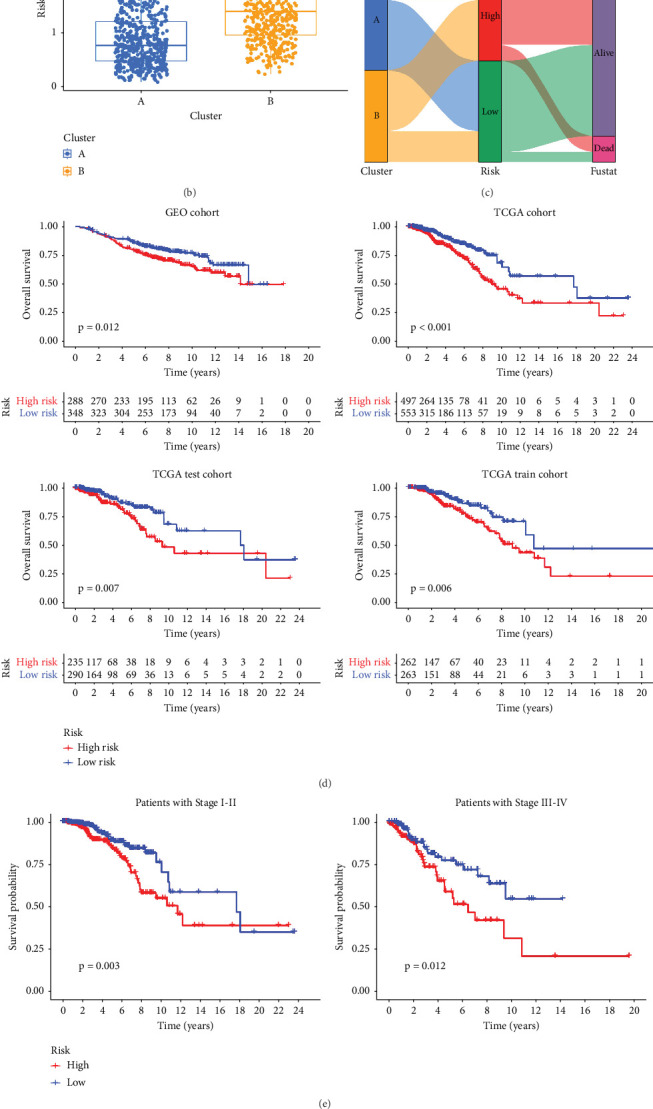
Validation of prognostic signatures for PRGs. (a) Coefficient profiles of PRGs in the LASSO (left) and identification of the best parameter in LASSO (right). (b) Boxplot of PRS between Cluster A and Cluster B. (c) Sankey plot of the correlation among risk groups, clusters, and survival outcomes. (d) Kaplan–Meier analysis of the low-risk group and the high-risk group in TCGA and GEO databases (all *p* < 0.05). (e) Kaplan–Meier analysis of the low-risk group and the high-risk group in stage subgroups (both *p* < 0.05). (f) Time-dependent ROC curves of the nomogram in predicting the 1-, 3-, and 5-year OS.

We conducted an intersection analysis of DEGs across two clusters, employing criteria of *p* < 0.05, which resulted in the identification of 826 genes. Following this, LASSO regression analyses were applied to refine this set of genes (Figure 3a). This approach successfully identified the most potent predictive markers and developed a prognostic index capable of forecasting clinical outcomes. The dashed vertical line in the plot indicates the optimal value of log*λ*, which corresponds to the minimum deviance in the partial likelihood. The PRS was computed using the coefficients derived from each intersected gene via the LASSO algorithm. Boxplot revealed elevated PRS in Cluster B relative to Cluster A (Figure 3b). A Sankey diagram was utilized to visualize the relationships among risk groups, clusters, and survival outcomes (Figure 3c). Patients classified in the high-risk group indicated a notably diminished survival probability compared to those in the low-risk group across both TCGA and GEO databases (*p* < 0.05), as depicted in Figure 3d. Subgroup analysis confirmed the efficacy of the risk model in discerning survival prognoses within Stage I–II and Stage III–IV cohorts (Figure 3e), implying that this risk categorization may be particularly indicative of the prognosis for locally advanced BC. To assess the predictive accuracy of PRS, ROC curves were constructed using data from TCGA and GEO databases, showing AUC values for 1-, 3-, and 5-year survival at 0.699, 0.651, and 0.601, respectively, which surpassed the predictive performance of other clinical variables (Figure 3f).

### 3.4. Construction and Verification of BC Prognostic Nomogram

**Figure 4:**
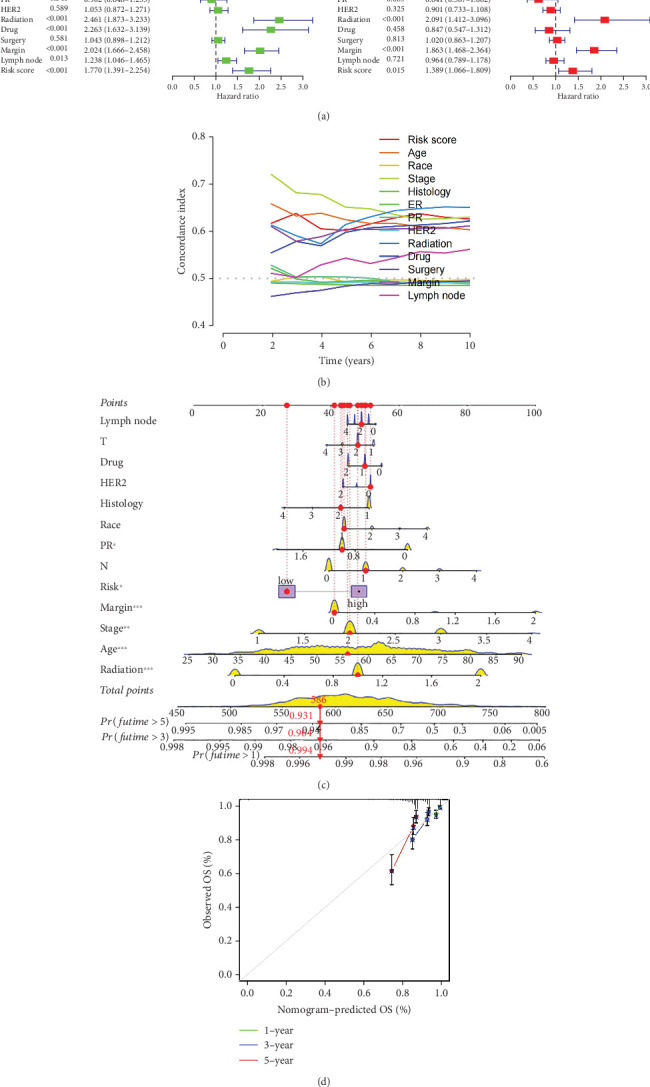
Construction of a clinical nomogram. (a) Univariate (right) and multivariate (left) Cox regression analyses of the independent prognostic value of the model. (b) Concordance index curves of survival time. (c) Nomogram based on the PRS, age, stage, PR status, surgery margin, and radiation. (d) Calibration curves of the nomogram in predicting the 1-, 3-, and 5-year OS.

Our study conducted Cox regression to evaluate the independent prognostic value of the model in conjunction with various clinical data. The univariate analysis revealed significant associations of age (*p* < 0.001), tumor stage (*p* < 0.001), radiation therapy (*p* < 0.001), chemotherapy medication (*p* < 0.001), surgical margin status (*p* < 0.001), lymph node involvement (*p* = 0.013), and the derived PRS (*p* < 0.001) with OS (Figure 4a). Further, it is showed through multivariate Cox regression analysis that tumor stage (*p* < 0.001), radiation therapy (*p* < 0.001), surgical margin status (*p* < 0.001), and the risk score (*p* = 0.015) continued to show strong correlations with OS (Figure 4a). This analysis underscored the persistent relevance of clinical staging and PRS in predicting OS. The concordance index evaluation suggested that the PRS outperforms other clinical parameters in predicting survival (Figure 4b). To enhance predictive accuracy, PRS was combined with additional clinical characteristics to build a nomogram model (Figure 4c). Calibration plots confirmed a high degree of concordance among nomogram predicted outcomes and the actual survival rates of BC patients (Figure 4d), thereby validating the nomogram's precise prognostic capabilities for BC survival.

### 3.5. Comprehensive Analysis of Tumor Immune Microenvironment and Drug Sensitivity

**Figure 5:**
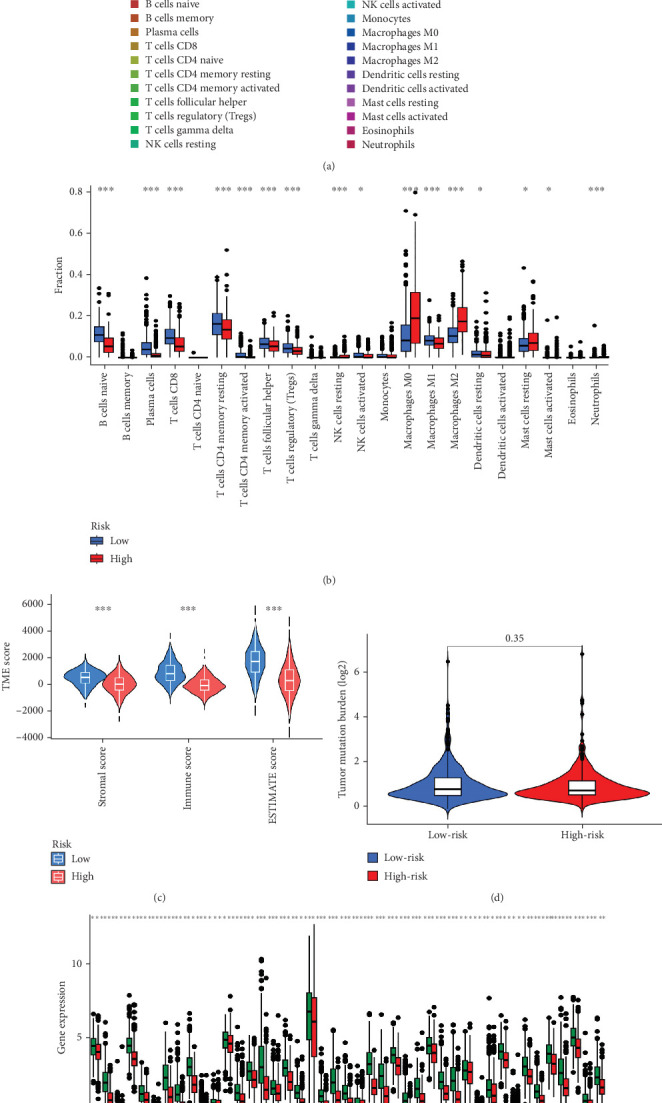
Immune landscape. (a, b) Difference of immune cell infiltration between the low- and high-PRS groups. (c) TME score discrepancy of the low- and high-PRS groups. (d) TMB in the low- and high-PRS groups. (e) Immune checkpoint gene expression in the low- and high-PRS groups.

**Figure 6:**
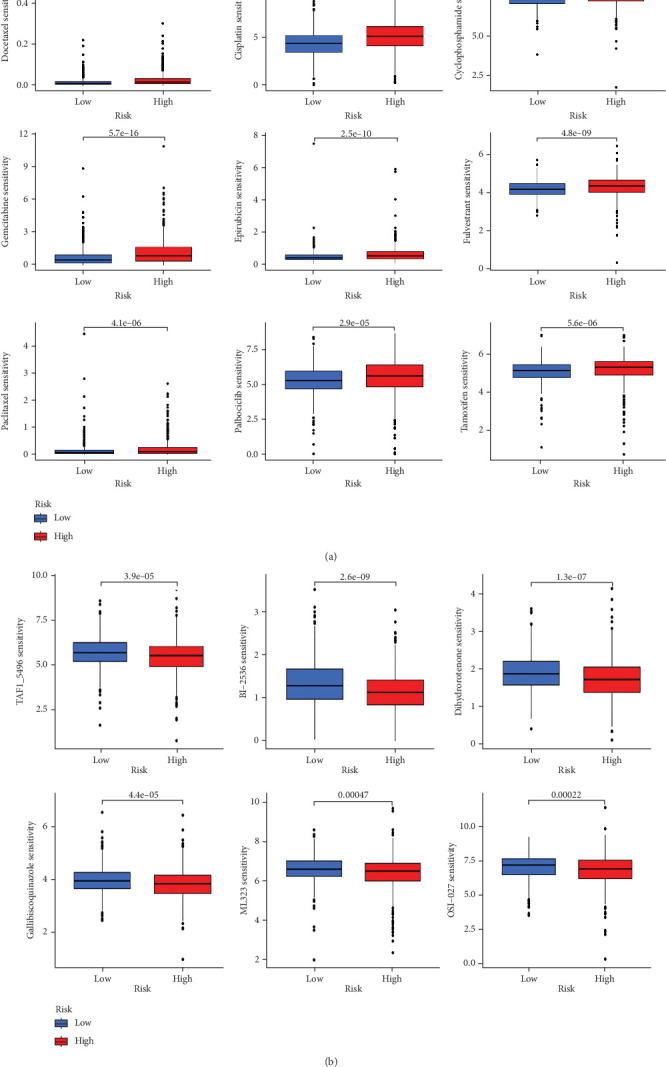
Drug sensitivity. (a, b) IC_50_ of antitumor drugs in the low- and high-PRS groups.

When comparing the infiltration of immune cells between the low-risk and high-risk groups, our data revealed a higher prevalence of M0 macrophages, M2 macrophages, and resting mast cells within high-risk cohort (Figure 5a,b). The analysis of TME scores indicated that the low-risk group had a higher stromal score but a lower immune score (Figure 5c). However, there was no significant correlation observed between TMB and the PRS (Figure 5d). In the context of predicting responses to checkpoint blockade therapy, the box plot showed differences in the expression of immune checkpoint genes across the two risk cohorts. All genes were more highly expressed in the low-risk cohort (Figure 5e). Additionally, IC_50_ values suggested that the high-risk group showed greater sensitivity to a range of chemotherapeutic agents, including cisplatin, cyclophosphamide, docetaxel, epirubicin, fulvestrant, gemcitabine, paclitaxel, palbociclib, and tamoxifen. In contrast, the low-risk cohort showed increased sensitivity to drugs such as BI-2536, dihydrorotenone, gallibiscoquinazole, ML323, OSI-027, and TAF1_5496 (Figure 6a,b and Supporting Information 3).

## 4. Discussion

Various programmed cell death (PCD) pathways, such as apoptosis, necroptosis, and pyroptosis, have been recognized as key factors influencing cancer progression [20]. The concept of PANoptosis integrates these distinct PCD pathways, allowing them to complement each other and act cooperatively across diverse stimuli [11]. This integration is orchestrated by the PANoptosome complex, a molecular structure essential for the execution of pyroptosis, apoptosis, and necroptosis mechanisms [7]. In the field of oncology, PANoptosis has been related to the control of colorectal cancer development and the efficacy of immune therapies in gastric cancer [13, 14]. Presently, certain researchers have focused on PANoptosis across diverse cancer forms such as hepatocellular carcinoma, gastric cancer, and pancreatic cancer [21–23]. Furthermore, genes associated with PANoptosis have demonstrated prognostic value for BC patients and influenced the immune TME [15]. Additionally, a previous study has examined the molecules that participate in the pathway of PANoptosis and proposed that targeting the PD-1/PD-L1 interaction could potentially engage the PANoptosis pathway, although the exact mechanisms in BC are still under investigation [24].

Notwithstanding the commonality of research subjects [15], this investigation utilized a synergistic approach involving Cox regression and LASSO algorithms to identify PANoptosis-associated genes. For the first time, it developed a highly precise nomogram that amalgamates PANoptosis gene signatures with clinical pathological data. Previous studies have revealed that Kelch domain–containing 7B (KLHDC7B) is overexpressed in BC and is distinguished related to gene modulation activity during BC tumorigenesis [25]. Mechanistically, KLHDC7B-DT directly binds to the IL-6 promoter, inducing chromatin structure opening in the IL-6 promoter region, activating IL-6 transcription, and upregulating IL-6 expression and secretion [26]. Yu et al. discovered that the four core genes CHIT1, GTSF1L, PLA2G2D, and GNG8 constitute the final features, suggesting that immune scoring depending on these four immune-related genes can serve as an auxiliary standard to effectively predict survival outcomes, tumor infiltration, and immunotherapy efficacy of cervical cancer [27]. One study constructed a prognosis signature related to TME, including risk factor IGKV1OR2-108, as an independent prognostic factor for BRCA. IGKV1OR2-108 synergistically promotes an immunosuppressive microenvironment in the high-risk scoring group, characterized by impaired migration of immune-suppressive neutrophils, natural killer cell cytotoxicity, and cytotoxic T lymphocytes [28]. Hsu et al. identified a subnetwork including six novel genes, including IGHD related to B cell–specific immunoglobulin, which proved that these genes are correlated with relapse-free survival and distal metastasis–free survival in triple-negative and general BC [29]. Various prognostic models have been established for BC patients. Nonetheless, none of these models surpassed the efficacy of our PANoptosis-centric nomogram, as evidenced by the AUC metric, highlighting the substantial significance of our study [30–32].

TME comprises a multifaceted structure encompassing stroma, cancer cells, endothelial cells, and immune cells [33]. The intricate interplay in the TME, involving both adaptive and innate immune cells, cell surface molecules, and extracellular immune factors, plays a pivotal role in tumourigenesis [34–36]. We developed a prognostic model utilizing PRS and performed extensive analyses comparing low-risk and high-risk cohorts. Our functional enrichment analysis proved that DEGs were enriched in various pathways related to the immune system. These observations imply that the divergent survival outcomes between low-risk and high-risk cohorts can be partially explained by distinct tumor immune microenvironments. To substantiate this hypothesis, we conducted analyses on TMB and immune cell infiltration.

TMB serves as a representative fatidic biomarker of cancer immunotherapy. Through our investigation, we identified elevated TMB scores within the high-risk cohort, suggesting that individuals with high PRS might derive greater benefit from immunotherapy than those with low-risk scores. Additionally, our team examined the relationship between scores and immune cell infiltration, uncovering a positive association with resting mast cells, M2 macrophages, and M0 macrophages, whereas a negative association was found with the majority of other immune cell types. The research underscores the role of tumor-promoting immune cells (including myeloid-derived suppressor cells, neutrophils, dendritic cells, macrophages, innate lymphoid cells, and cytokines) in modulating immune function, inhibiting T-cell antitumor responses, facilitating angiogenesis, and fostering cancer cell proliferation, invasion, and metastasis [37–39]. Conversely, NK cells, M1 cells, N1 cells, dendritic cell 1, T helper cell 1, and CD8+ T cells are characterized as tumor-suppressive infiltrating immune cells [40, 41]. Moreover, individuals in the high-risk group exhibited increased sensitivity to commonly administered chemotherapeutic agents, such as cisplatin, cyclophosphamide, docetaxel, epirubicin, fulvestrant, gemcitabine, paclitaxel, palbociclib, and tamoxifen.

Despite the strengths of our study, certain limitations are imperative to recognize. Initially, the model was developed and validated through retrospective analysis, thereby calling for prospective investigations in actual clinical scenarios to substantiate its practical applicability. Furthermore, our approach did not include immunohistochemistry analysis using tissue arrays, and additional mechanistic studies are essential to uncover the internal mechanisms of the PANoptosis-associated genes.

To conclude, this research has successfully developed and tested a prognostic model associated with PANoptosis, demonstrating substantial significance in forecasting survival rates among BC patients. Moreover, we executed extensive analyses on patients with both low and high PRS, thereby affirming the critical role of PANoptosis-linked gene signatures in influencing TME and medicine responsiveness in BC. These findings offer essential perspectives for future mechanistic investigations and support healthcare providers in formulating more tailored treatment strategies.

## Data Availability

The data used to support the findings of this study are included within the article and the supporting information files.

## Supporting Information

Supporting Information 1 Sixty-five PRGs verified based on prior reports.

Supporting Information 2 The incrementally adjusting variable of clustering (*k*).

Supporting Information 3 Distinct patterns of drug sensitivity between low-risk and high-risk groups.
