# Supplementary figures and images for: The Role of PANoptosis-Related Genes in Predicting Breast Cancer Survival and Immune Prospect (part 1 of 2)
Source: Biomed Res Int. 2025 May 28;2025:3423698. doi: 10.1155/bmri/3423698 (PMC12136870; doi:10.1155/bmri/3423698)

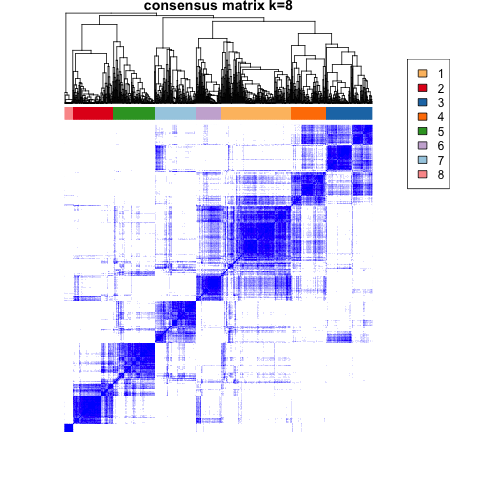

Supplement: Supporting Information 2 — The incrementally adjusting variable of clustering (k). [file 3423698.f2.zip › Supplementary Material 2/consensus008.png]

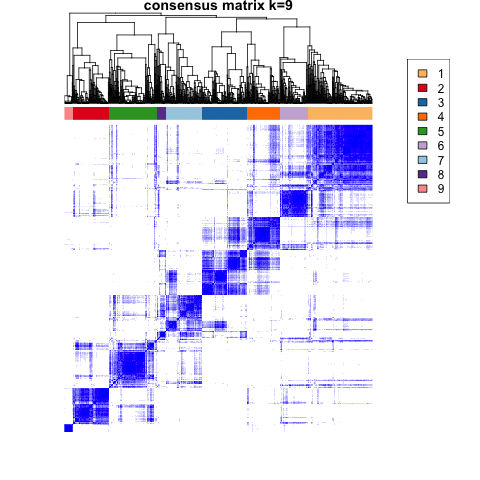

Supplement: Supporting Information 2 — The incrementally adjusting variable of clustering (k). [file 3423698.f2.zip › Supplementary Material 2/consensus009.png]

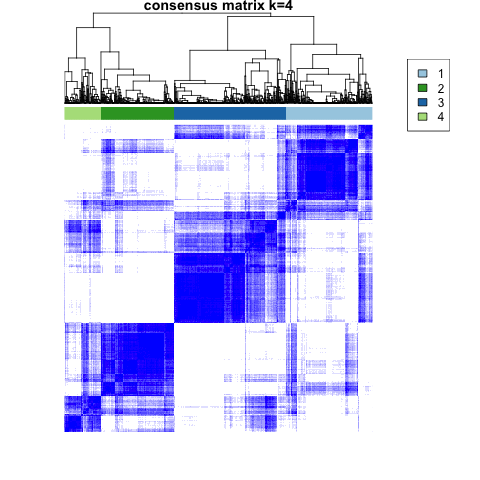

Supplement: Supporting Information 2 — The incrementally adjusting variable of clustering (k). [file 3423698.f2.zip › Supplementary Material 2/consensus004.png]

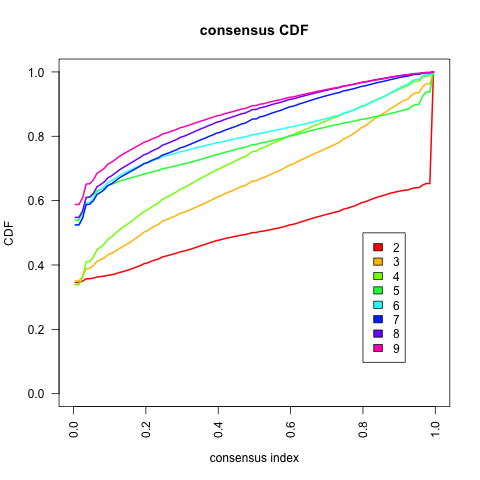

Supplement: Supporting Information 2 — The incrementally adjusting variable of clustering (k). [file 3423698.f2.zip › Supplementary Material 2/consensus010.png]

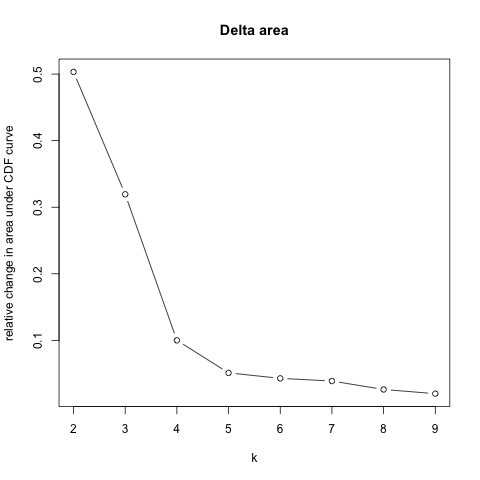

Supplement: Supporting Information 2 — The incrementally adjusting variable of clustering (k). [file 3423698.f2.zip › Supplementary Material 2/consensus011.png]

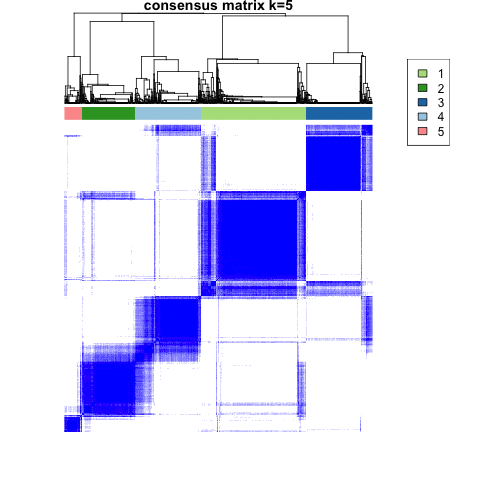

Supplement: Supporting Information 2 — The incrementally adjusting variable of clustering (k). [file 3423698.f2.zip › Supplementary Material 2/consensus005.png]

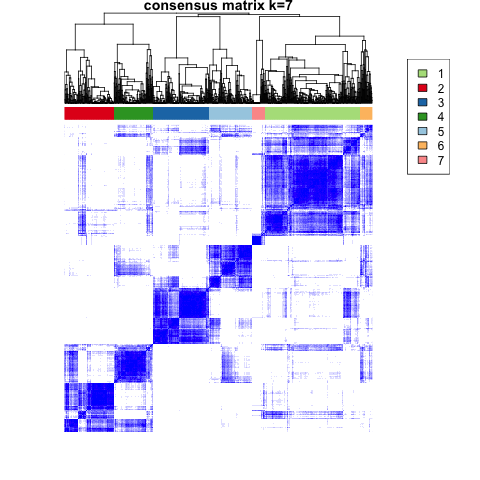

Supplement: Supporting Information 2 — The incrementally adjusting variable of clustering (k). [file 3423698.f2.zip › Supplementary Material 2/consensus007.png]

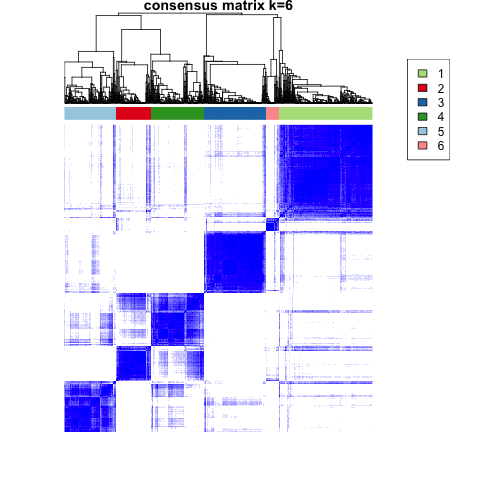

Supplement: Supporting Information 2 — The incrementally adjusting variable of clustering (k). [file 3423698.f2.zip › Supplementary Material 2/consensus006.png]

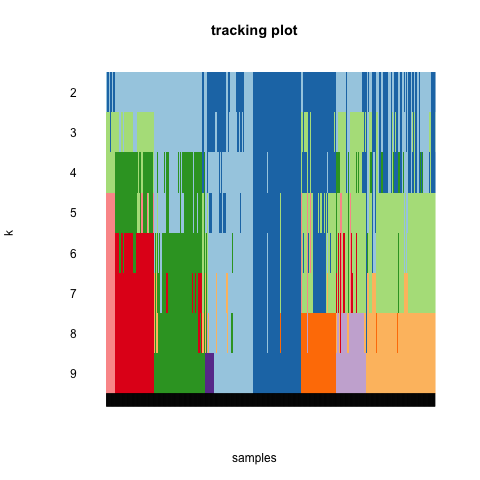

Supplement: Supporting Information 2 — The incrementally adjusting variable of clustering (k). [file 3423698.f2.zip › Supplementary Material 2/consensus012.png]

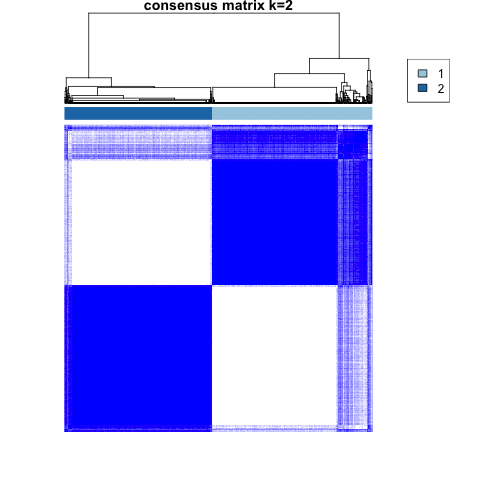

Supplement: Supporting Information 2 — The incrementally adjusting variable of clustering (k). [file 3423698.f2.zip › Supplementary Material 2/consensus002.png]

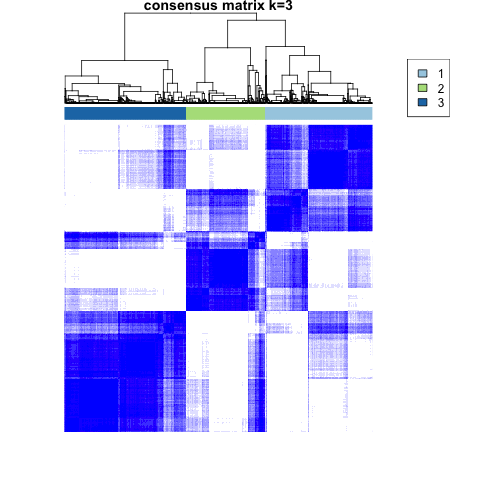

Supplement: Supporting Information 2 — The incrementally adjusting variable of clustering (k). [file 3423698.f2.zip › Supplementary Material 2/consensus003.png]

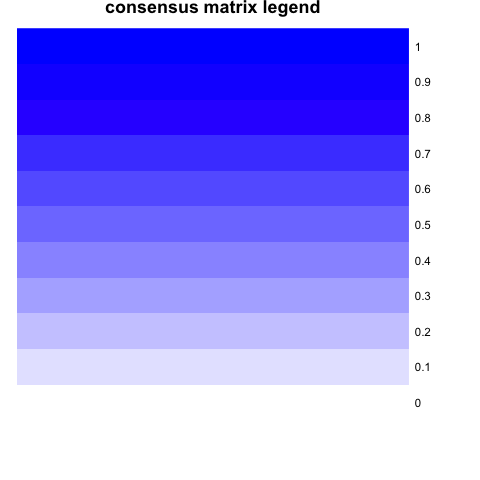

Supplement: Supporting Information 2 — The incrementally adjusting variable of clustering (k). [file 3423698.f2.zip › Supplementary Material 2/consensus001.png]

Risk 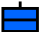 low 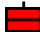 high

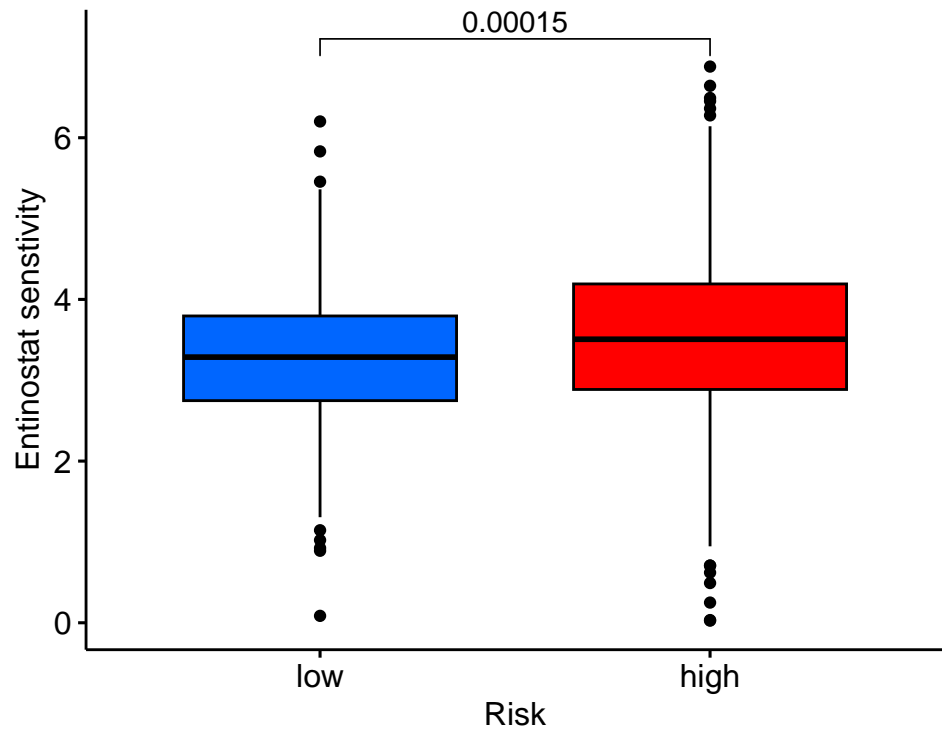

Supplement: Supporting Information 3 — Distinct patterns of drug sensitivity between low-risk and high-risk groups. [file 3423698.f3.zip › Supplementary Material 3/drugSenstivity.Entinostat.pdf]

Risk 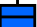 low 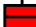 high

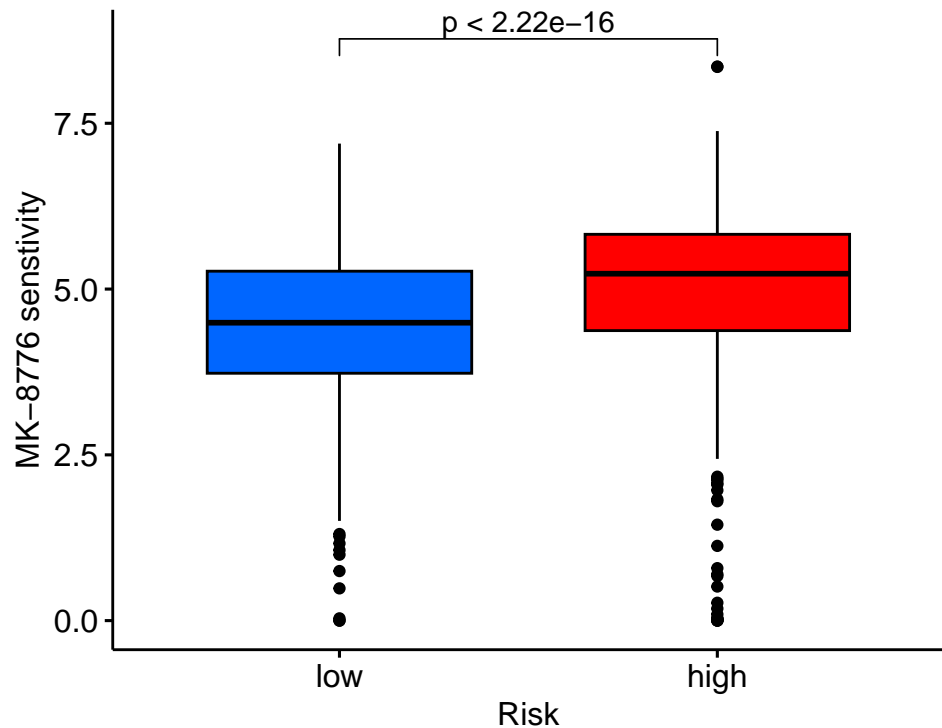

Supplement: Supporting Information 3 — Distinct patterns of drug sensitivity between low-risk and high-risk groups. [file 3423698.f3.zip › Supplementary Material 3/drugSenstivity.MK-8776.pdf]

Risk 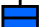 low 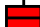 high

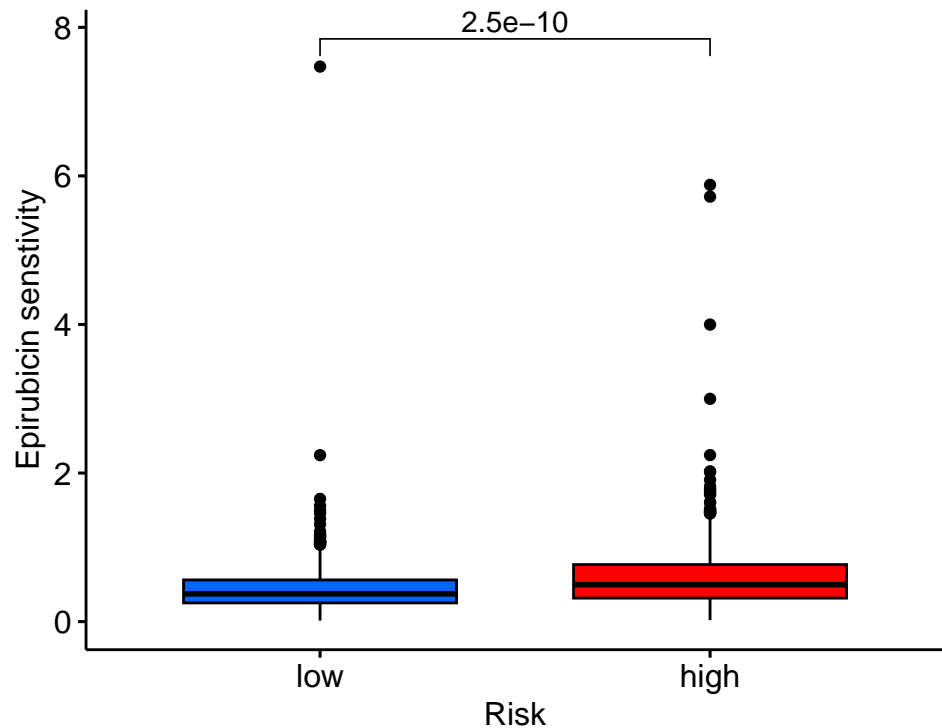

Supplement: Supporting Information 3 — Distinct patterns of drug sensitivity between low-risk and high-risk groups. [file 3423698.f3.zip › Supplementary Material 3/drugSenstivity.Epirubicin.pdf]

Risk 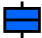 low 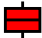 high

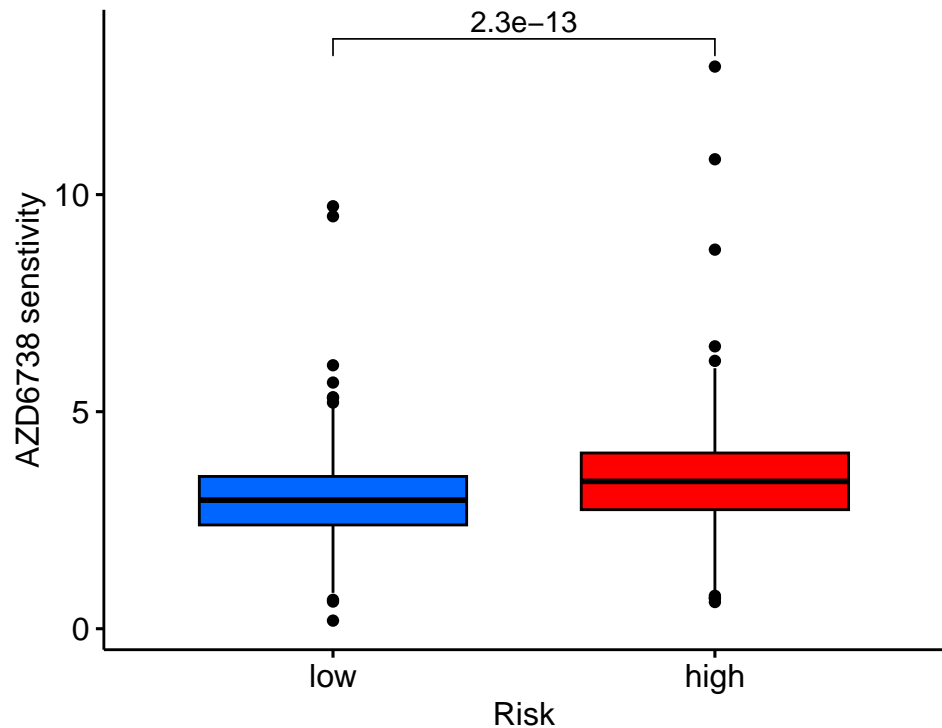

Supplement: Supporting Information 3 — Distinct patterns of drug sensitivity between low-risk and high-risk groups. [file 3423698.f3.zip › Supplementary Material 3/drugSenstivity.AZD6738.pdf]

Risk 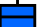 low 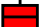 high

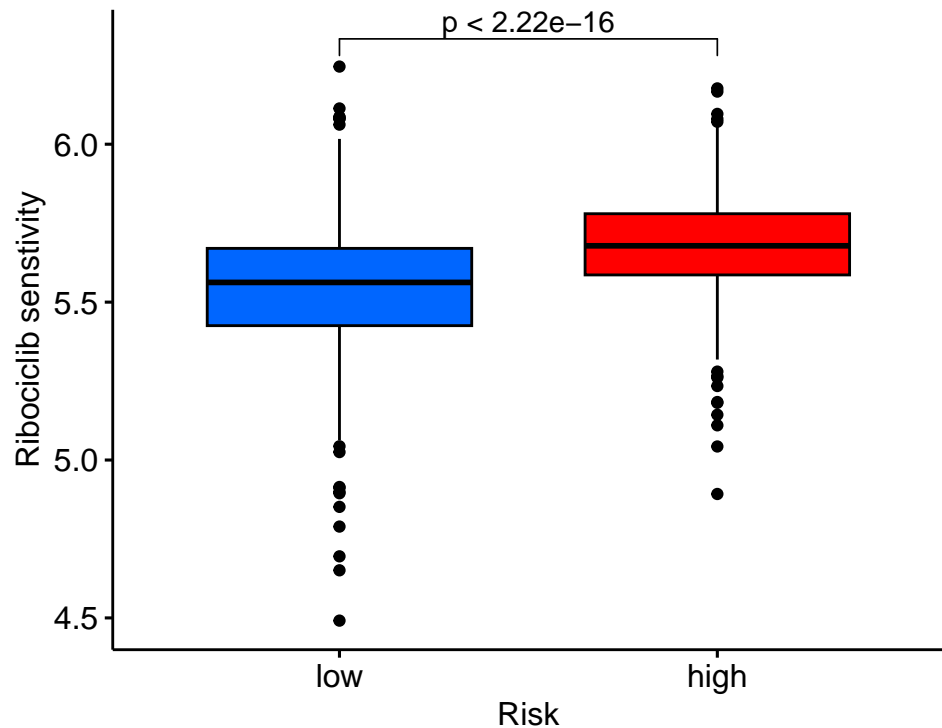

Supplement: Supporting Information 3 — Distinct patterns of drug sensitivity between low-risk and high-risk groups. [file 3423698.f3.zip › Supplementary Material 3/drugSenstivity.Ribociclib.pdf]

Risk 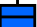 low 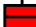 high

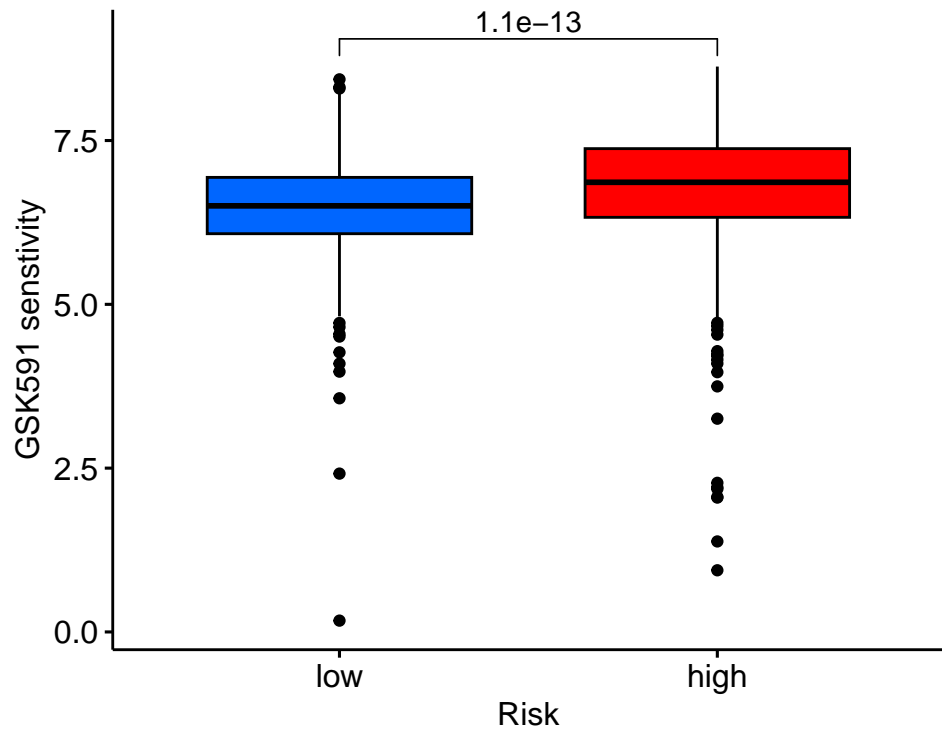

Supplement: Supporting Information 3 — Distinct patterns of drug sensitivity between low-risk and high-risk groups. [file 3423698.f3.zip › Supplementary Material 3/drugSenstivity.GSK591.pdf]

Risk 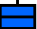 low 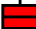 high

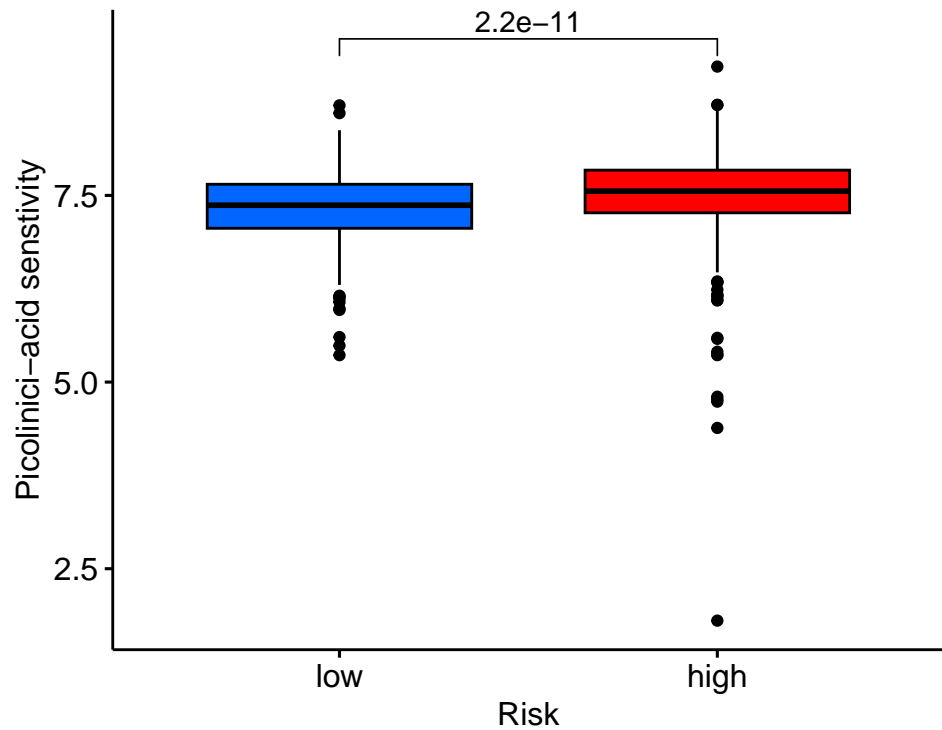

Supplement: Supporting Information 3 — Distinct patterns of drug sensitivity between low-risk and high-risk groups. [file 3423698.f3.zip › Supplementary Material 3/drugSenstivity.Picolinici-acid.pdf]

Risk 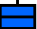 low 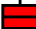 high

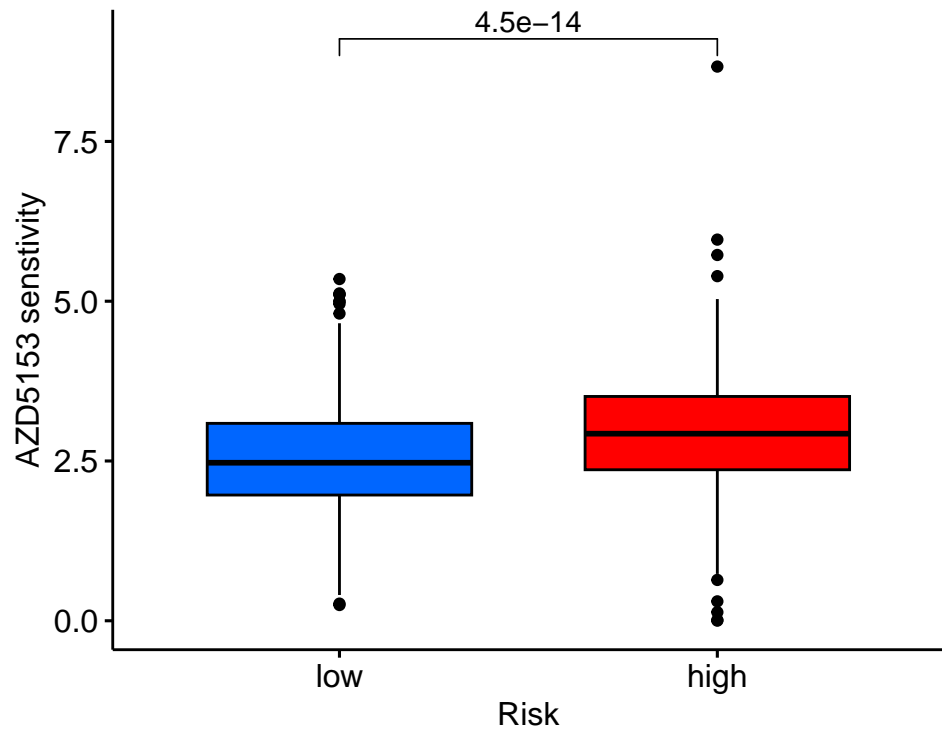

Supplement: Supporting Information 3 — Distinct patterns of drug sensitivity between low-risk and high-risk groups. [file 3423698.f3.zip › Supplementary Material 3/drugSenstivity.AZD5153.pdf]

Risk 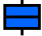 low 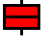 high

$p < 2.22e-16$

PFI3 sensitivity

10

8

6

low

high

Risk

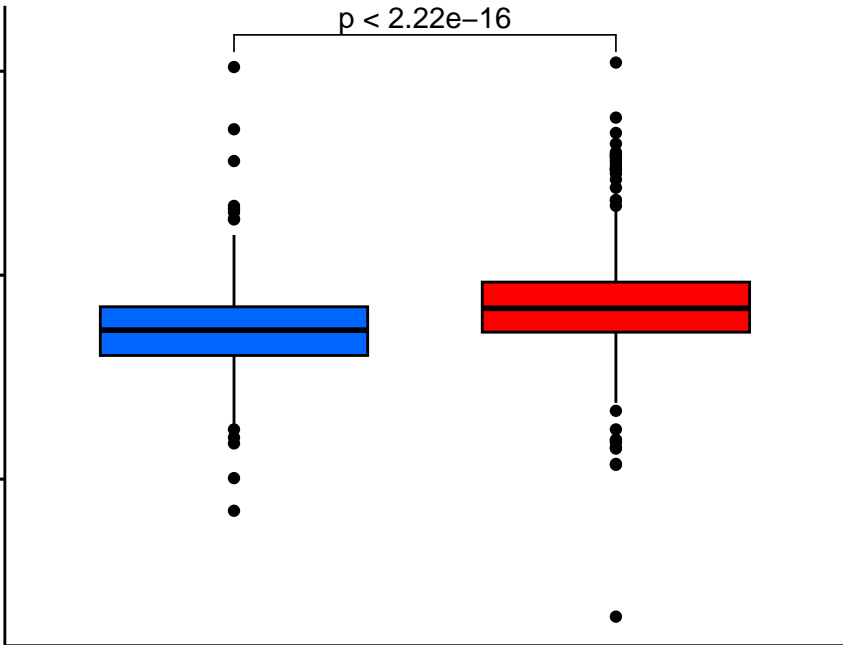

Supplement: Supporting Information 3 — Distinct patterns of drug sensitivity between low-risk and high-risk groups. [file 3423698.f3.zip › Supplementary Material 3/drugSenstivity.PFI3.pdf]

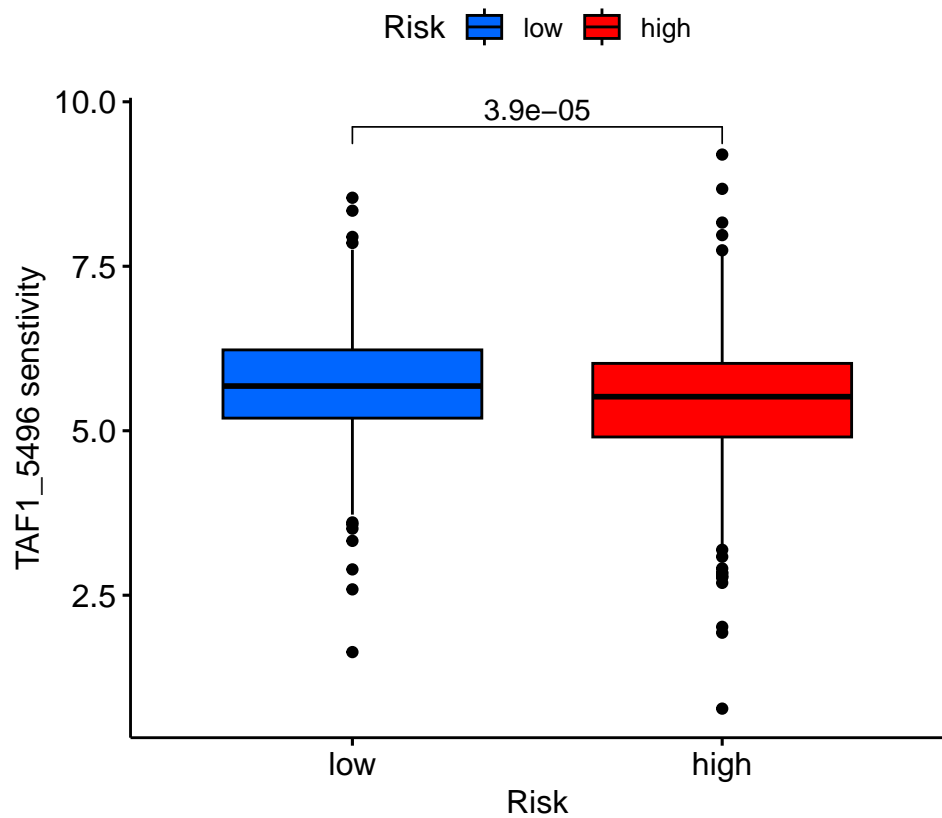

Supplement: Supporting Information 3 — Distinct patterns of drug sensitivity between low-risk and high-risk groups. [file 3423698.f3.zip › Supplementary Material 3/drugSenstivity.TAF1_5496.pdf]

Risk 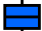 low 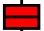 high

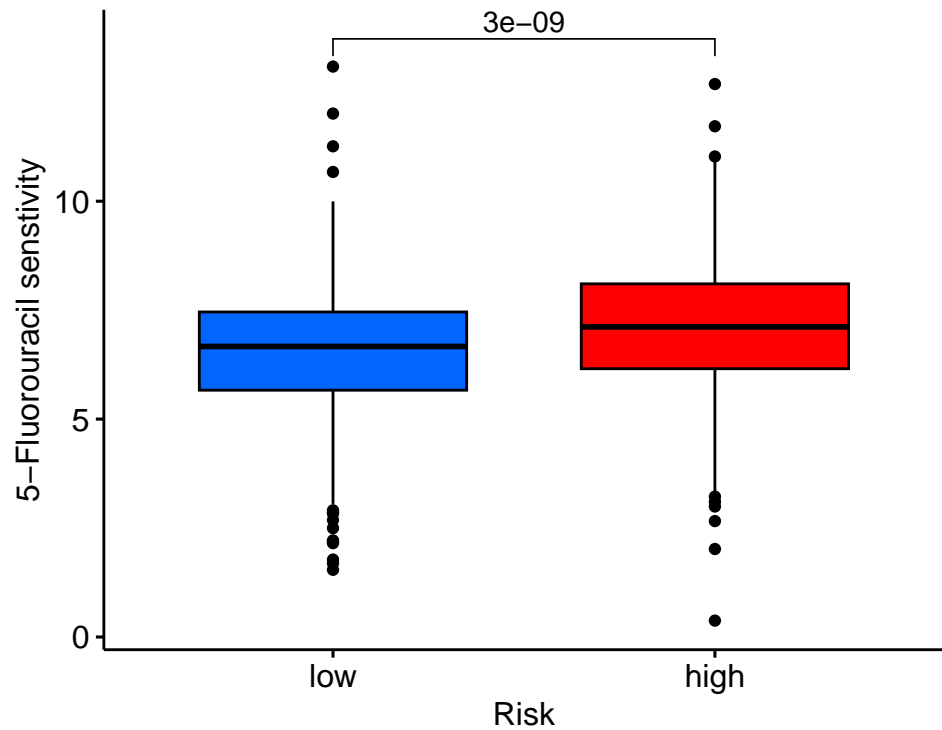

Supplement: Supporting Information 3 — Distinct patterns of drug sensitivity between low-risk and high-risk groups. [file 3423698.f3.zip › Supplementary Material 3/drugSenstivity.5-Fluorouracil.pdf]

Risk 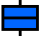 low 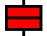 high

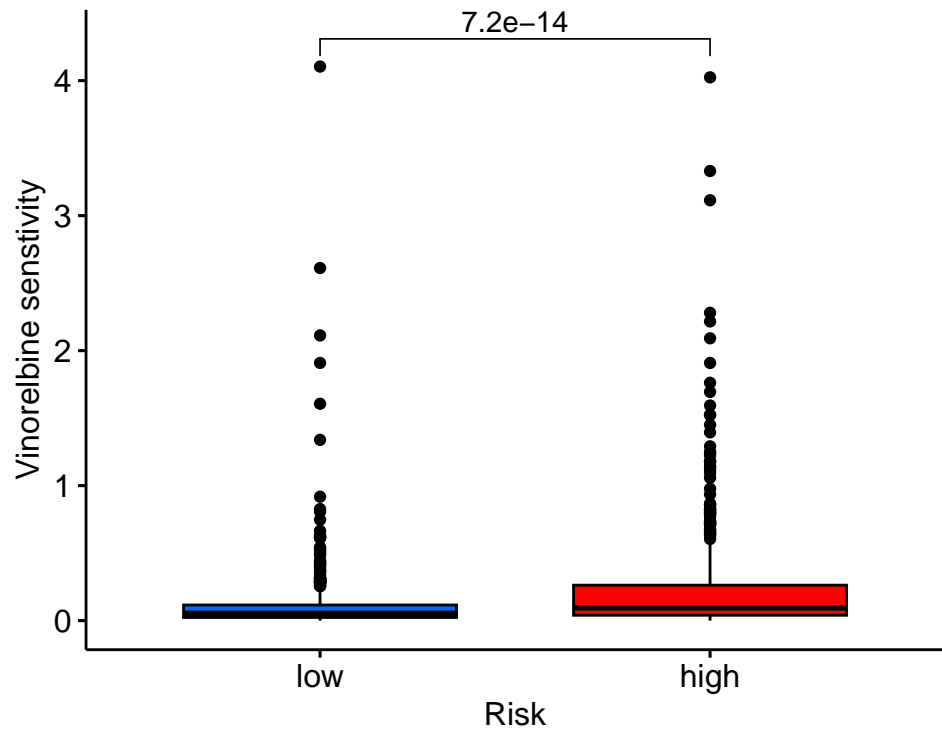

Supplement: Supporting Information 3 — Distinct patterns of drug sensitivity between low-risk and high-risk groups. [file 3423698.f3.zip › Supplementary Material 3/drugSenstivity.Vinorelbine.pdf]

Risk 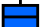 low 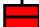 high

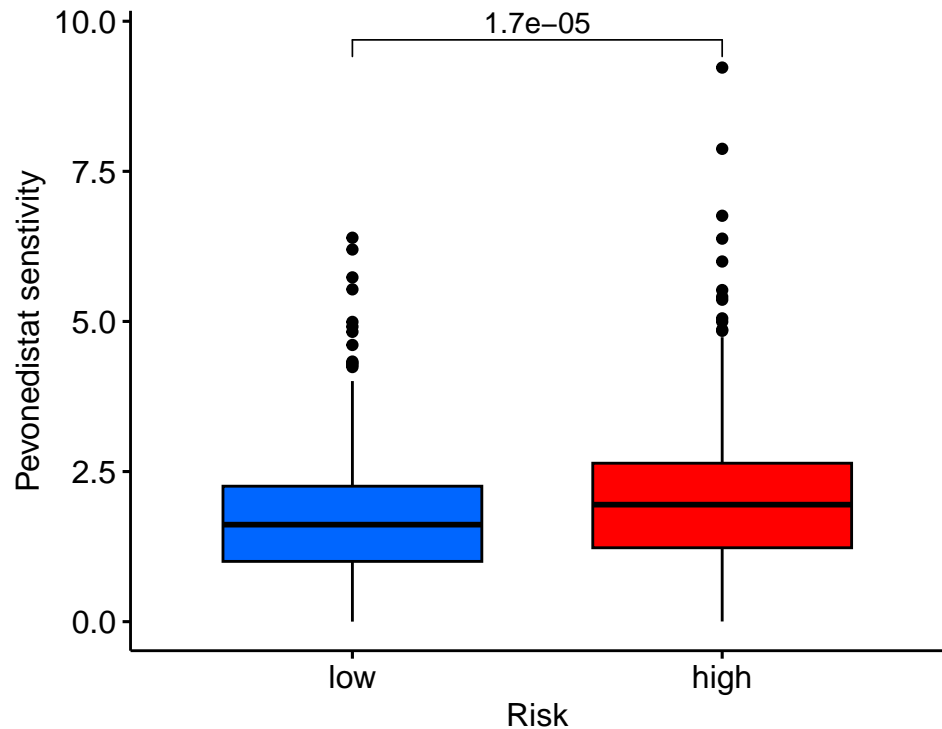

Supplement: Supporting Information 3 — Distinct patterns of drug sensitivity between low-risk and high-risk groups. [file 3423698.f3.zip › Supplementary Material 3/drugSenstivity.Pevonedistat.pdf]

Risk low high

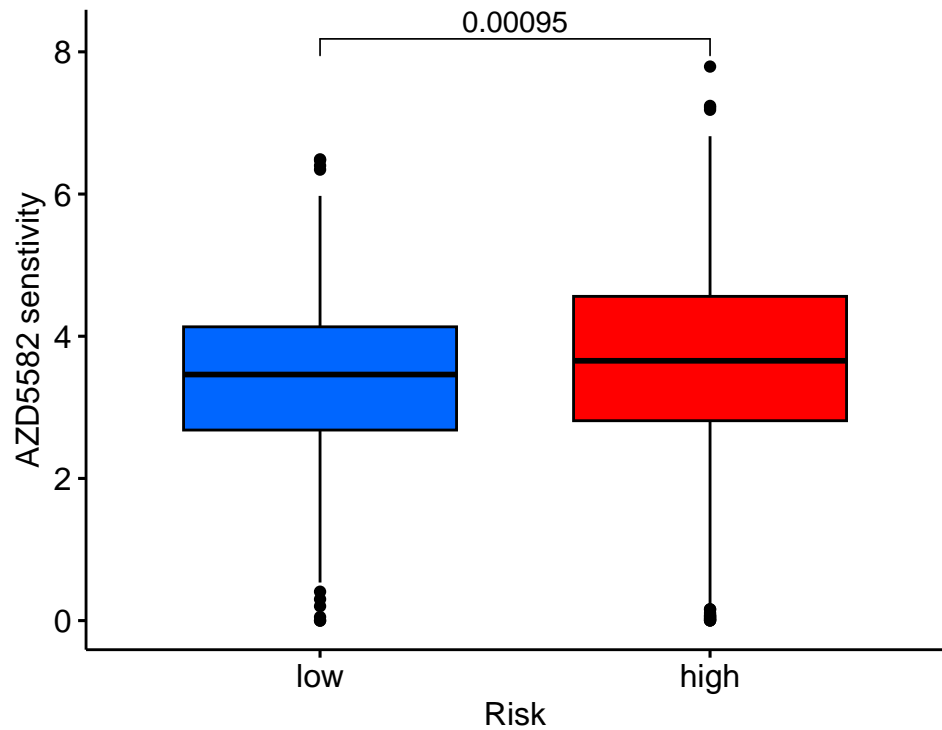

Supplement: Supporting Information 3 — Distinct patterns of drug sensitivity between low-risk and high-risk groups. [file 3423698.f3.zip › Supplementary Material 3/drugSenstivity.AZD5582.pdf]

Risk 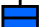 low 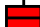 high

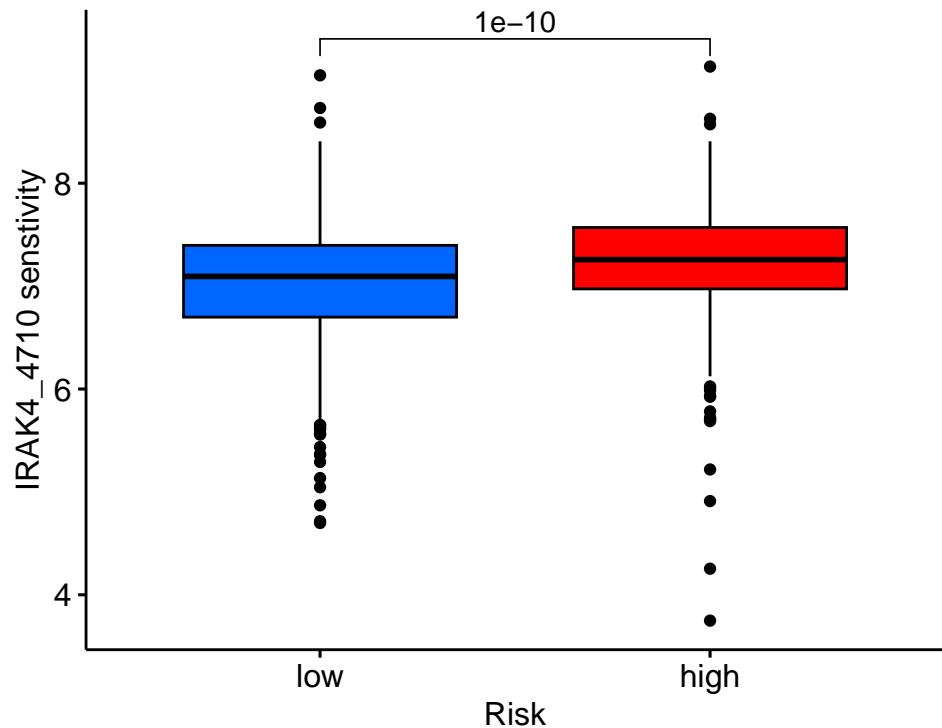

Supplement: Supporting Information 3 — Distinct patterns of drug sensitivity between low-risk and high-risk groups. [file 3423698.f3.zip › Supplementary Material 3/drugSenstivity.IRAK4_4710.pdf]

Risk low high

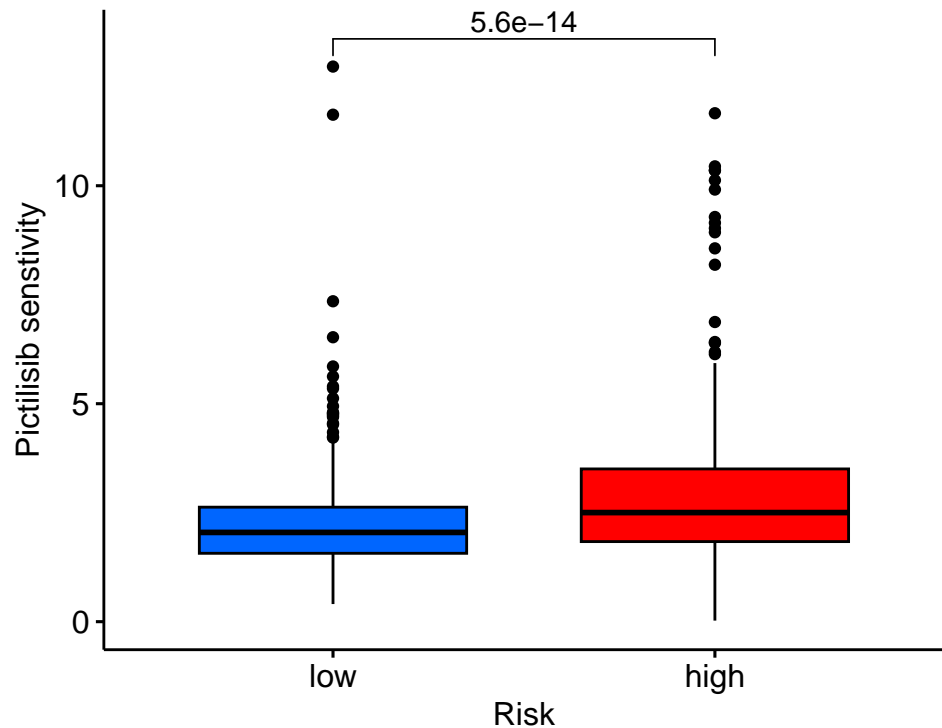

Supplement: Supporting Information 3 — Distinct patterns of drug sensitivity between low-risk and high-risk groups. [file 3423698.f3.zip › Supplementary Material 3/drugSenstivity.Pictilisib.pdf]

Risk 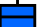 low 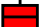 high

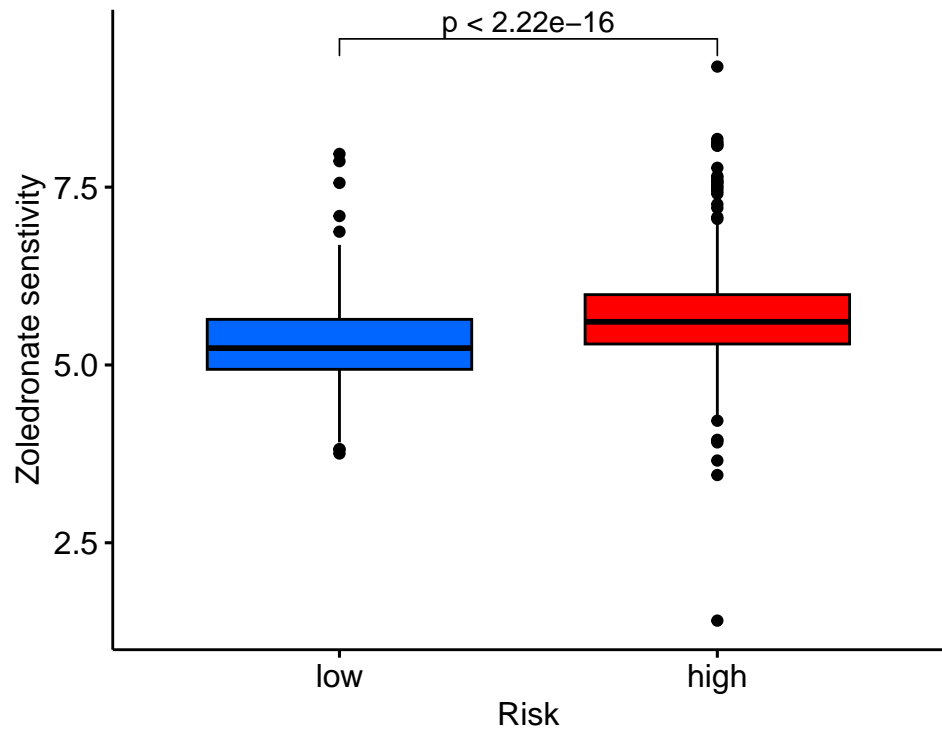

Supplement: Supporting Information 3 — Distinct patterns of drug sensitivity between low-risk and high-risk groups. [file 3423698.f3.zip › Supplementary Material 3/drugSenstivity.Zoledronate.pdf]

Risk 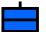 low 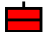 high

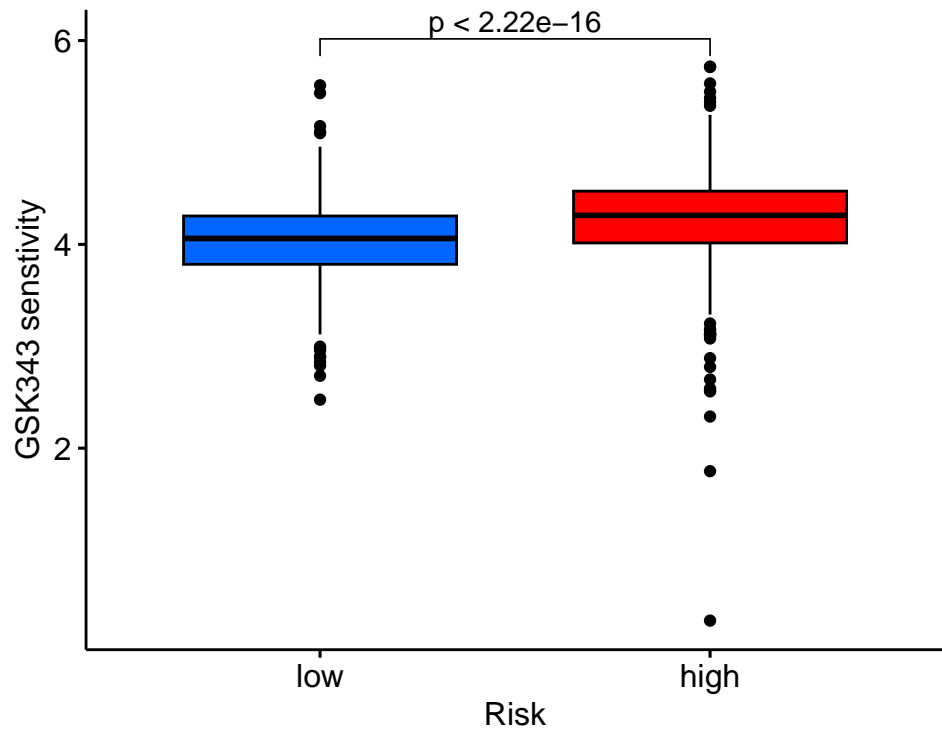

Supplement: Supporting Information 3 — Distinct patterns of drug sensitivity between low-risk and high-risk groups. [file 3423698.f3.zip › Supplementary Material 3/drugSenstivity.GSK343.pdf]

Risk 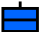 low 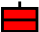 high

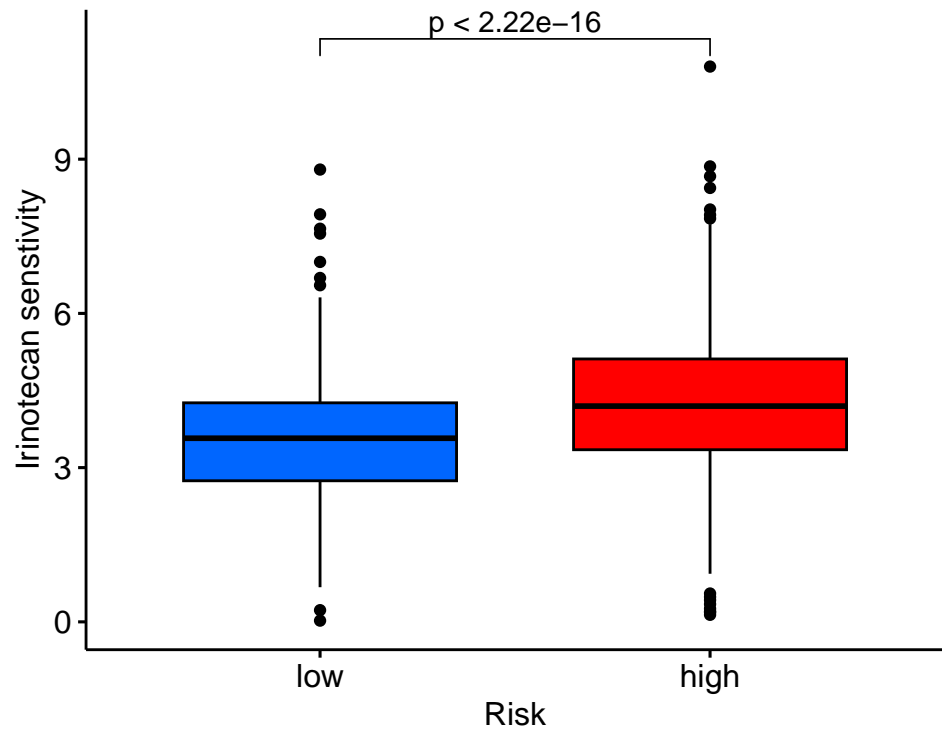

Supplement: Supporting Information 3 — Distinct patterns of drug sensitivity between low-risk and high-risk groups. [file 3423698.f3.zip › Supplementary Material 3/drugSenstivity.Irinotecan.pdf]

Risk 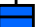 low 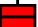 high

$p < 2.22e-16$

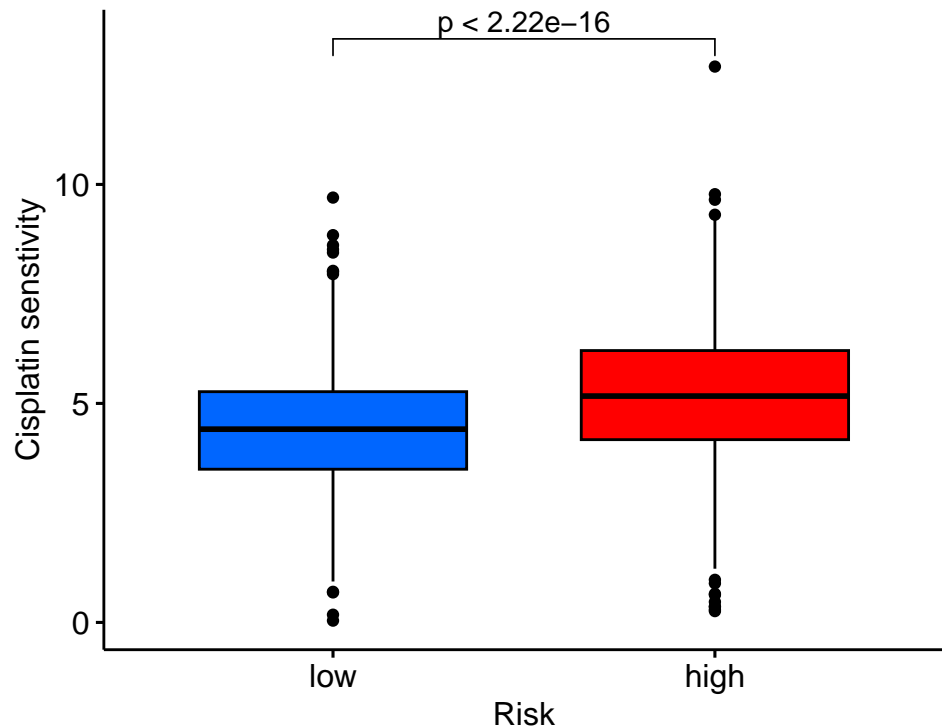

Supplement: Supporting Information 3 — Distinct patterns of drug sensitivity between low-risk and high-risk groups. [file 3423698.f3.zip › Supplementary Material 3/drugSenstivity.Cisplatin.pdf]

Risk 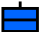 low 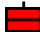 high

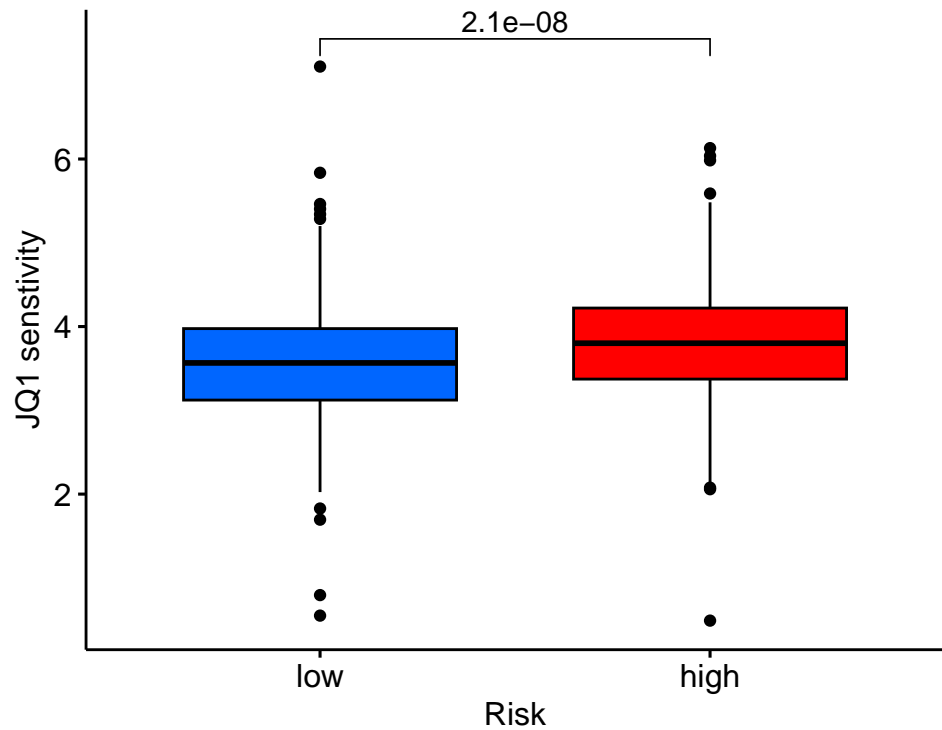

Supplement: Supporting Information 3 — Distinct patterns of drug sensitivity between low-risk and high-risk groups. [file 3423698.f3.zip › Supplementary Material 3/drugSenstivity.JQ1.pdf]

Risk 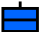 low 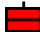 high

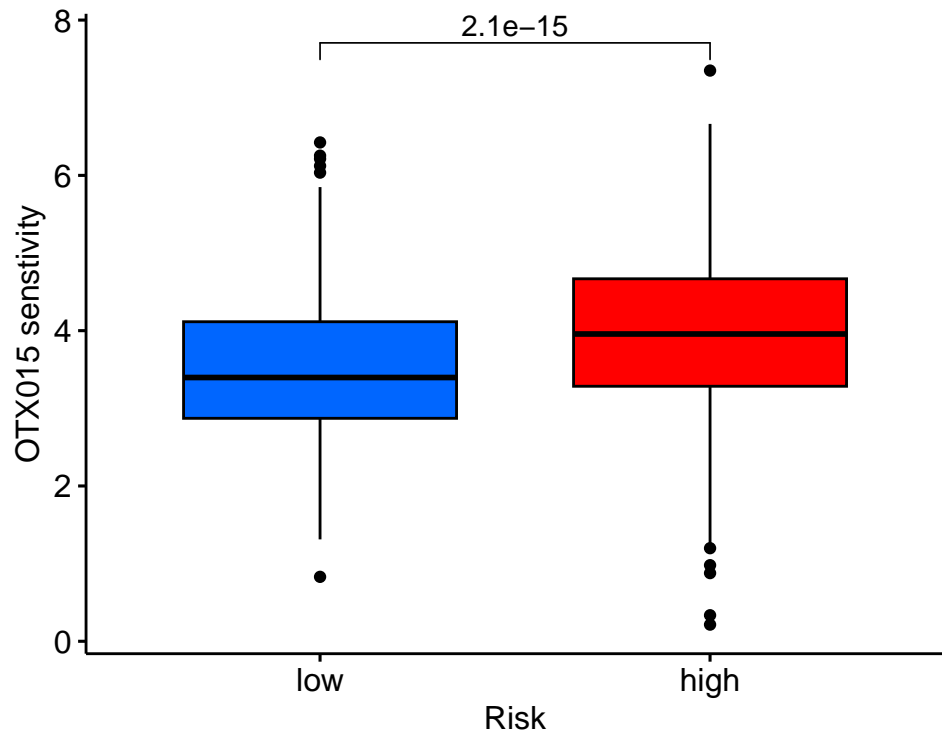

Supplement: Supporting Information 3 — Distinct patterns of drug sensitivity between low-risk and high-risk groups. [file 3423698.f3.zip › Supplementary Material 3/drugSenstivity.OTX015.pdf]

Risk 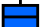 low 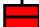 high

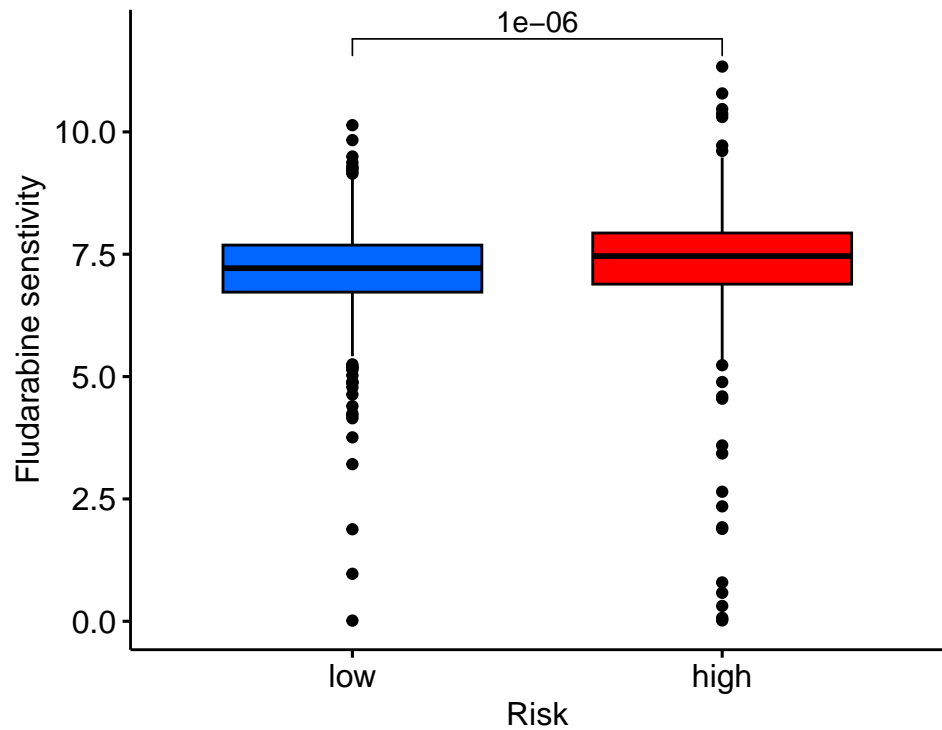

Supplement: Supporting Information 3 — Distinct patterns of drug sensitivity between low-risk and high-risk groups. [file 3423698.f3.zip › Supplementary Material 3/drugSenstivity.Fludarabine.pdf]

Risk 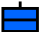 low 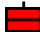 high

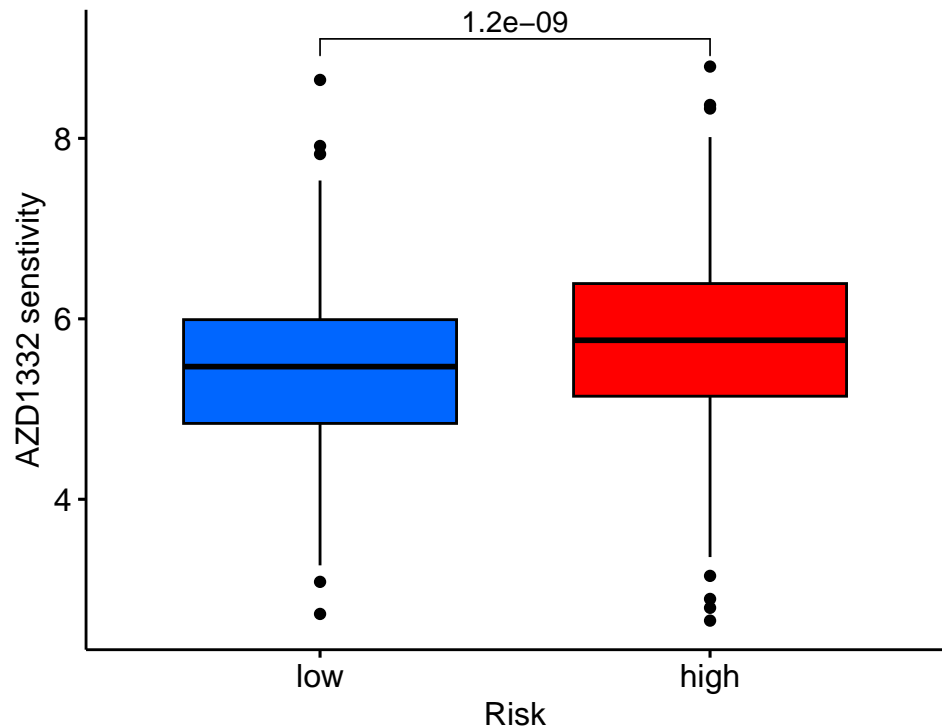

Supplement: Supporting Information 3 — Distinct patterns of drug sensitivity between low-risk and high-risk groups. [file 3423698.f3.zip › Supplementary Material 3/drugSenstivity.AZD1332.pdf]

Risk 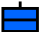 low 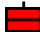 high

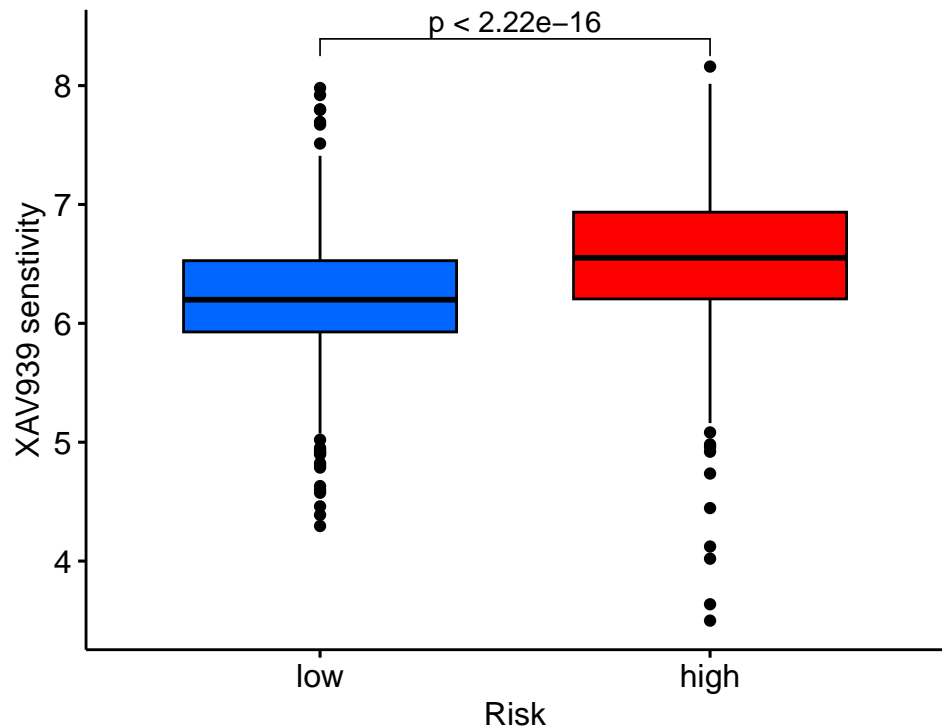

Supplement: Supporting Information 3 — Distinct patterns of drug sensitivity between low-risk and high-risk groups. [file 3423698.f3.zip › Supplementary Material 3/drugSenstivity.XAV939.pdf]

Risk 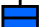 low 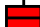 high

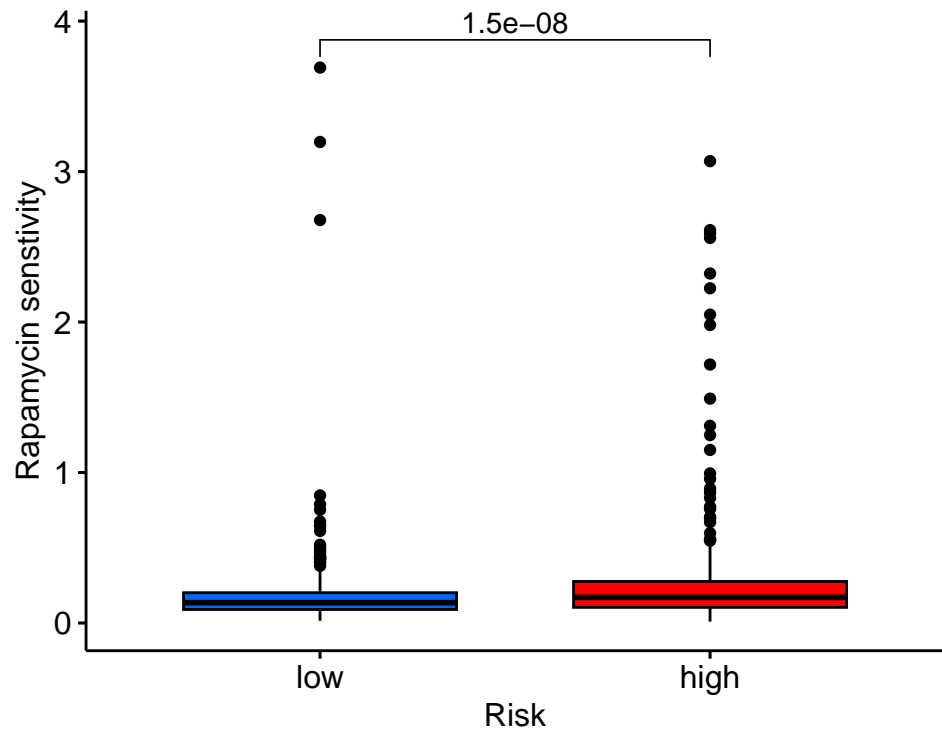

Supplement: Supporting Information 3 — Distinct patterns of drug sensitivity between low-risk and high-risk groups. [file 3423698.f3.zip › Supplementary Material 3/drugSenstivity.Rapamycin.pdf]

Risk 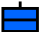 low 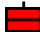 high

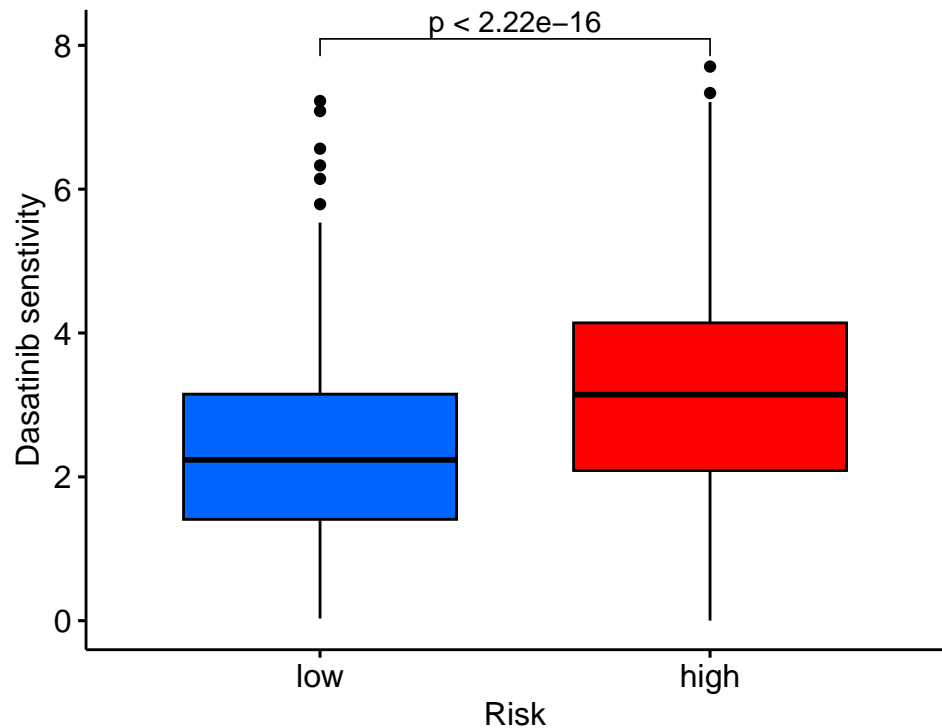

Supplement: Supporting Information 3 — Distinct patterns of drug sensitivity between low-risk and high-risk groups. [file 3423698.f3.zip › Supplementary Material 3/drugSenstivity.Dasatinib.pdf]

Risk 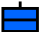 low 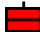 high

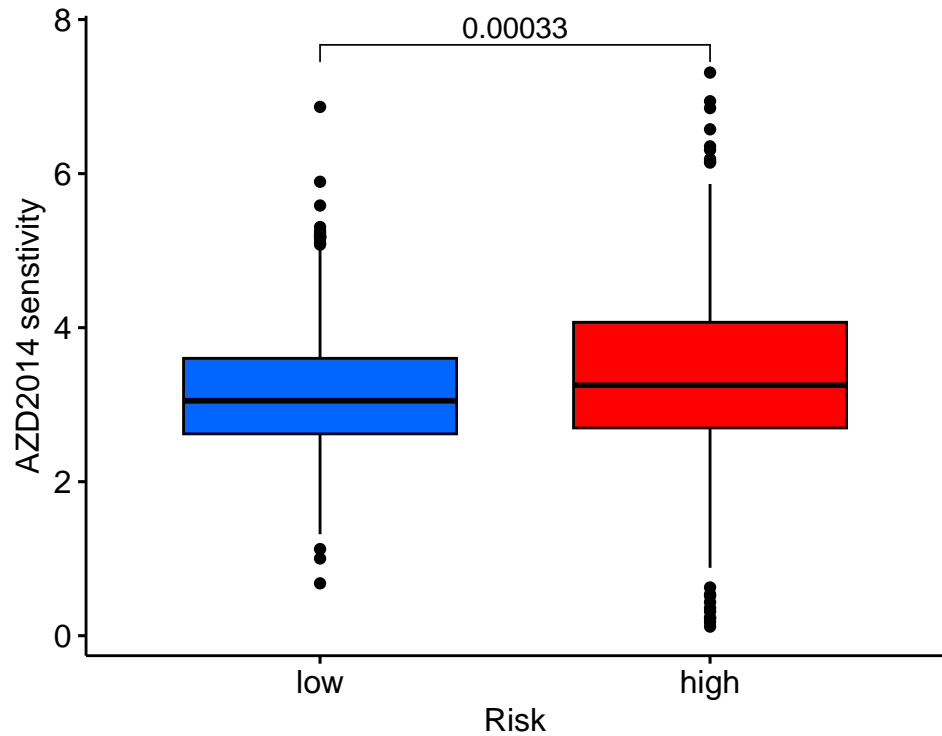

Supplement: Supporting Information 3 — Distinct patterns of drug sensitivity between low-risk and high-risk groups. [file 3423698.f3.zip › Supplementary Material 3/drugSenstivity.AZD2014.pdf]

Risk 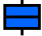 low 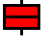 high

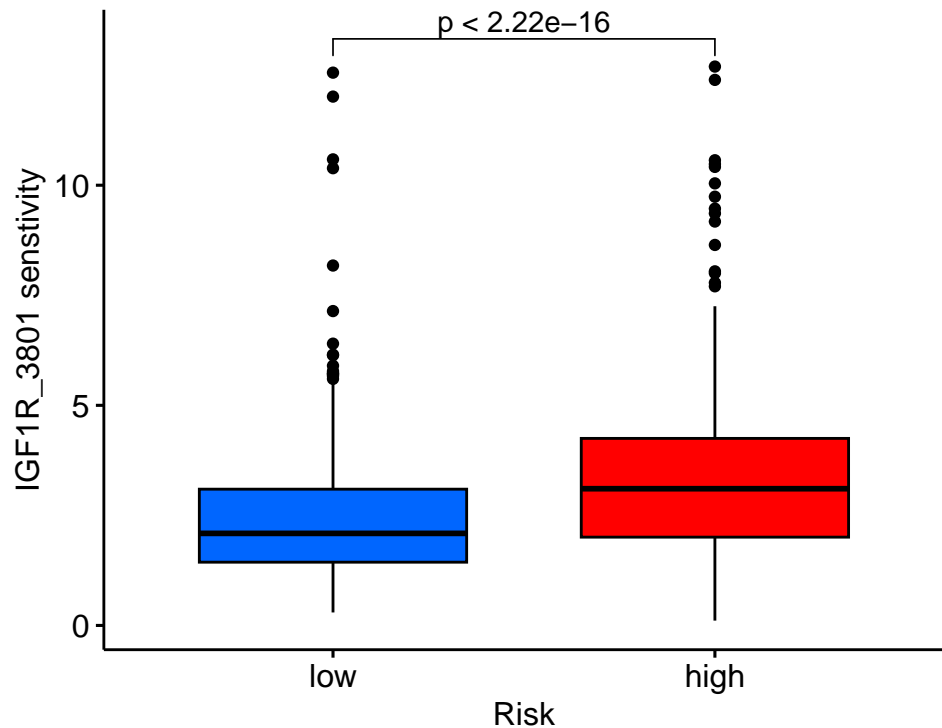

Supplement: Supporting Information 3 — Distinct patterns of drug sensitivity between low-risk and high-risk groups. [file 3423698.f3.zip › Supplementary Material 3/drugSenstivity.IGF1R_3801.pdf]

Risk 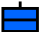 low 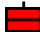 high

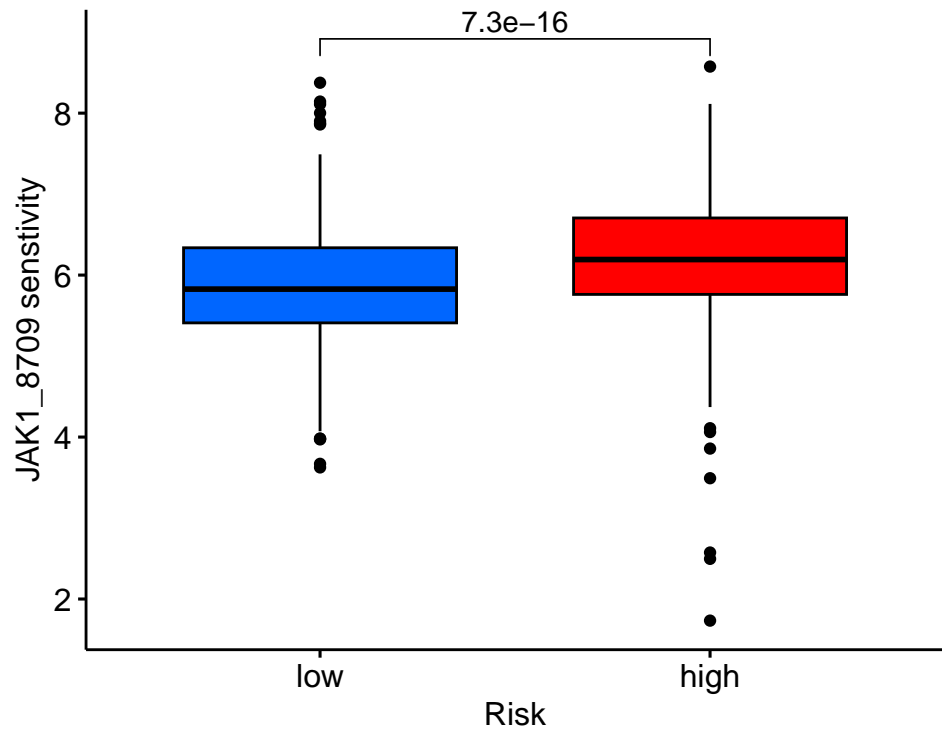

Supplement: Supporting Information 3 — Distinct patterns of drug sensitivity between low-risk and high-risk groups. [file 3423698.f3.zip › Supplementary Material 3/drugSenstivity.JAK1_8709.pdf]

Risk low high

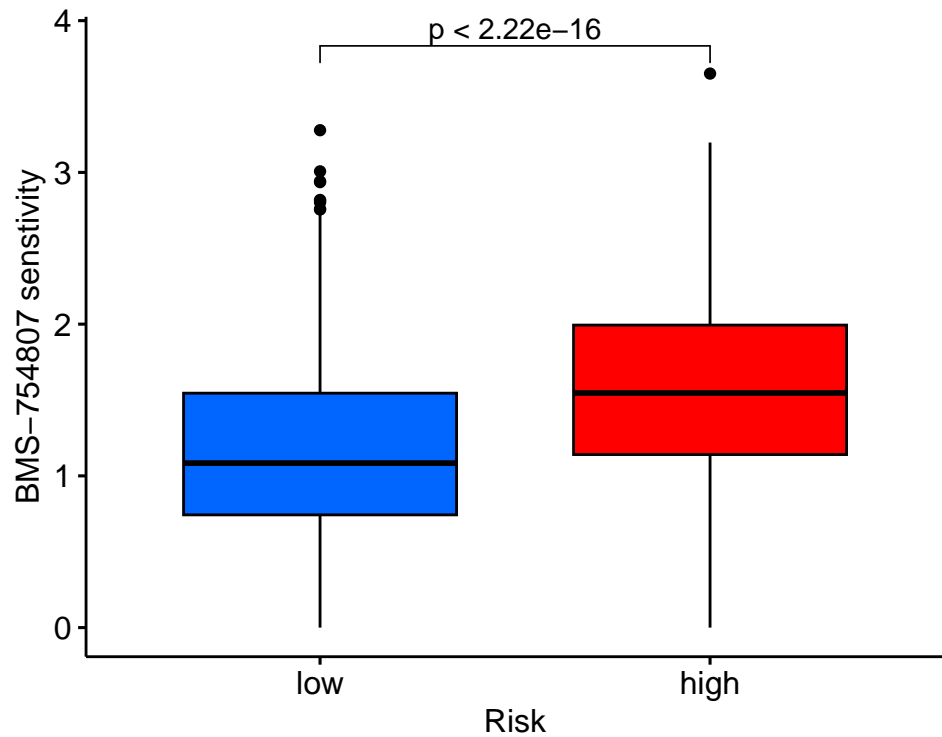

Supplement: Supporting Information 3 — Distinct patterns of drug sensitivity between low-risk and high-risk groups. [file 3423698.f3.zip › Supplementary Material 3/drugSenstivity.BMS-754807.pdf]

Risk 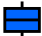 low 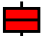 high

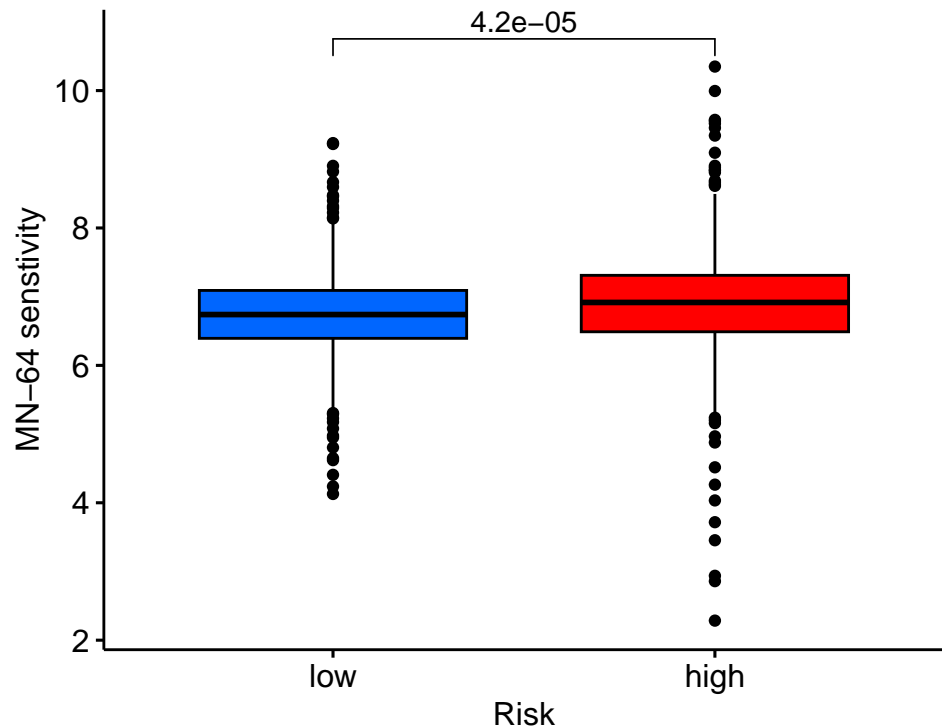

Supplement: Supporting Information 3 — Distinct patterns of drug sensitivity between low-risk and high-risk groups. [file 3423698.f3.zip › Supplementary Material 3/drugSenstivity.MN-64.pdf]

Risk 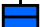 low 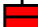 high

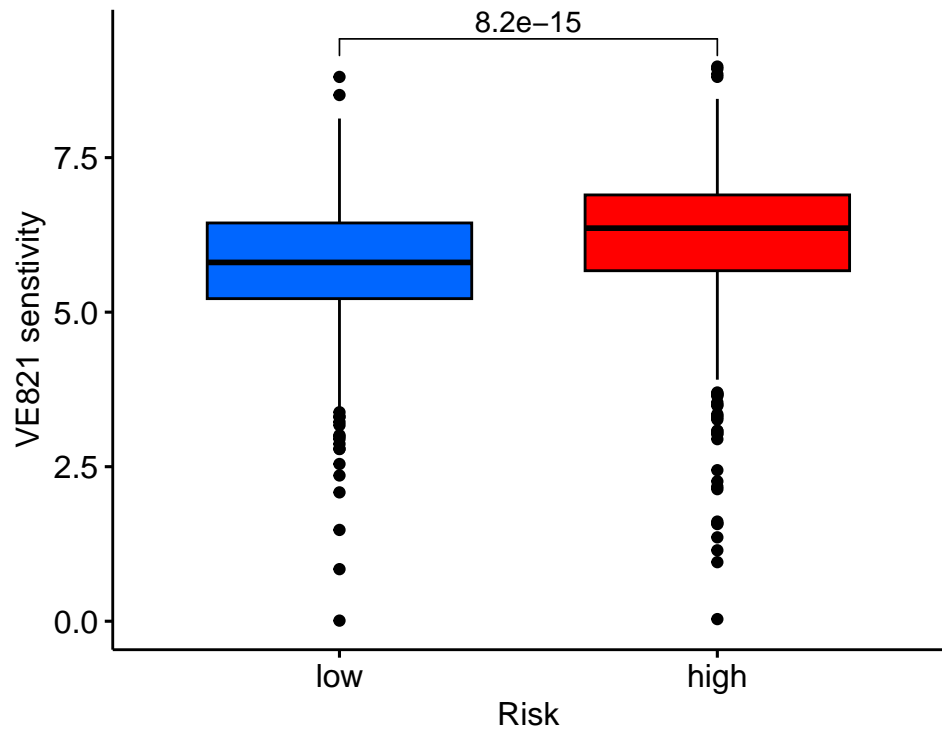

Supplement: Supporting Information 3 — Distinct patterns of drug sensitivity between low-risk and high-risk groups. [file 3423698.f3.zip › Supplementary Material 3/drugSenstivity.VE821.pdf]

Risk 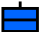 low 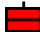 high

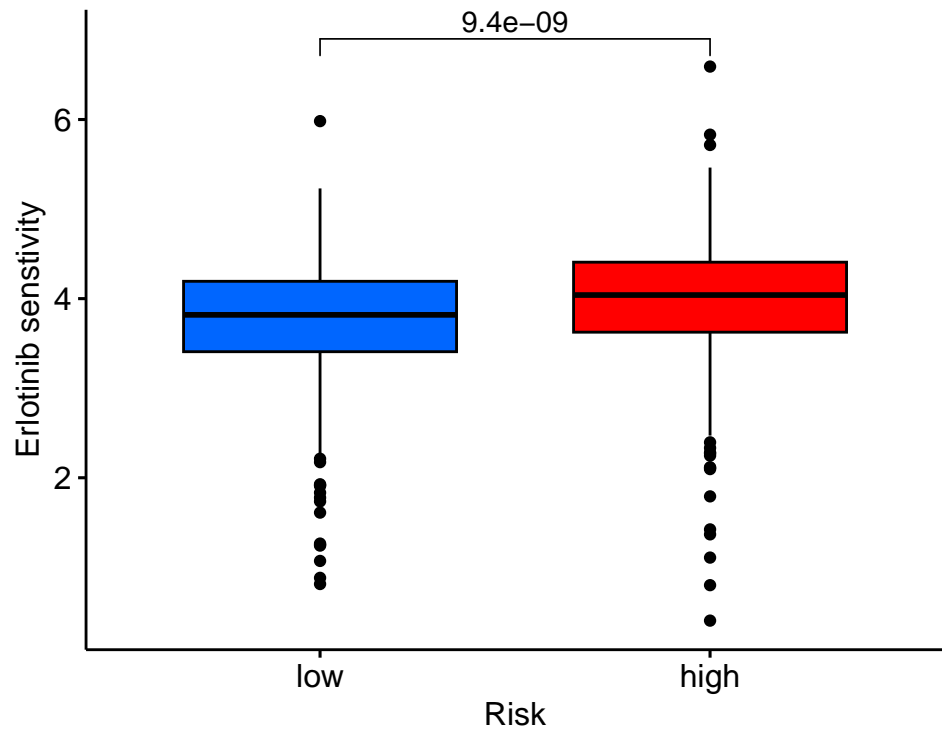

Supplement: Supporting Information 3 — Distinct patterns of drug sensitivity between low-risk and high-risk groups. [file 3423698.f3.zip › Supplementary Material 3/drugSenstivity.Erlotinib.pdf]

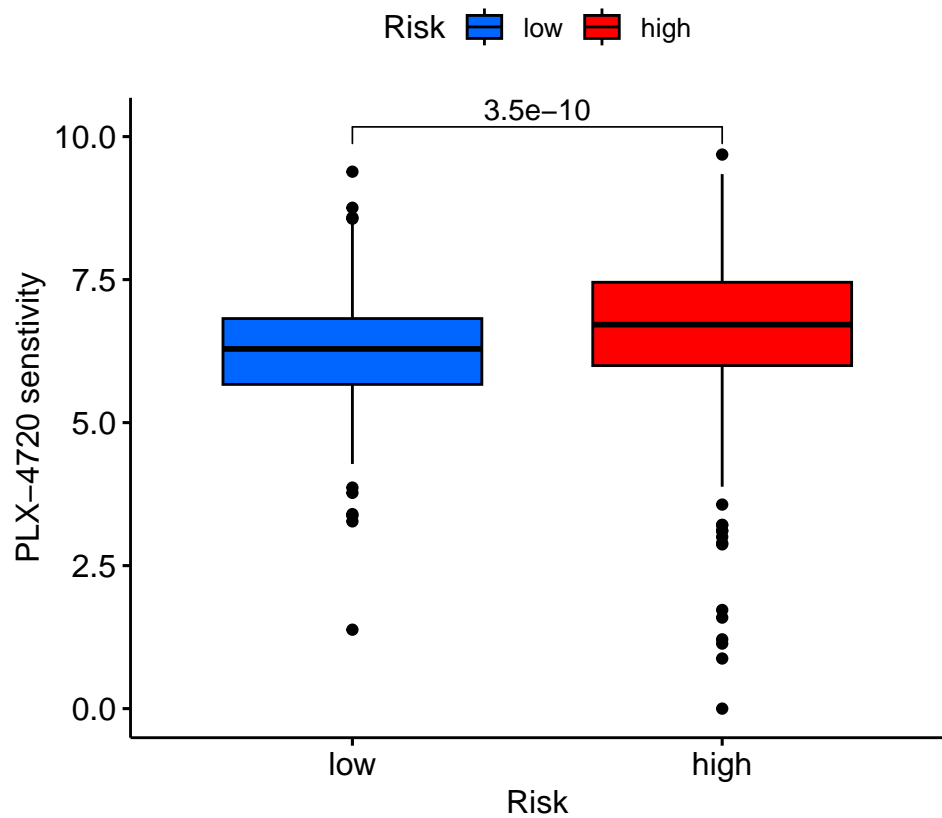

Supplement: Supporting Information 3 — Distinct patterns of drug sensitivity between low-risk and high-risk groups. [file 3423698.f3.zip › Supplementary Material 3/drugSenstivity.PLX-4720.pdf]

Risk 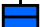 low 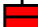 high

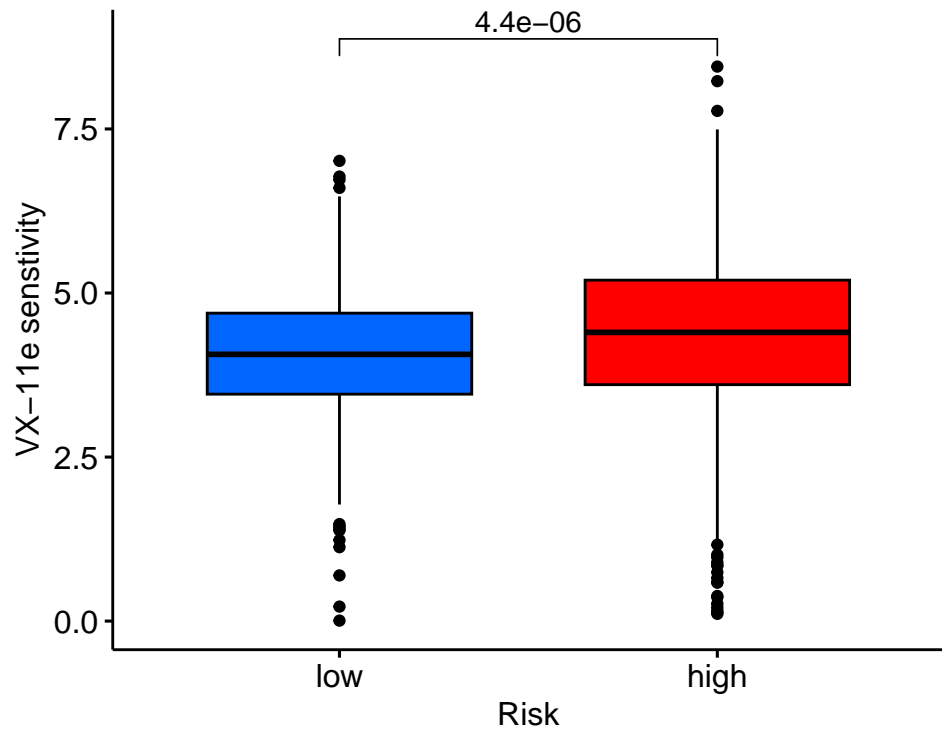

Supplement: Supporting Information 3 — Distinct patterns of drug sensitivity between low-risk and high-risk groups. [file 3423698.f3.zip › Supplementary Material 3/drugSenstivity.VX-11e.pdf]

Risk 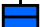 low 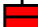 high

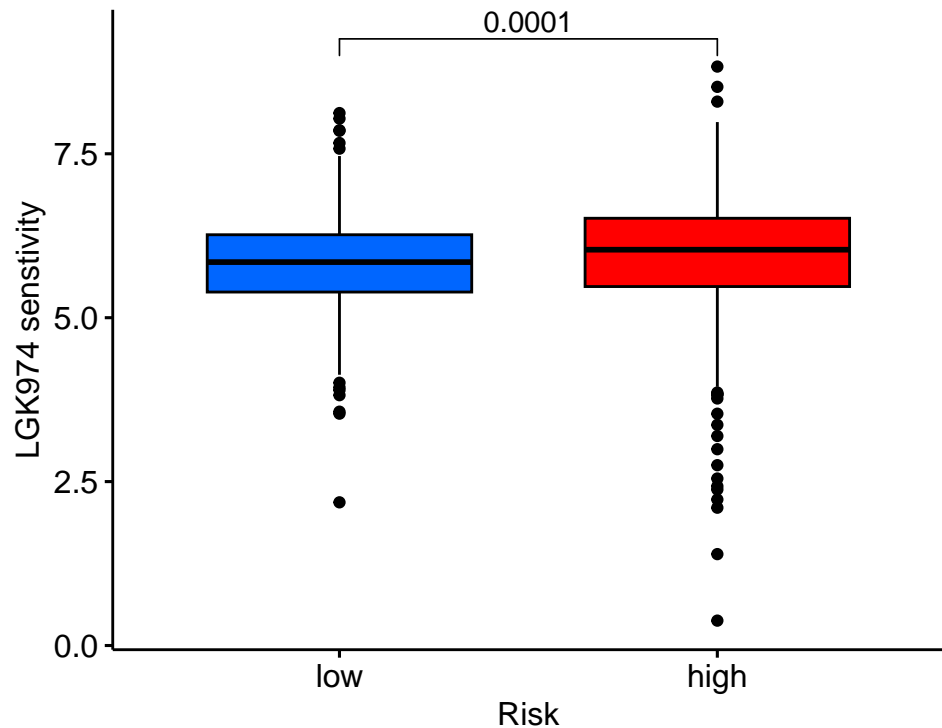

Supplement: Supporting Information 3 — Distinct patterns of drug sensitivity between low-risk and high-risk groups. [file 3423698.f3.zip › Supplementary Material 3/drugSenstivity.LGK974.pdf]

Risk 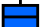 low 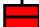 high

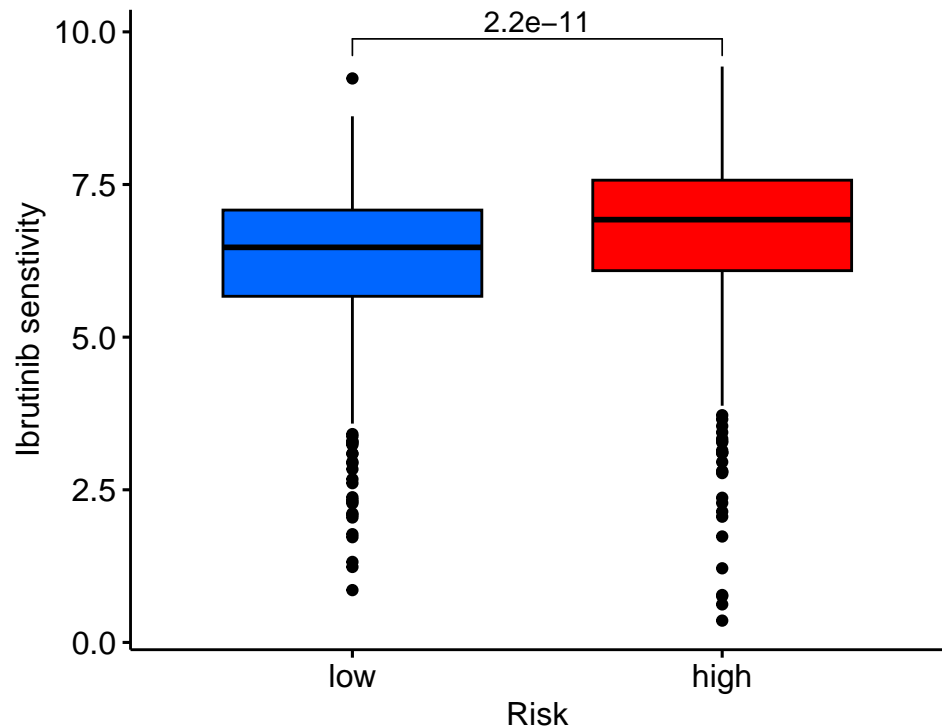

Supplement: Supporting Information 3 — Distinct patterns of drug sensitivity between low-risk and high-risk groups. [file 3423698.f3.zip › Supplementary Material 3/drugSenstivity.Ibrutinib.pdf]

Risk 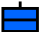 low 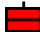 high

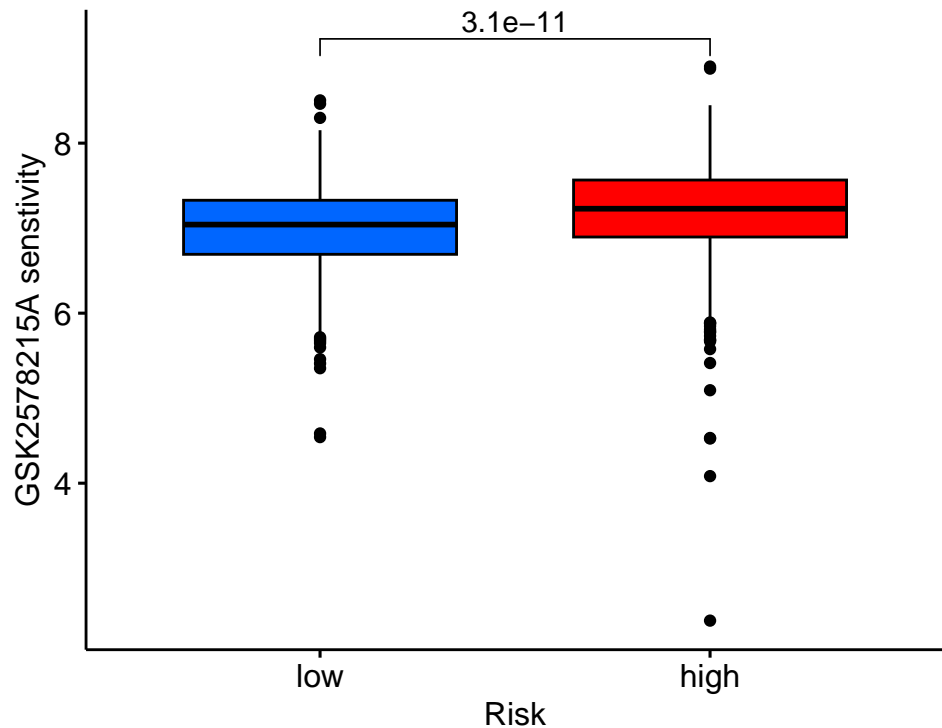

Supplement: Supporting Information 3 — Distinct patterns of drug sensitivity between low-risk and high-risk groups. [file 3423698.f3.zip › Supplementary Material 3/drugSenstivity.GSK2578215A.pdf]

Risk 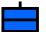 low 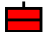 high

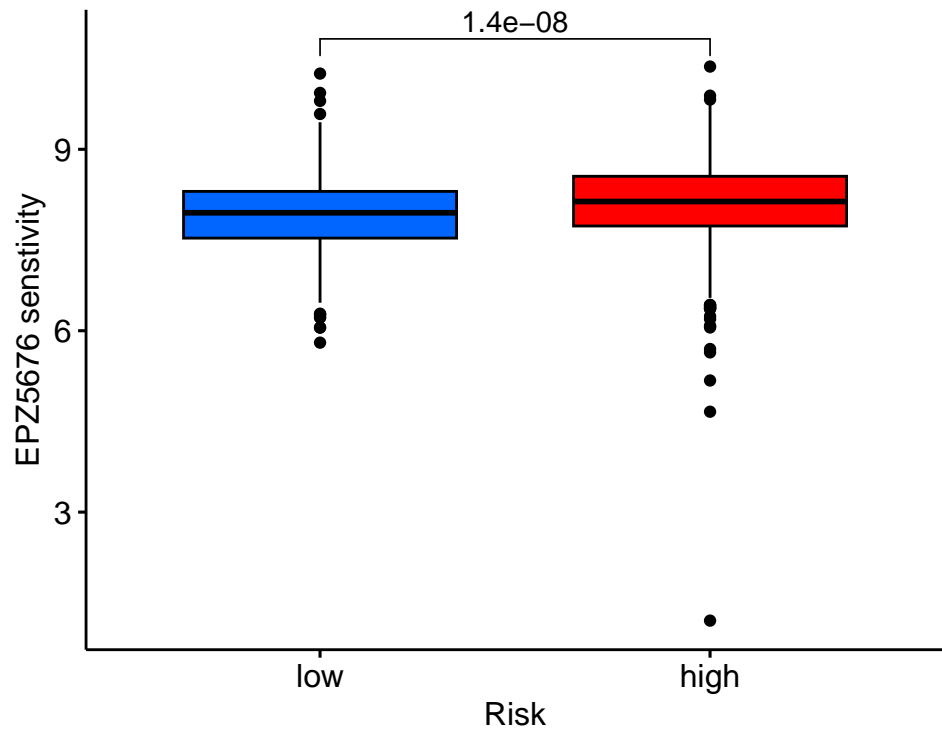

Supplement: Supporting Information 3 — Distinct patterns of drug sensitivity between low-risk and high-risk groups. [file 3423698.f3.zip › Supplementary Material 3/drugSenstivity.EPZ5676.pdf]

Risk 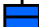 low 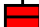 high

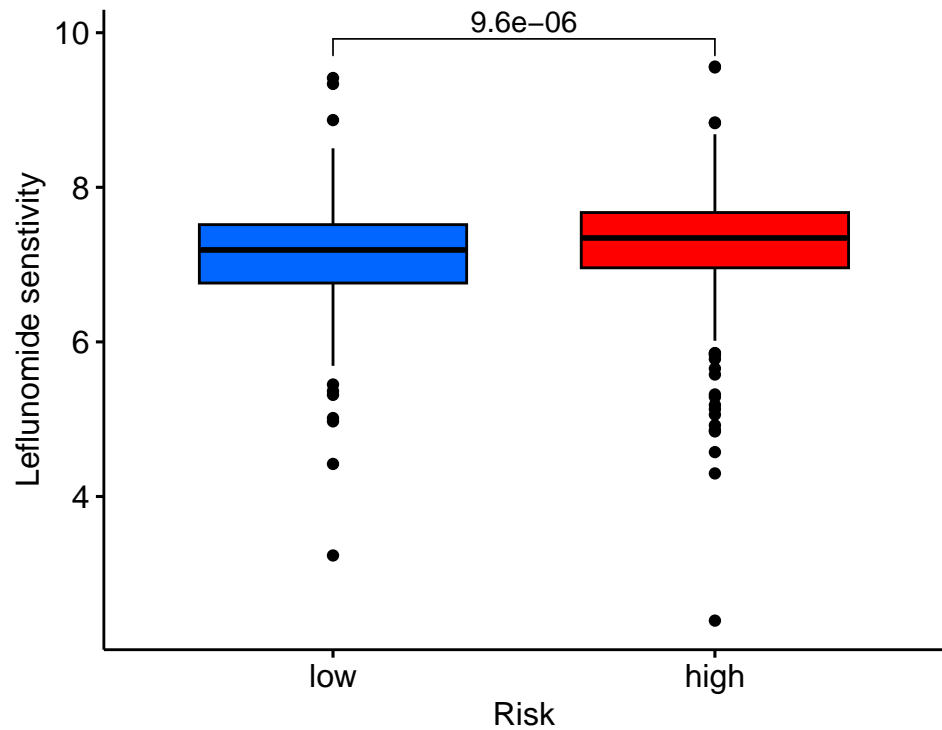

Supplement: Supporting Information 3 — Distinct patterns of drug sensitivity between low-risk and high-risk groups. [file 3423698.f3.zip › Supplementary Material 3/drugSenstivity.Leflunomide.pdf]

Risk 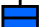 low 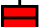 high

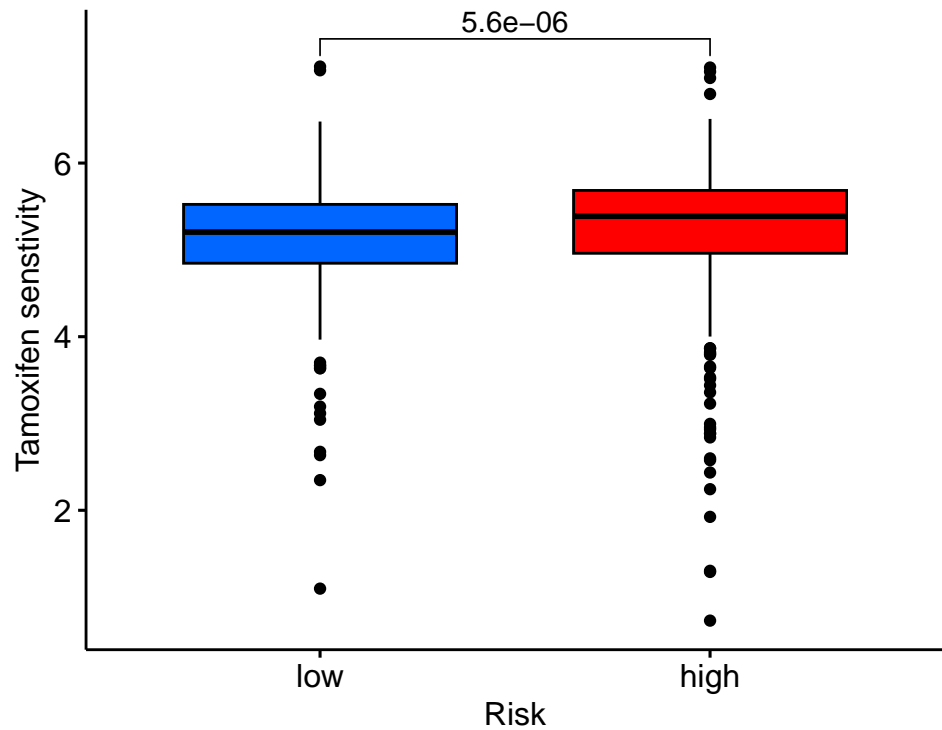

Supplement: Supporting Information 3 — Distinct patterns of drug sensitivity between low-risk and high-risk groups. [file 3423698.f3.zip › Supplementary Material 3/drugSenstivity.Tamoxifen.pdf]

Risk 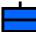 low 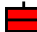 high

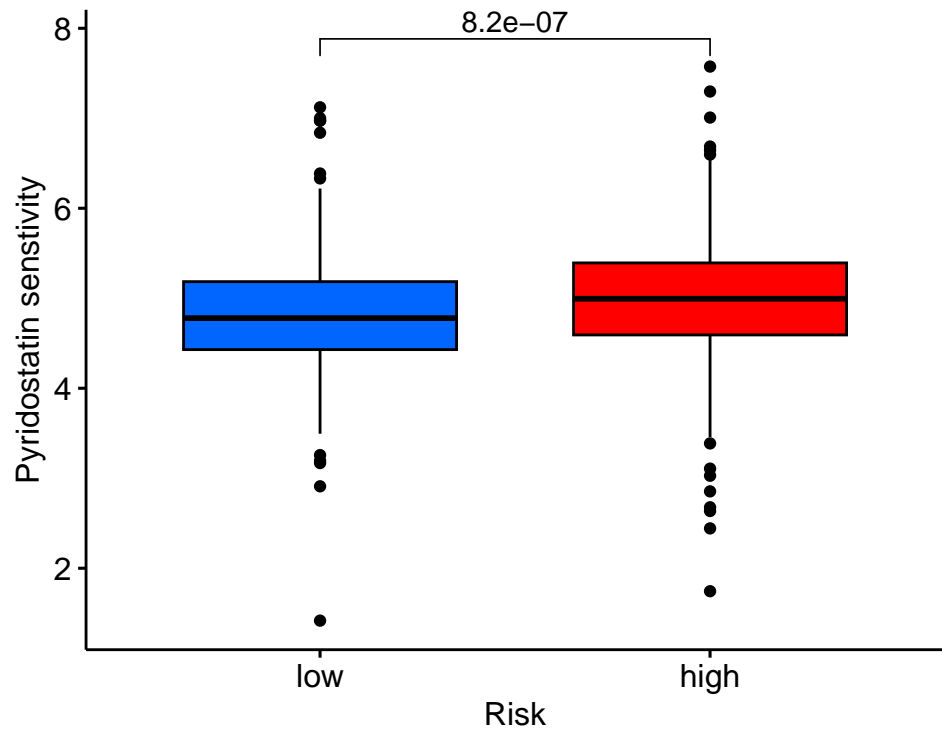

Supplement: Supporting Information 3 — Distinct patterns of drug sensitivity between low-risk and high-risk groups. [file 3423698.f3.zip › Supplementary Material 3/drugSenstivity.Pyridostatin.pdf]

Risk 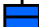 low 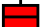 high

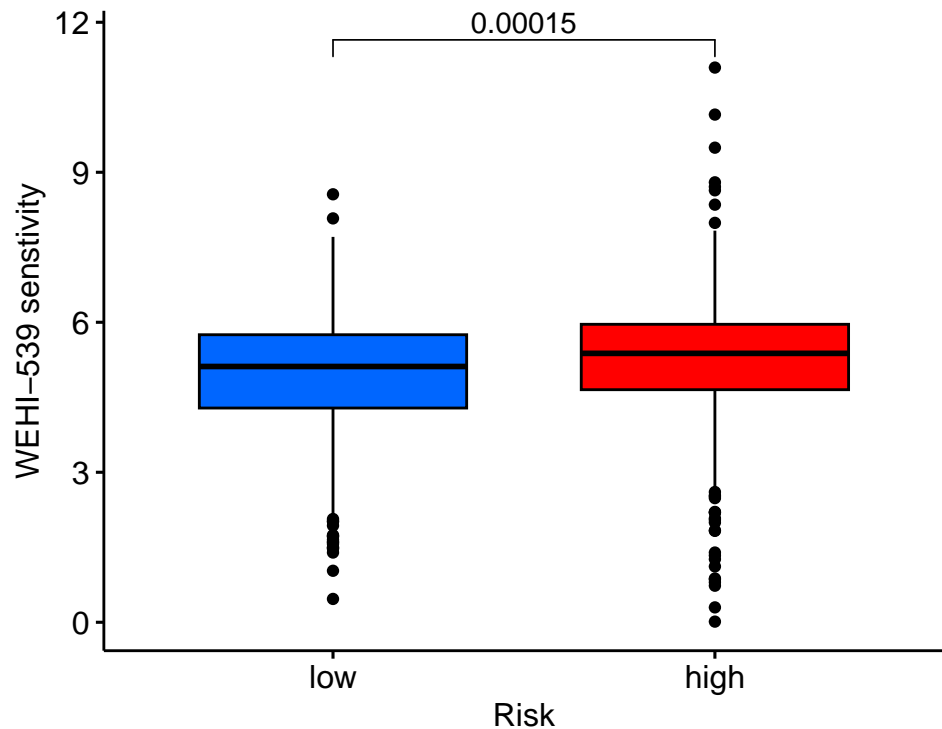

Supplement: Supporting Information 3 — Distinct patterns of drug sensitivity between low-risk and high-risk groups. [file 3423698.f3.zip › Supplementary Material 3/drugSenstivity.WEHI-539.pdf]

Risk 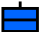 low 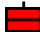 high

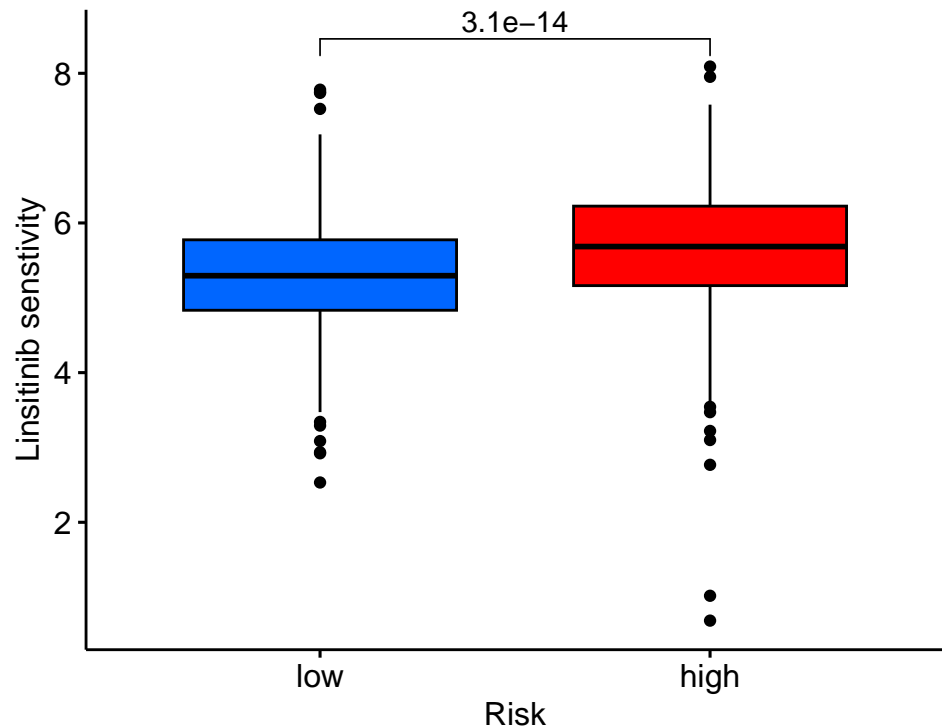

Supplement: Supporting Information 3 — Distinct patterns of drug sensitivity between low-risk and high-risk groups. [file 3423698.f3.zip › Supplementary Material 3/drugSenstivity.Linsitinib.pdf]

Risk low high

$1.9\text{e-}14$

Dactinomycin sensitivity

2.0  
1.5  
1.0  
0.5  
0.0

low

high

Risk

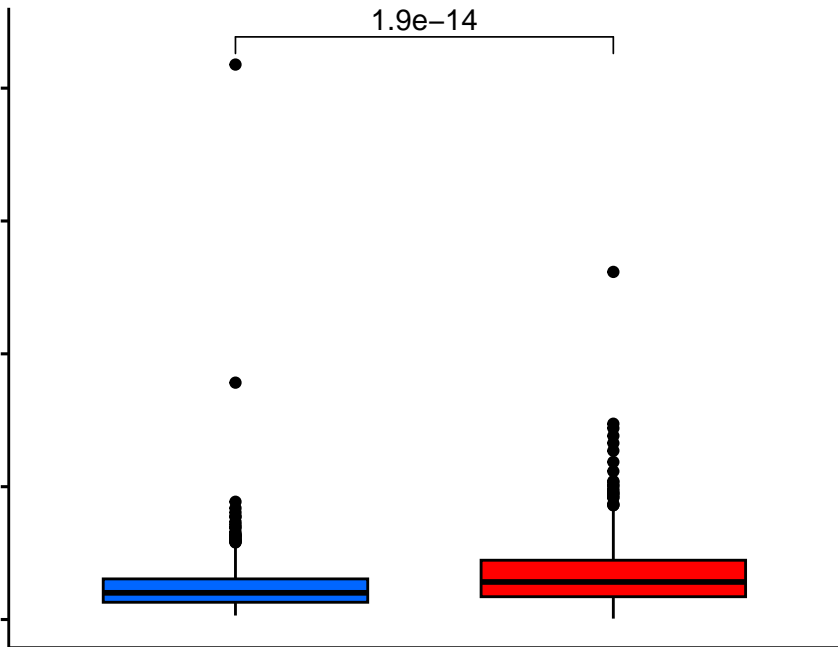

Supplement: Supporting Information 3 — Distinct patterns of drug sensitivity between low-risk and high-risk groups. [file 3423698.f3.zip › Supplementary Material 3/drugSenstivity.Dactinomycin.pdf]

Risk 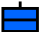 low 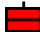 high

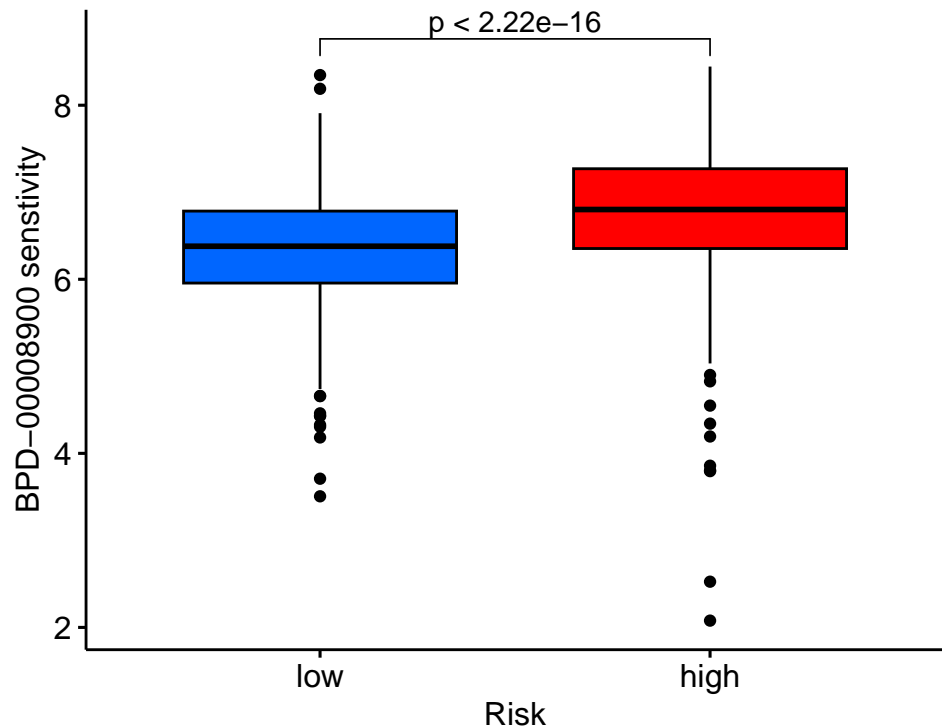

Supplement: Supporting Information 3 — Distinct patterns of drug sensitivity between low-risk and high-risk groups. [file 3423698.f3.zip › Supplementary Material 3/drugSenstivity.BPD-00008900.pdf]

Risk 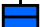 low 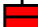 high

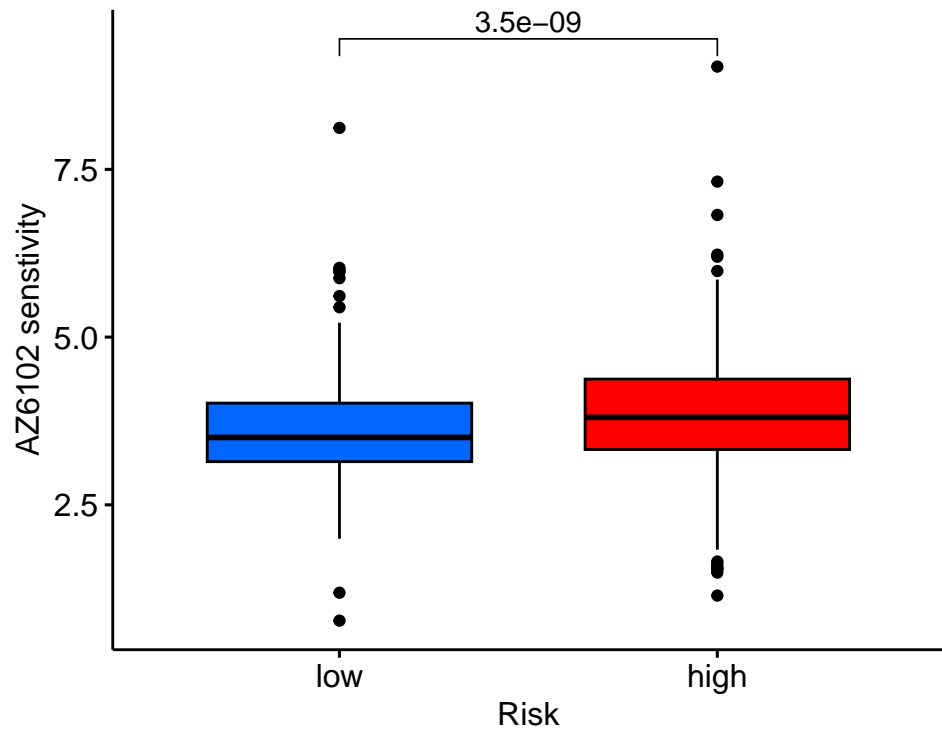

Supplement: Supporting Information 3 — Distinct patterns of drug sensitivity between low-risk and high-risk groups. [file 3423698.f3.zip › Supplementary Material 3/drugSenstivity.AZ6102.pdf]

AZD1208 sensitivity

Risk 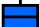 low 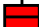 high

$2.4\text{e-}06$

low

high

Risk

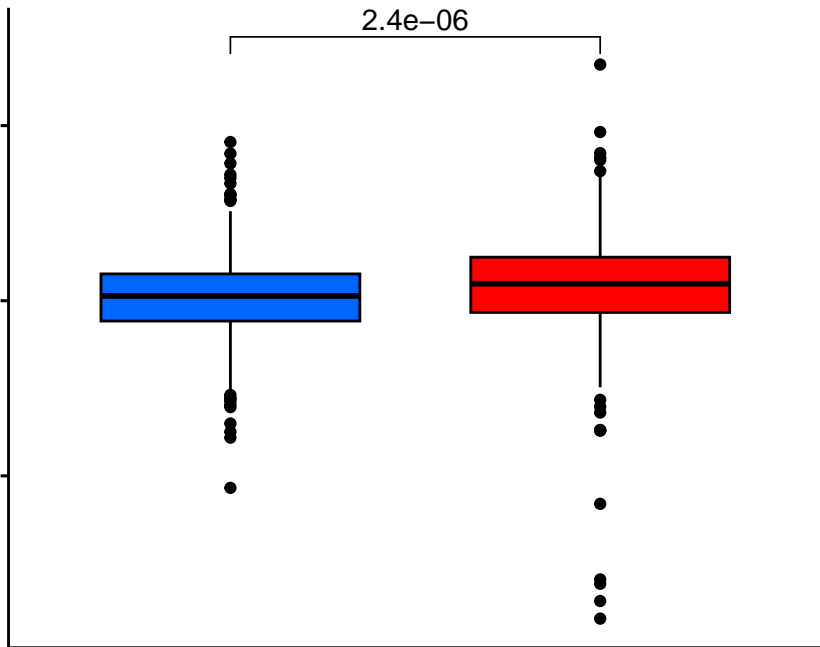

Supplement: Supporting Information 3 — Distinct patterns of drug sensitivity between low-risk and high-risk groups. [file 3423698.f3.zip › Supplementary Material 3/drugSenstivity.AZD1208.pdf]

Risk 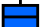 low 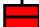 high

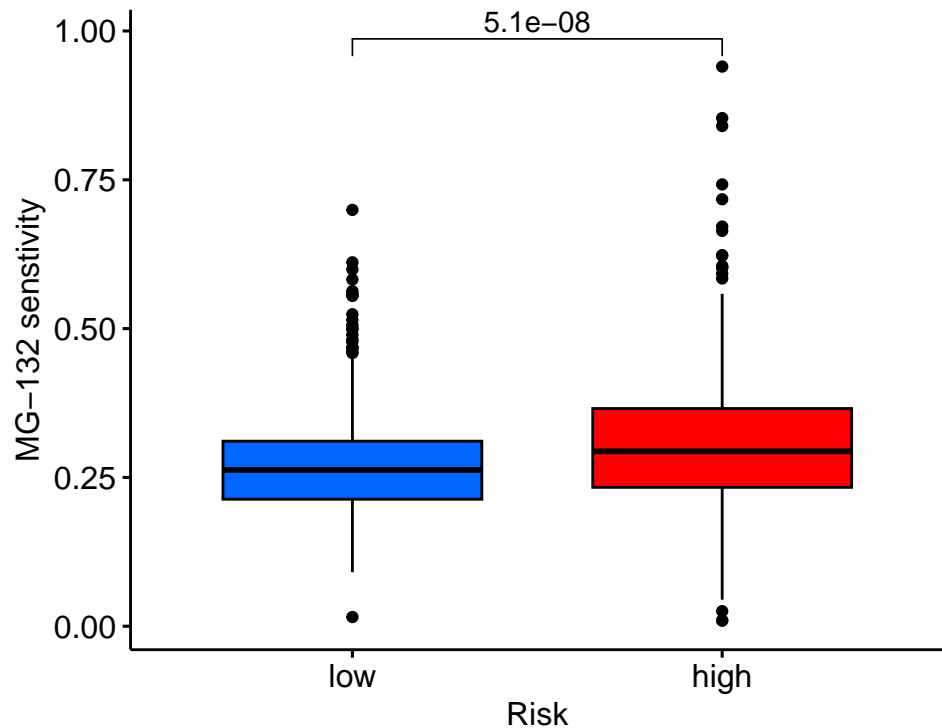

Supplement: Supporting Information 3 — Distinct patterns of drug sensitivity between low-risk and high-risk groups. [file 3423698.f3.zip › Supplementary Material 3/drugSenstivity.MG-132.pdf]

Risk 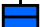 low 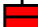 high

$p < 2.22\text{e-}16$

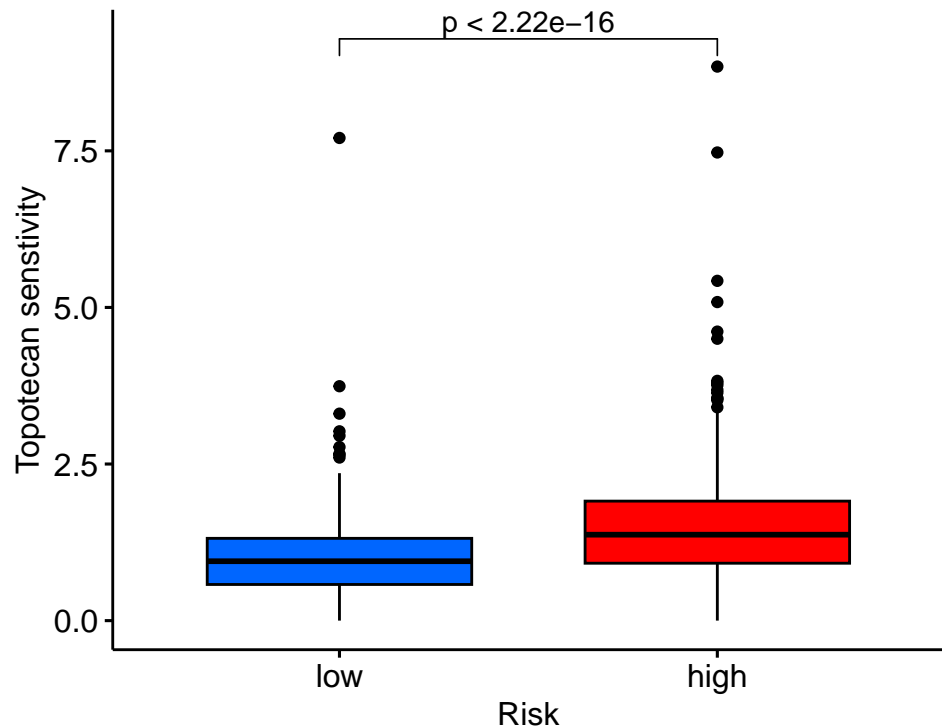

Supplement: Supporting Information 3 — Distinct patterns of drug sensitivity between low-risk and high-risk groups. [file 3423698.f3.zip › Supplementary Material 3/drugSenstivity.Topotecan.pdf]

Risk 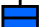 low 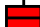 high

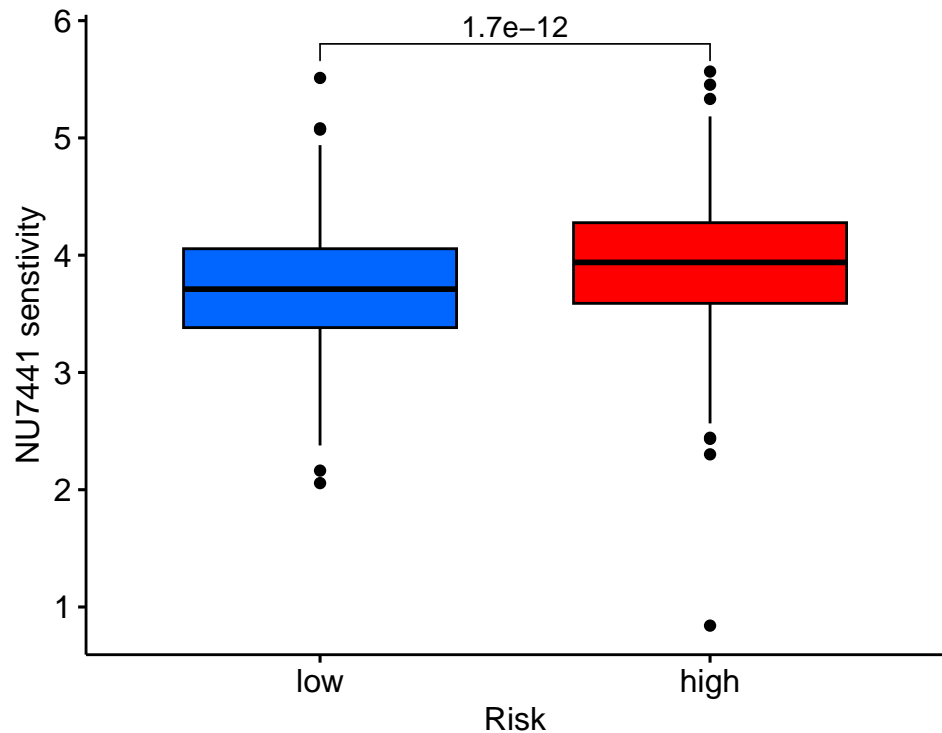

Supplement: Supporting Information 3 — Distinct patterns of drug sensitivity between low-risk and high-risk groups. [file 3423698.f3.zip › Supplementary Material 3/drugSenstivity.NU7441.pdf]

Risk 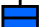 low 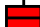 high

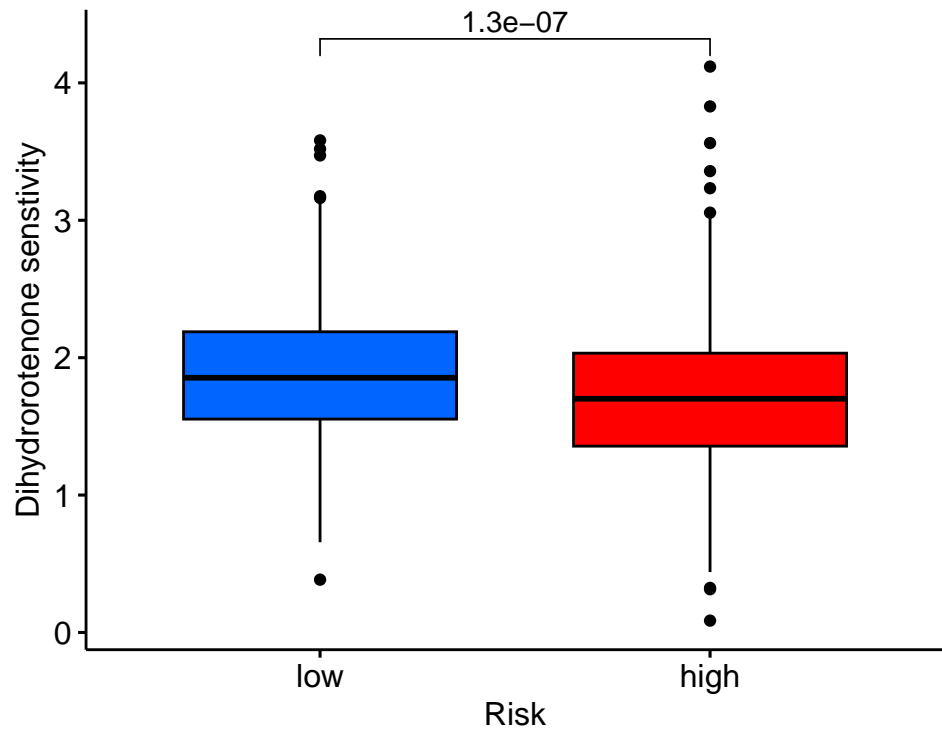

Supplement: Supporting Information 3 — Distinct patterns of drug sensitivity between low-risk and high-risk groups. [file 3423698.f3.zip › Supplementary Material 3/drugSenstivity.Dihydrorotenone.pdf]

Risk 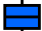 low 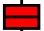 high

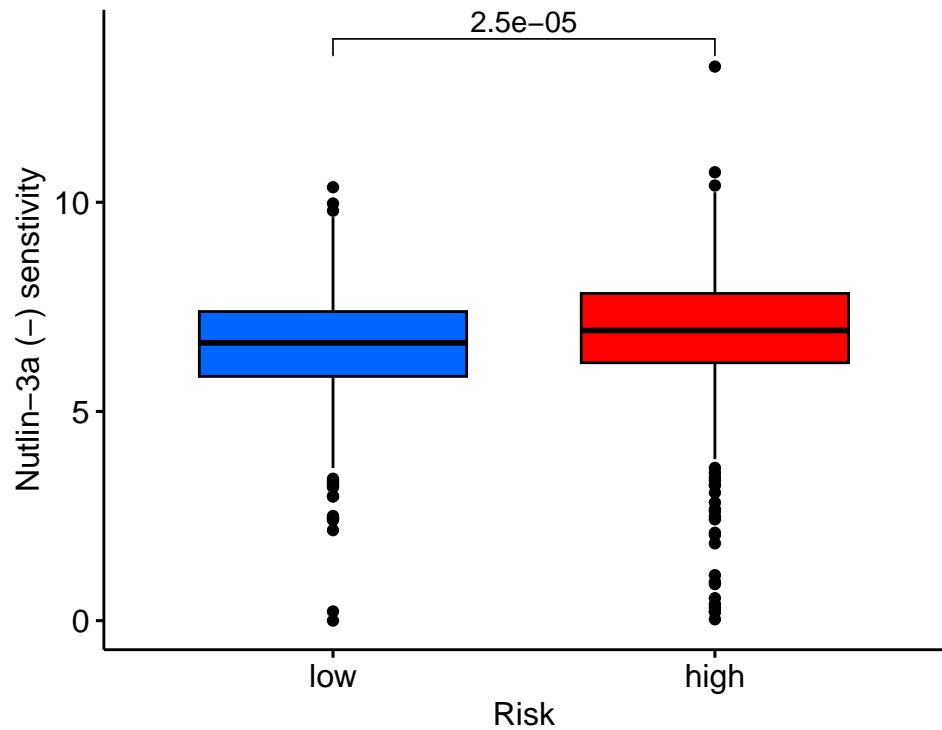

Supplement: Supporting Information 3 — Distinct patterns of drug sensitivity between low-risk and high-risk groups. [file 3423698.f3.zip › Supplementary Material 3/drugSenstivity.Nutlin-3a (-).pdf]

Risk 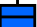 low 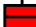 high

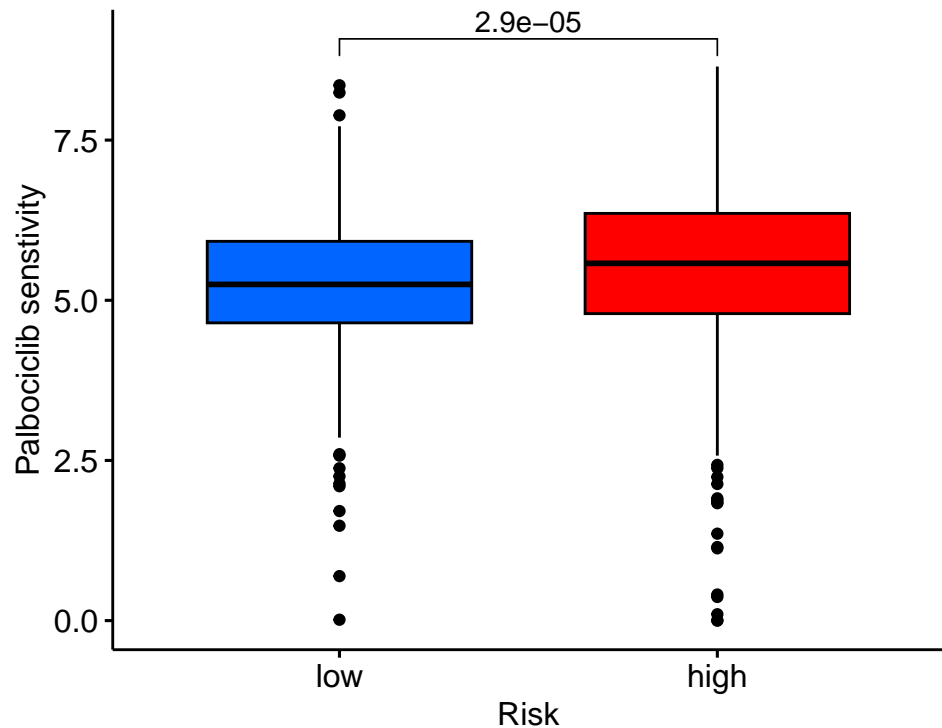

Supplement: Supporting Information 3 — Distinct patterns of drug sensitivity between low-risk and high-risk groups. [file 3423698.f3.zip › Supplementary Material 3/drugSenstivity.Palbociclib.pdf]

Risk 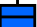 low 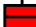 high

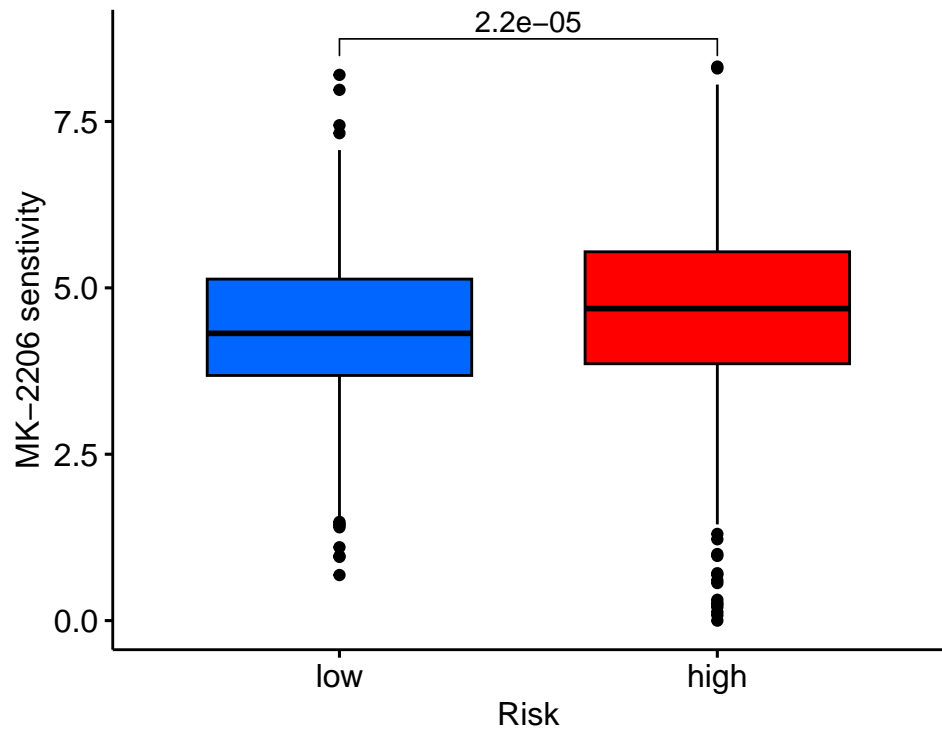

Supplement: Supporting Information 3 — Distinct patterns of drug sensitivity between low-risk and high-risk groups. [file 3423698.f3.zip › Supplementary Material 3/drugSenstivity.MK-2206.pdf]

Risk 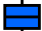 low 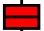 high

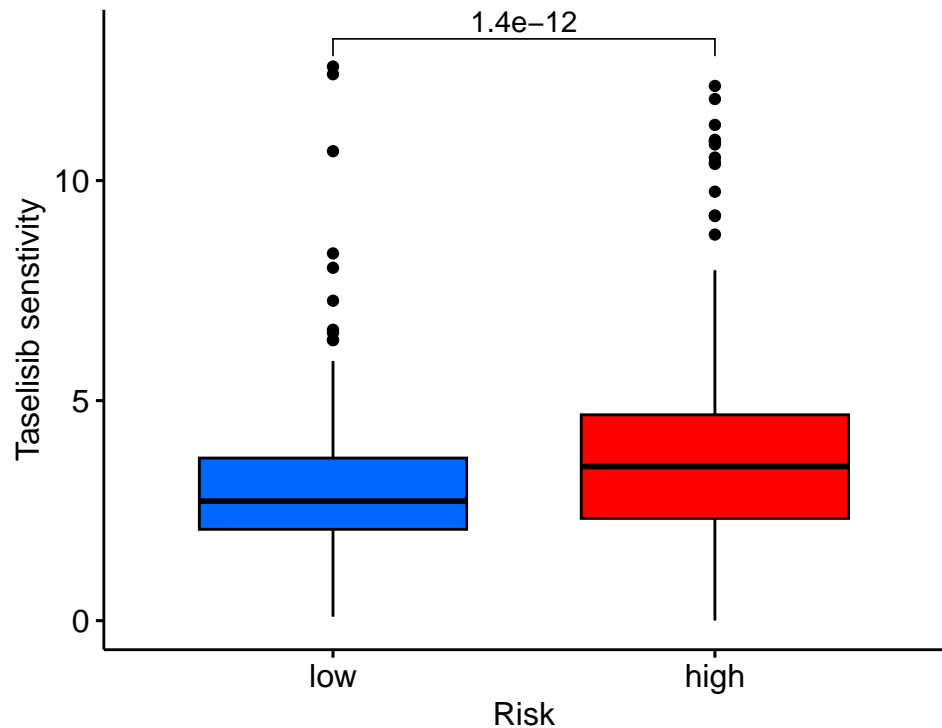

Supplement: Supporting Information 3 — Distinct patterns of drug sensitivity between low-risk and high-risk groups. [file 3423698.f3.zip › Supplementary Material 3/drugSenstivity.Taselisib.pdf]

Risk 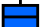 low 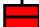 high

$p < 2.22\text{e-}16$

AZ960 sensitivity

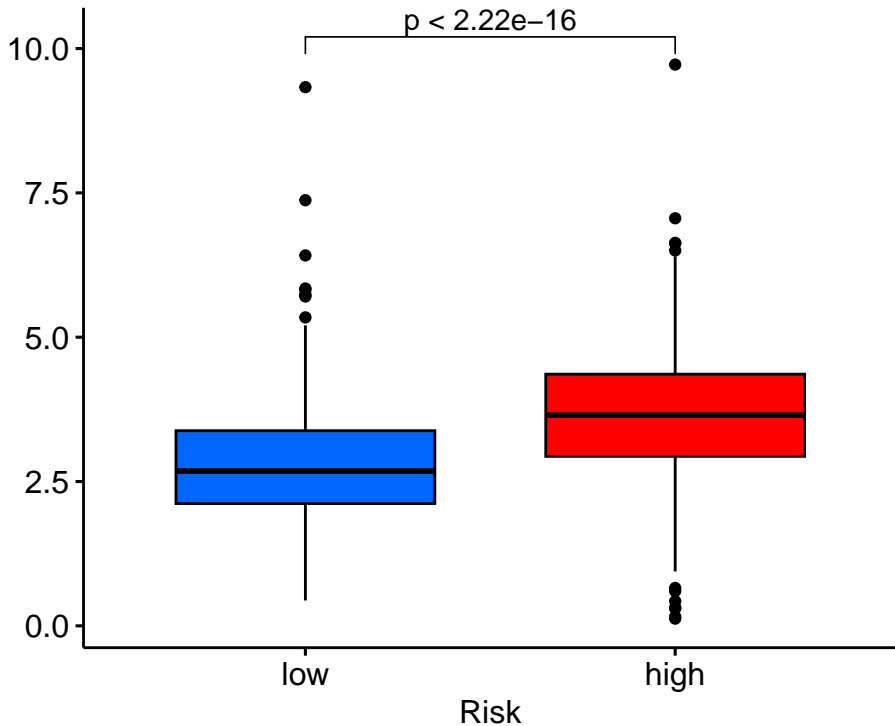

Supplement: Supporting Information 3 — Distinct patterns of drug sensitivity between low-risk and high-risk groups. [file 3423698.f3.zip › Supplementary Material 3/drugSenstivity.AZ960.pdf]

Risk low high

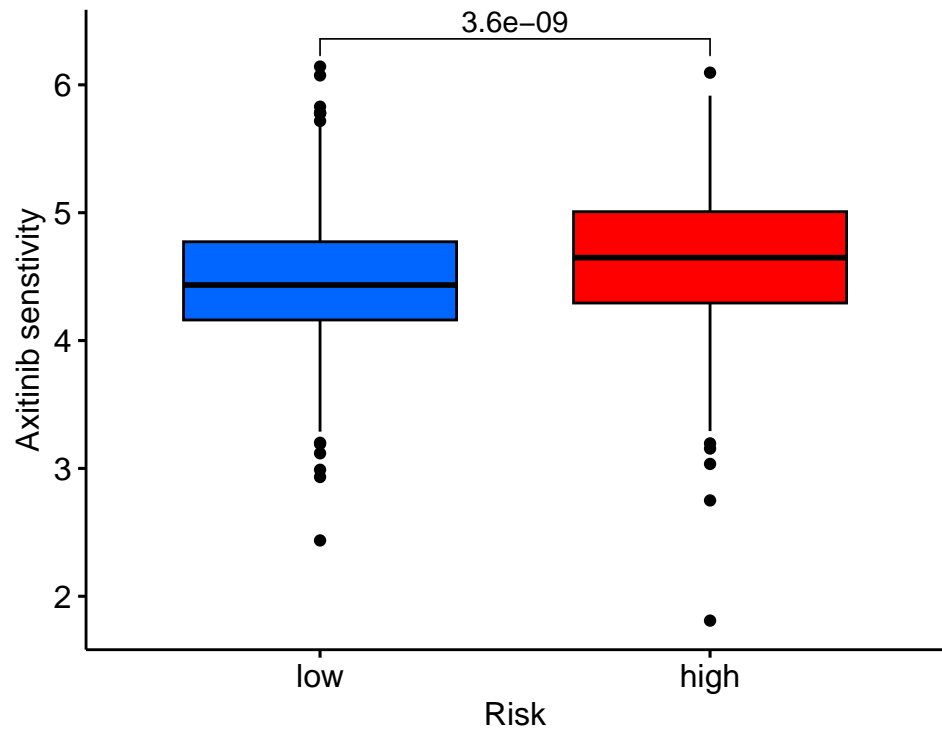

Supplement: Supporting Information 3 — Distinct patterns of drug sensitivity between low-risk and high-risk groups. [file 3423698.f3.zip › Supplementary Material 3/drugSenstivity.Axitinib.pdf]

Risk 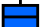 low 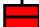 high

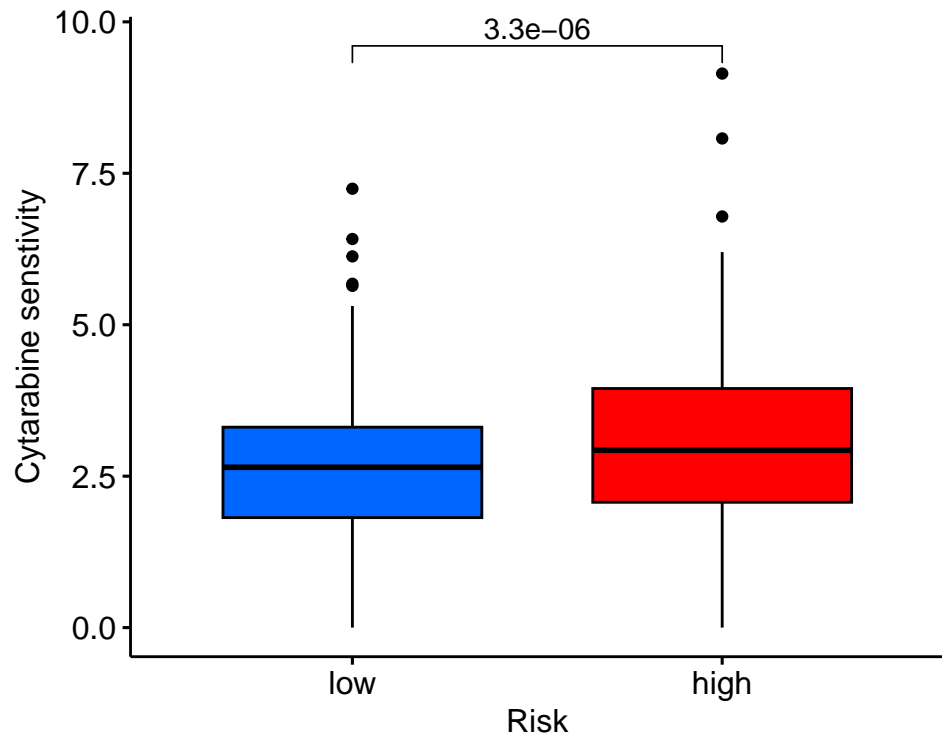

Supplement: Supporting Information 3 — Distinct patterns of drug sensitivity between low-risk and high-risk groups. [file 3423698.f3.zip › Supplementary Material 3/drugSenstivity.Cytarabine.pdf]

Risk 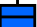 low 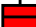 high

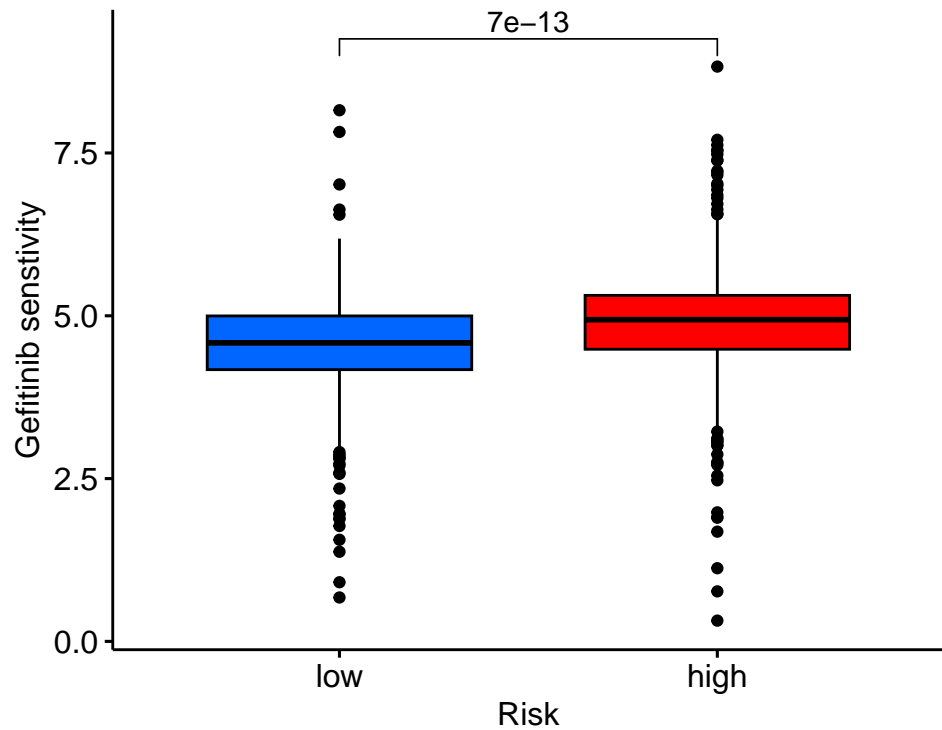

Supplement: Supporting Information 3 — Distinct patterns of drug sensitivity between low-risk and high-risk groups. [file 3423698.f3.zip › Supplementary Material 3/drugSenstivity.Gefitinib.pdf]

Risk low high

4.1e-06

Paclitaxel sensitivity

4

3

2

1

0

low

high

Risk

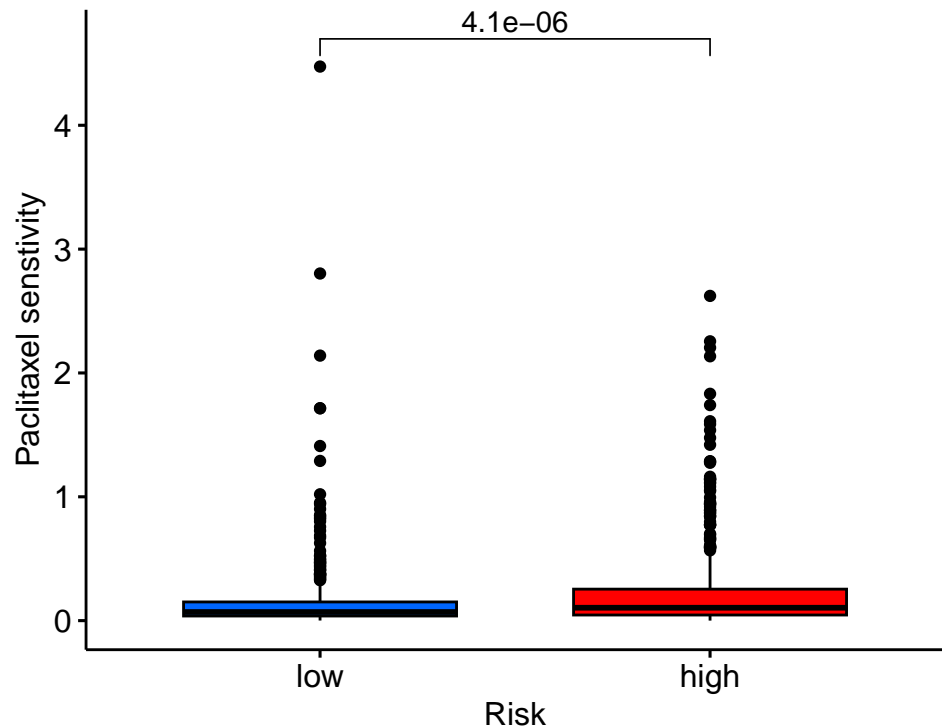

Supplement: Supporting Information 3 — Distinct patterns of drug sensitivity between low-risk and high-risk groups. [file 3423698.f3.zip › Supplementary Material 3/drugSenstivity.Paclitaxel.pdf]

Risk 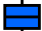 low 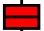 high

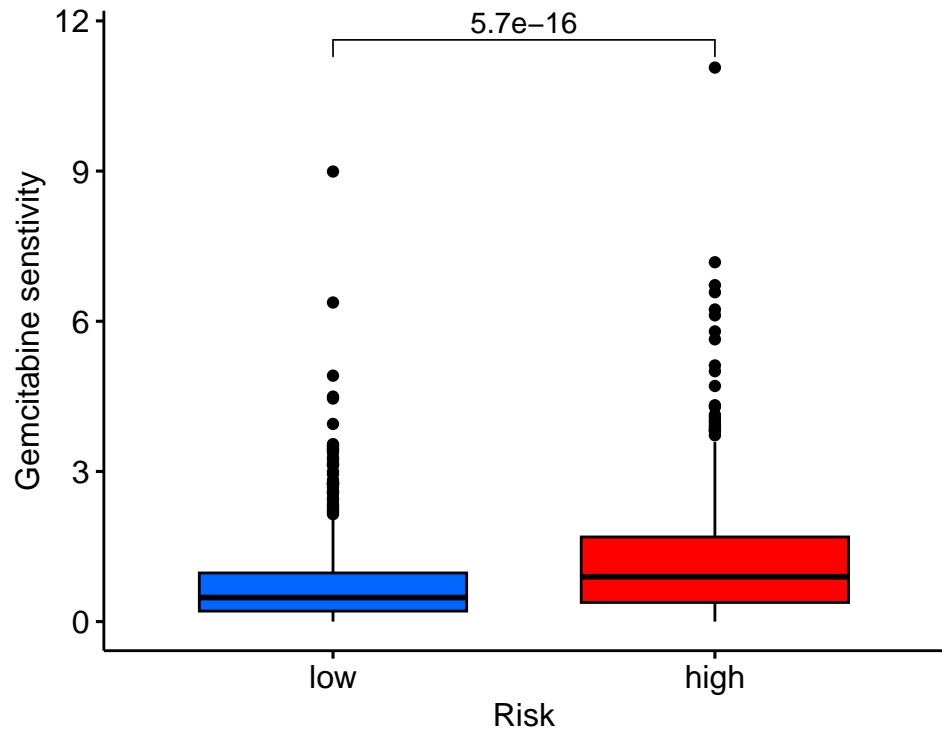

Supplement: Supporting Information 3 — Distinct patterns of drug sensitivity between low-risk and high-risk groups. [file 3423698.f3.zip › Supplementary Material 3/drugSenstivity.Gemcitabine.pdf]

Risk 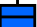 low 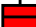 high

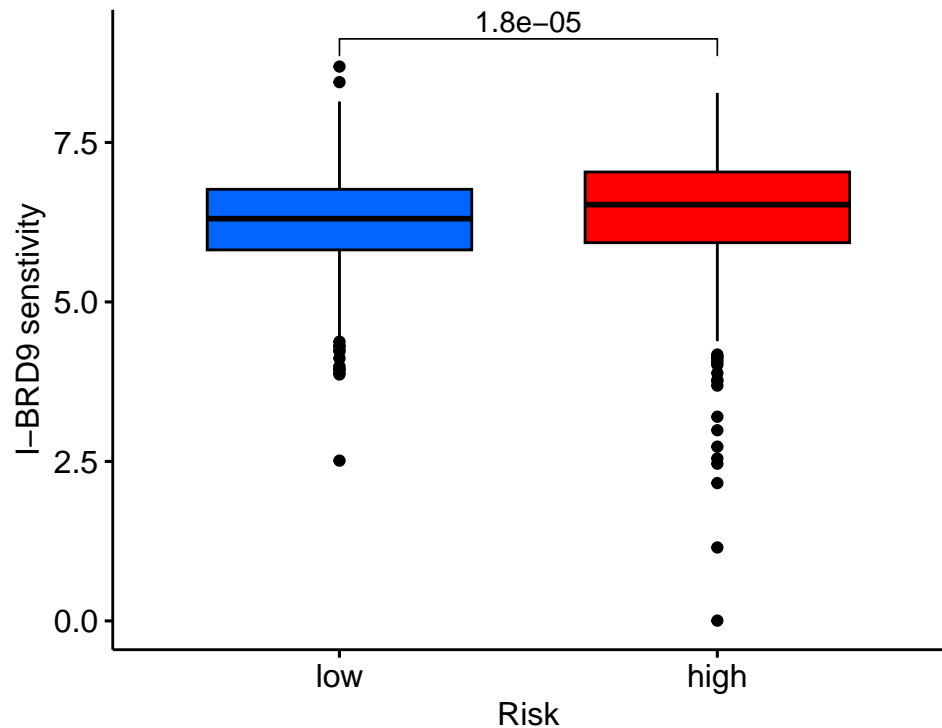

Supplement: Supporting Information 3 — Distinct patterns of drug sensitivity between low-risk and high-risk groups. [file 3423698.f3.zip › Supplementary Material 3/drugSenstivity.I-BRD9.pdf]

Risk low high

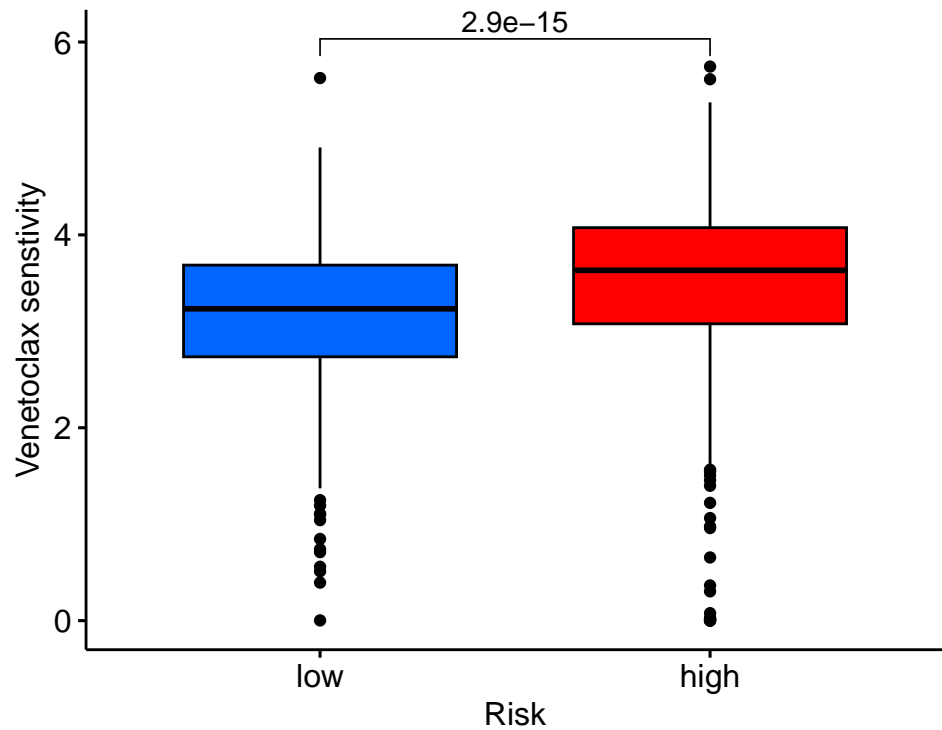

Supplement: Supporting Information 3 — Distinct patterns of drug sensitivity between low-risk and high-risk groups. [file 3423698.f3.zip › Supplementary Material 3/drugSenstivity.Venetoclax.pdf]

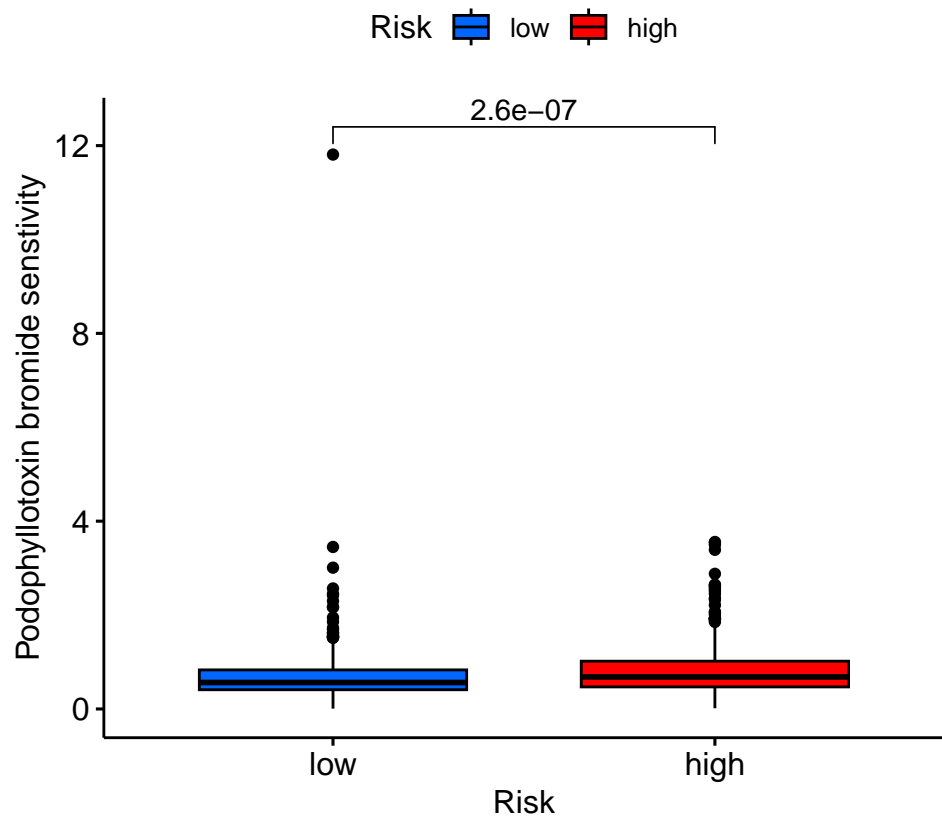

Supplement: Supporting Information 3 — Distinct patterns of drug sensitivity between low-risk and high-risk groups. [file 3423698.f3.zip › Supplementary Material 3/drugSenstivity.Podophyllotoxin bromide.pdf]

Risk 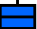 low 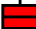 high

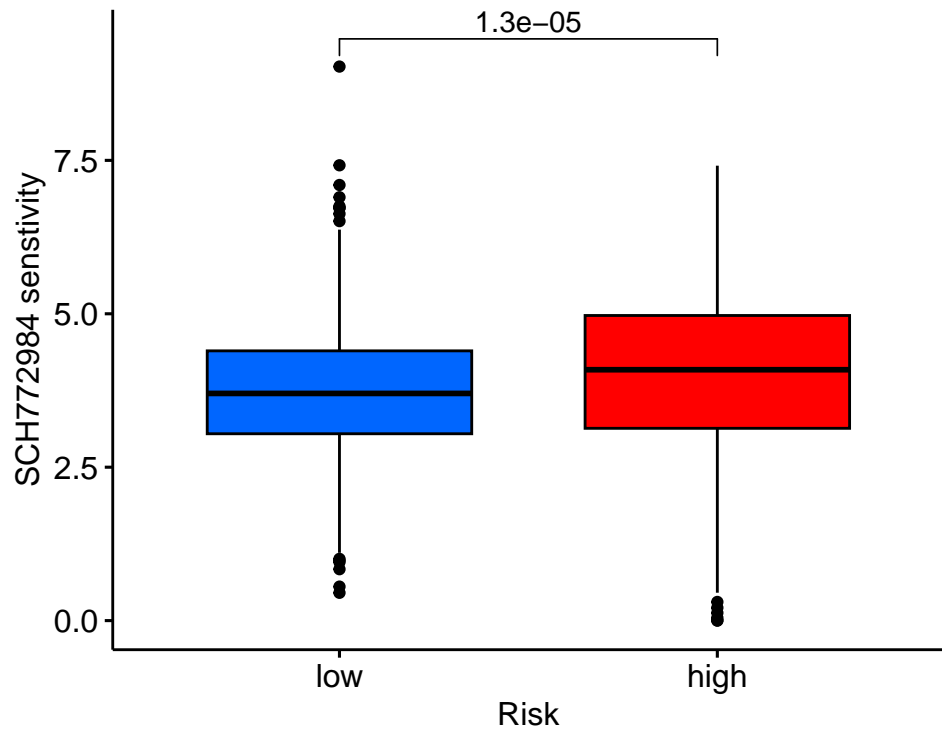

Supplement: Supporting Information 3 — Distinct patterns of drug sensitivity between low-risk and high-risk groups. [file 3423698.f3.zip › Supplementary Material 3/drugSenstivity.SCH772984.pdf]

Risk 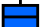 low 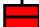 high

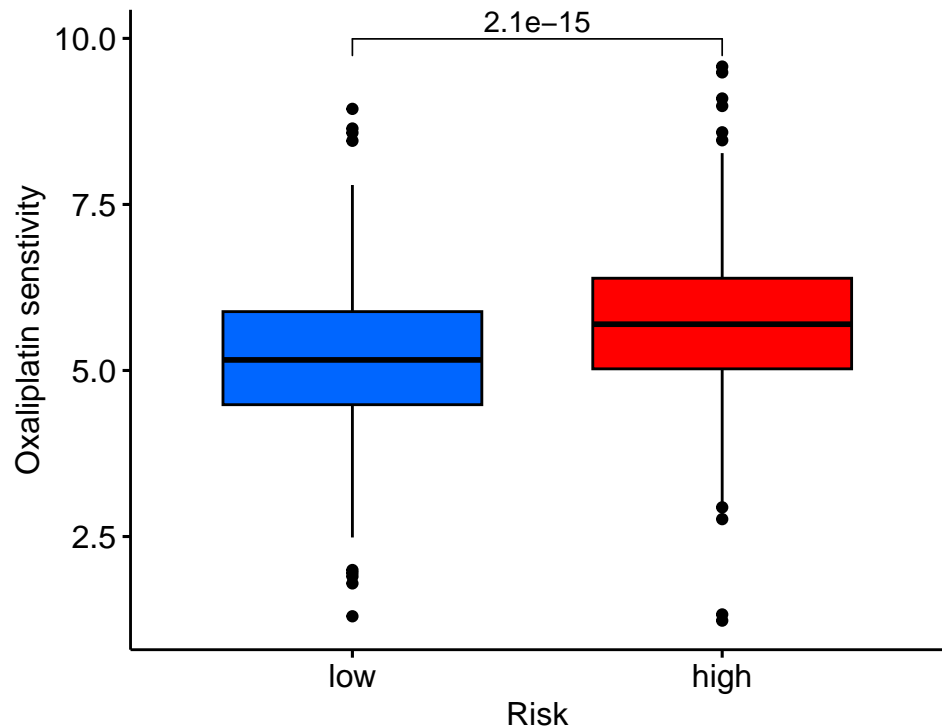

Supplement: Supporting Information 3 — Distinct patterns of drug sensitivity between low-risk and high-risk groups. [file 3423698.f3.zip › Supplementary Material 3/drugSenstivity.Oxaliplatin.pdf]

Risk low high

$p < 2.22e-16$

Temozolomide sensitivity

10

5

0

low

high

Risk

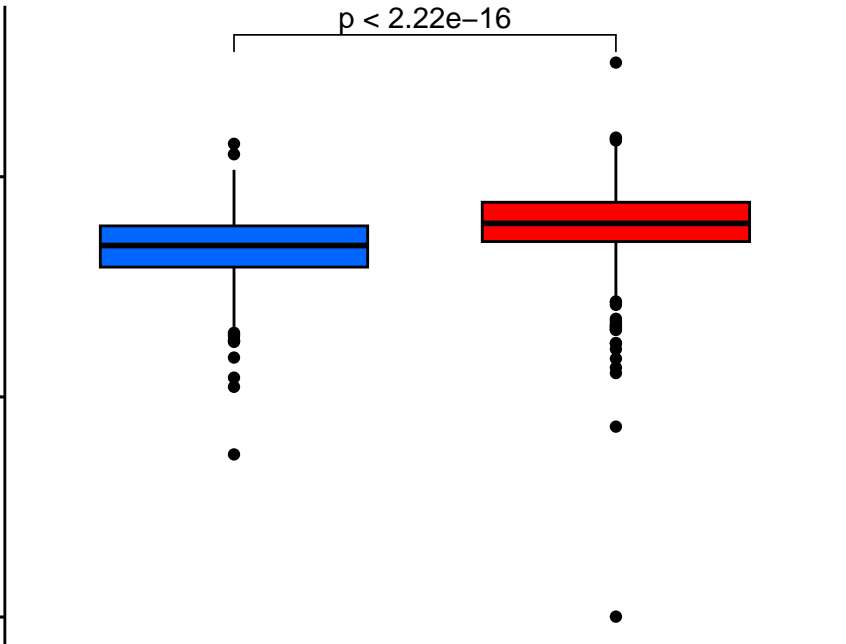

Supplement: Supporting Information 3 — Distinct patterns of drug sensitivity between low-risk and high-risk groups. [file 3423698.f3.zip › Supplementary Material 3/drugSenstivity.Temozolomide.pdf]

Risk low high

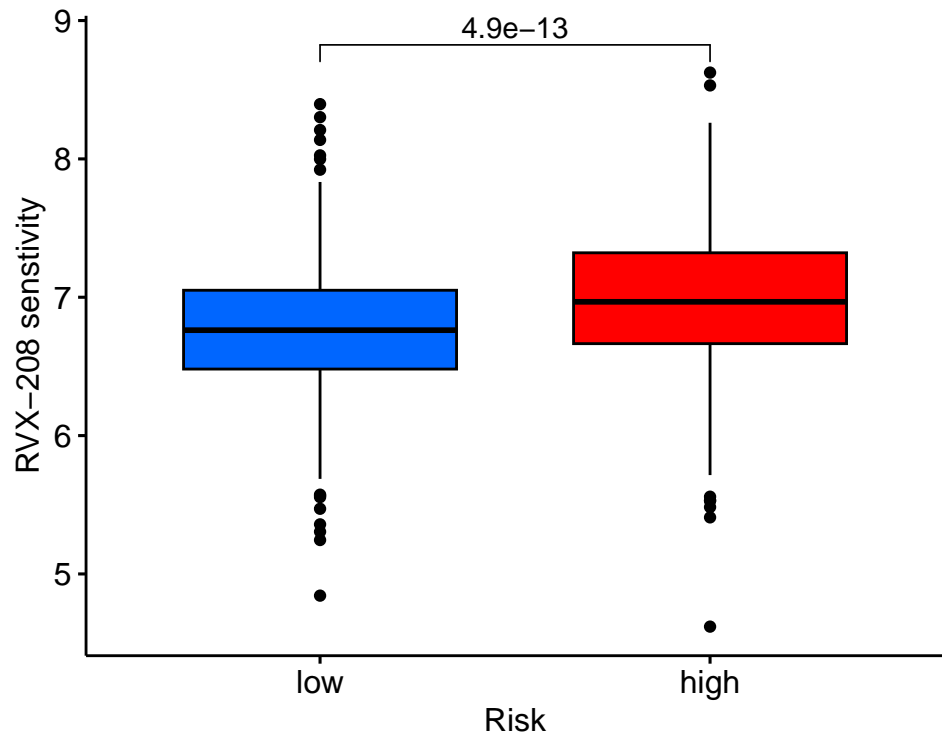

Supplement: Supporting Information 3 — Distinct patterns of drug sensitivity between low-risk and high-risk groups. [file 3423698.f3.zip › Supplementary Material 3/drugSenstivity.RVX-208.pdf]

Risk 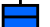 low 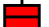 high

$p < 2.22\text{e-}16$

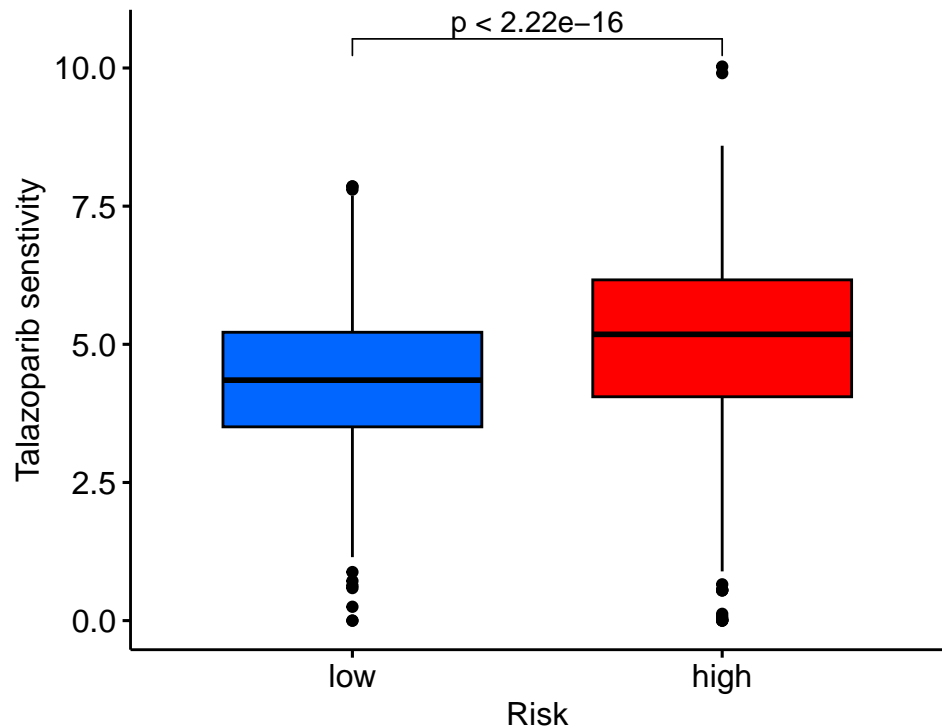

Supplement: Supporting Information 3 — Distinct patterns of drug sensitivity between low-risk and high-risk groups. [file 3423698.f3.zip › Supplementary Material 3/drugSenstivity.Talazoparib.pdf]

Risk 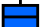 low 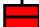 high

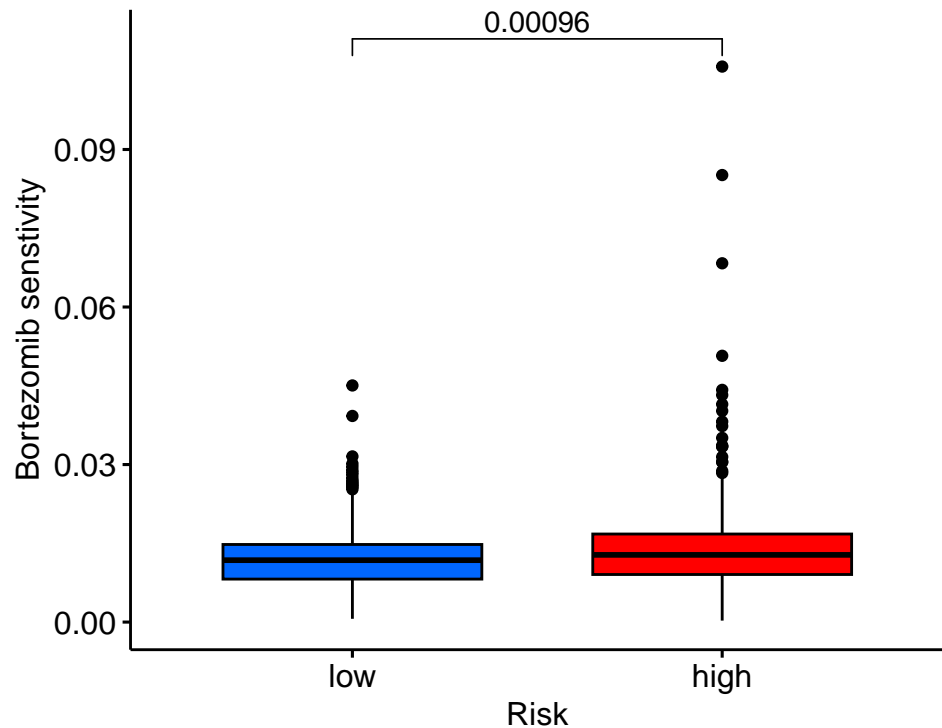

Supplement: Supporting Information 3 — Distinct patterns of drug sensitivity between low-risk and high-risk groups. [file 3423698.f3.zip › Supplementary Material 3/drugSenstivity.Bortezomib.pdf]

Risk 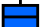 low 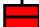 high

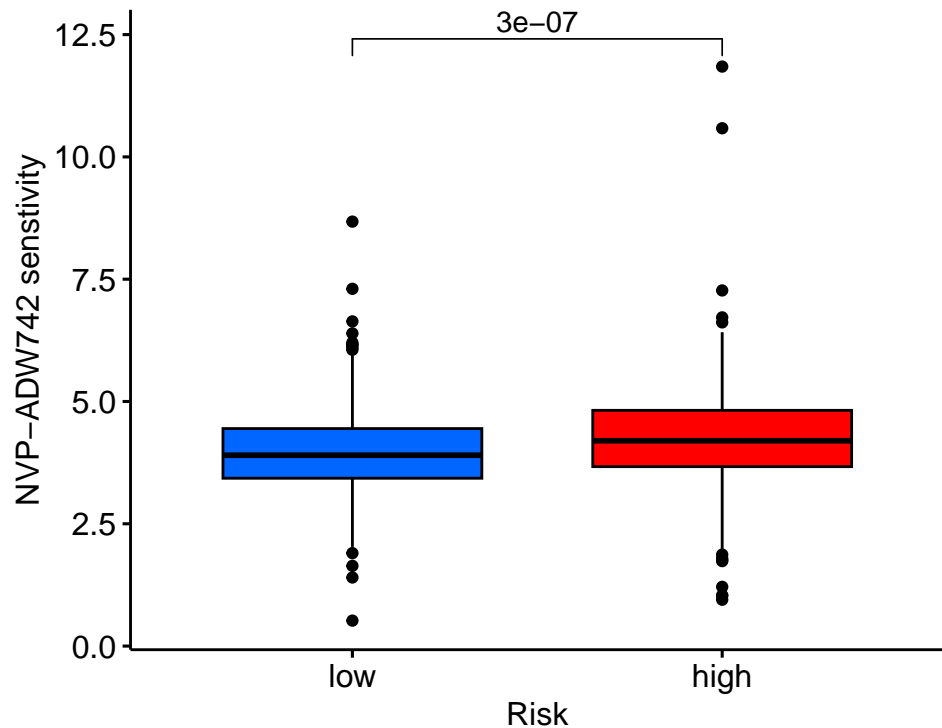

Supplement: Supporting Information 3 — Distinct patterns of drug sensitivity between low-risk and high-risk groups. [file 3423698.f3.zip › Supplementary Material 3/drugSenstivity.NVP-ADW742.pdf]

Risk 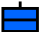 low 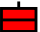 high

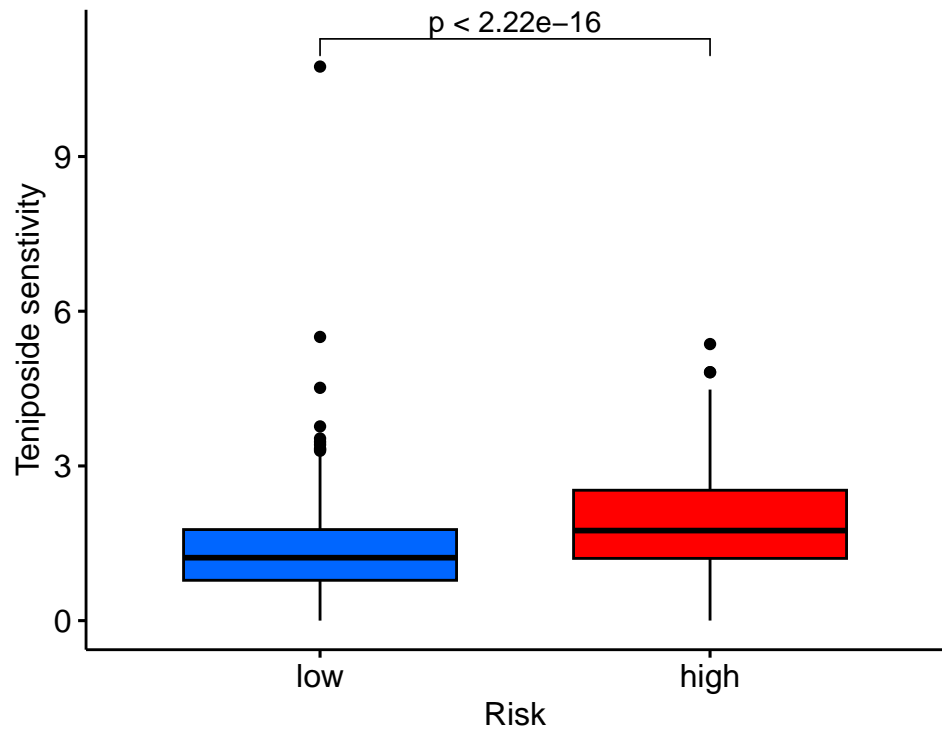

Supplement: Supporting Information 3 — Distinct patterns of drug sensitivity between low-risk and high-risk groups. [file 3423698.f3.zip › Supplementary Material 3/drugSenstivity.Teniposide.pdf]

Risk 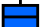 low 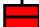 high

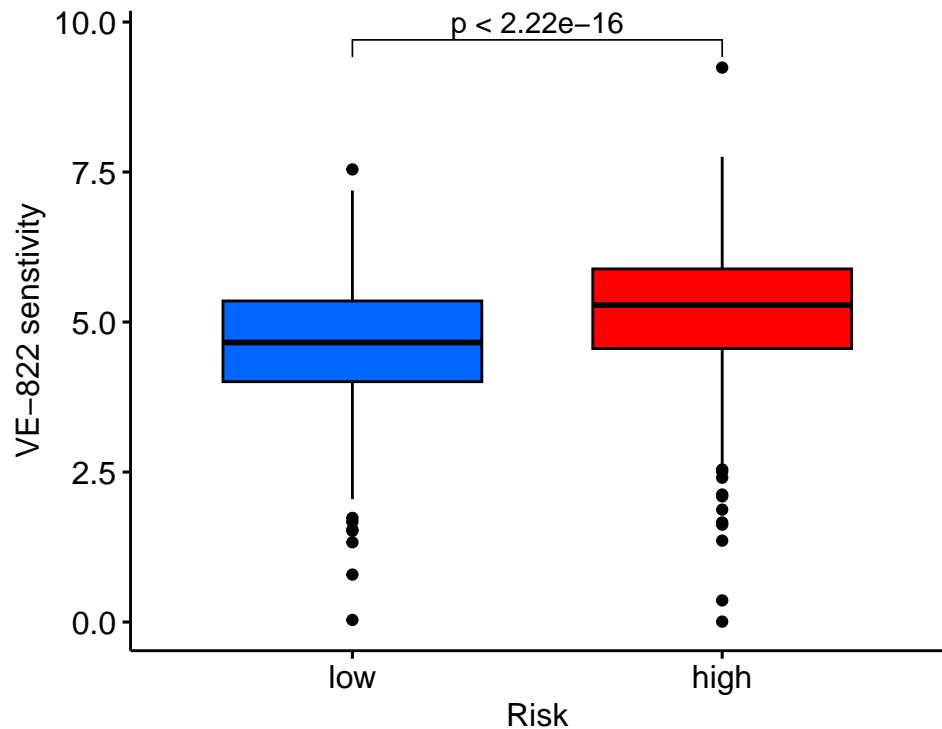

Supplement: Supporting Information 3 — Distinct patterns of drug sensitivity between low-risk and high-risk groups. [file 3423698.f3.zip › Supplementary Material 3/drugSenstivity.VE-822.pdf]

Risk 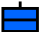 low 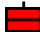 high

$p < 2.22\text{e-}16$

Staurosporine sensitivity

2

1

0

low

high

Risk

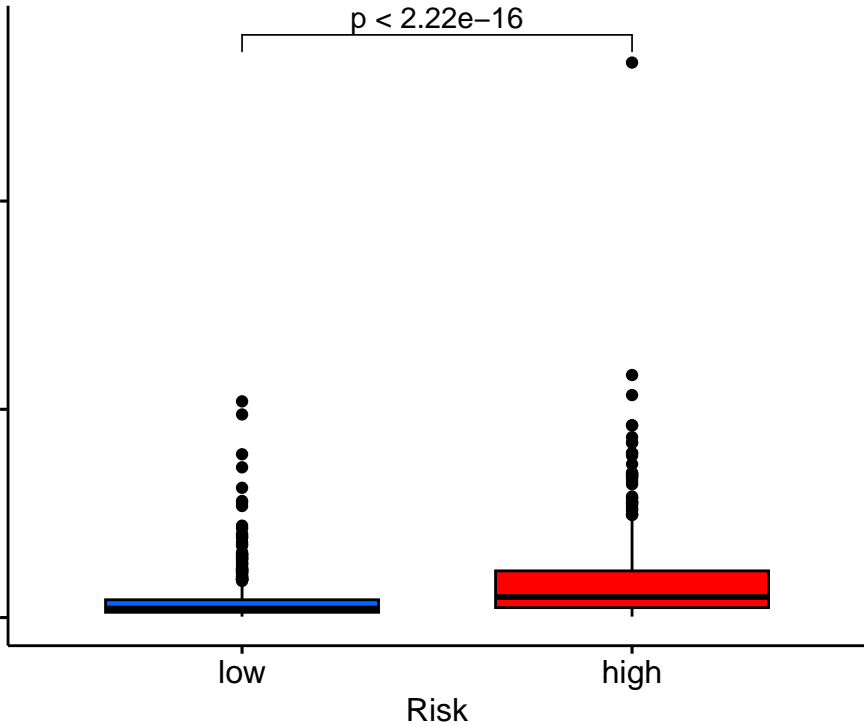

Supplement: Supporting Information 3 — Distinct patterns of drug sensitivity between low-risk and high-risk groups. [file 3423698.f3.zip › Supplementary Material 3/drugSenstivity.Staurosporine.pdf]

Risk 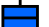 low 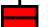 high

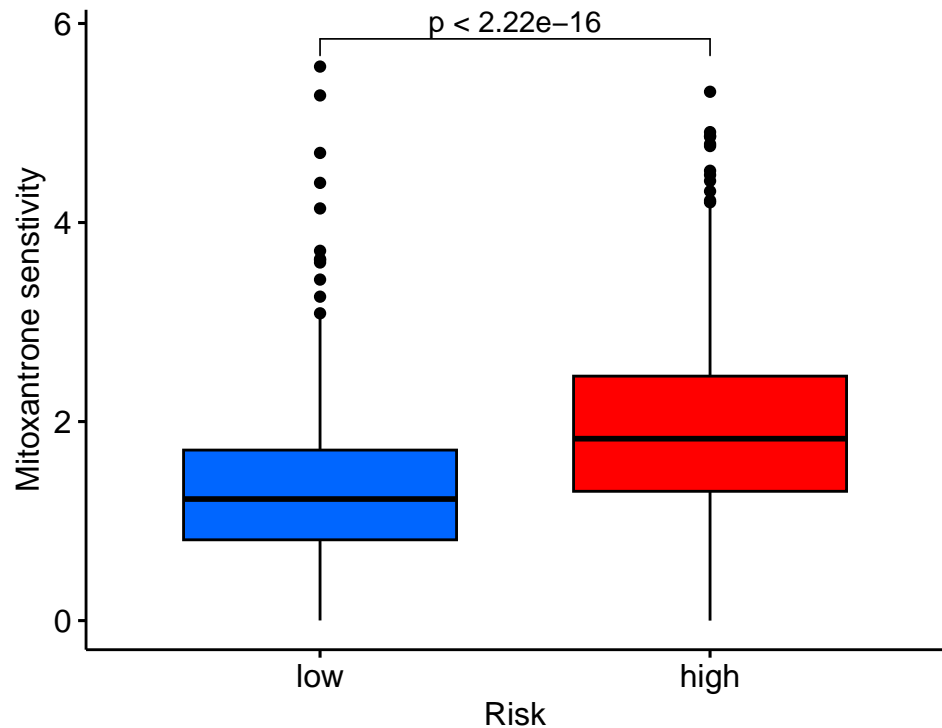

Supplement: Supporting Information 3 — Distinct patterns of drug sensitivity between low-risk and high-risk groups. [file 3423698.f3.zip › Supplementary Material 3/drugSenstivity.Mitoxantrone.pdf]

Risk 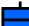 low 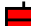 high

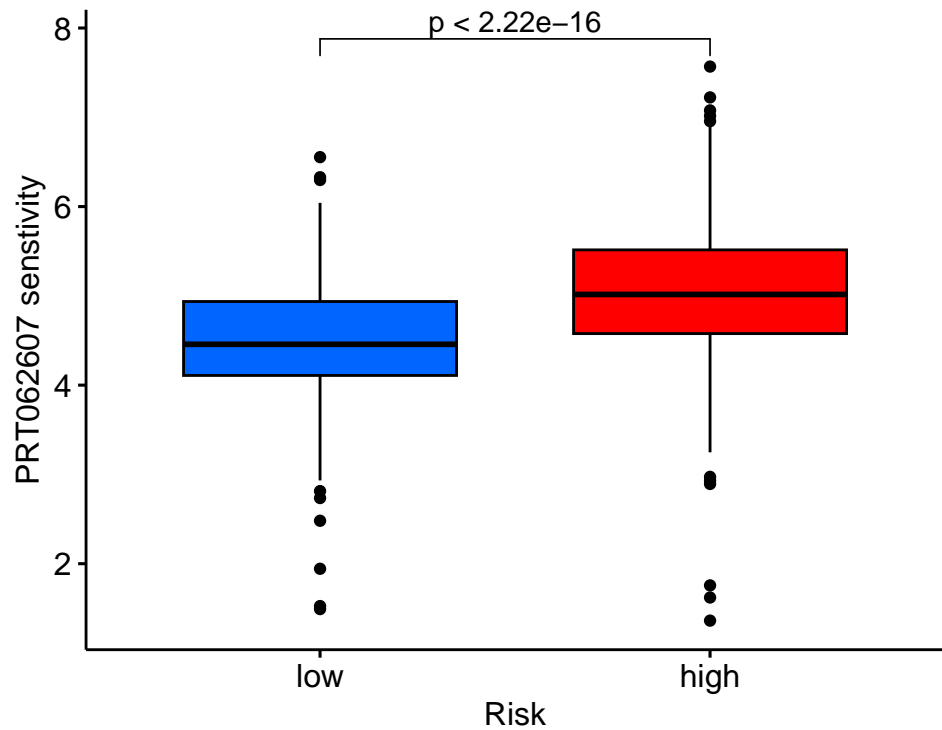

Supplement: Supporting Information 3 — Distinct patterns of drug sensitivity between low-risk and high-risk groups. [file 3423698.f3.zip › Supplementary Material 3/drugSenstivity.PRT062607.pdf]

Risk low high

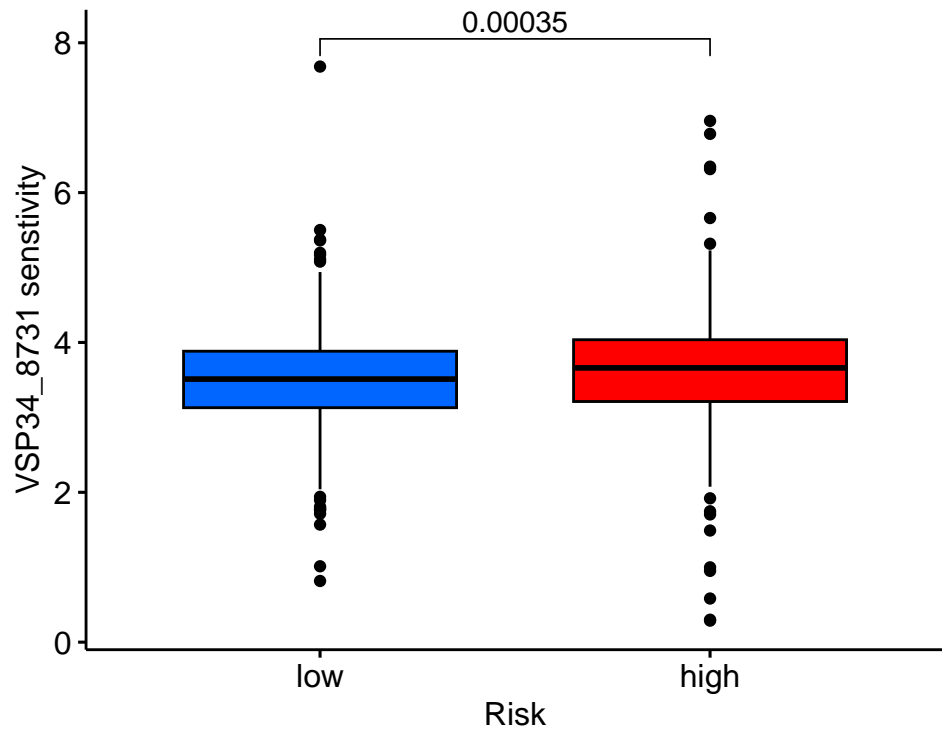

Supplement: Supporting Information 3 — Distinct patterns of drug sensitivity between low-risk and high-risk groups. [file 3423698.f3.zip › Supplementary Material 3/drugSenstivity.VSP34_8731.pdf]

Risk 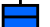 low 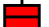 high

$p < 2.22\text{e-}16$

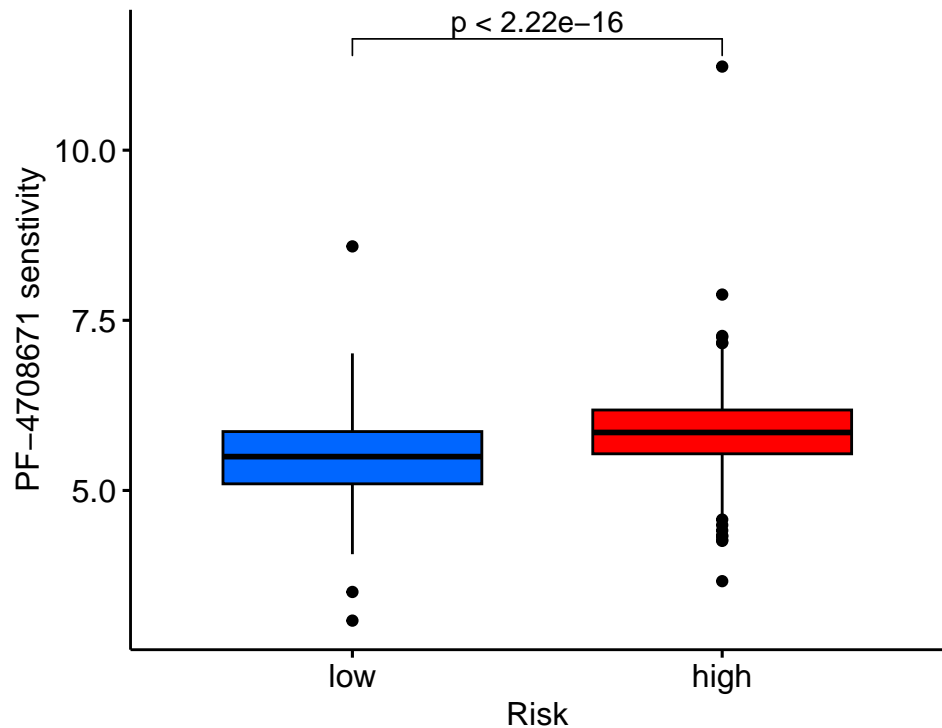

Supplement: Supporting Information 3 — Distinct patterns of drug sensitivity between low-risk and high-risk groups. [file 3423698.f3.zip › Supplementary Material 3/drugSenstivity.PF-4708671.pdf]

Risk 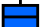 low 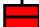 high

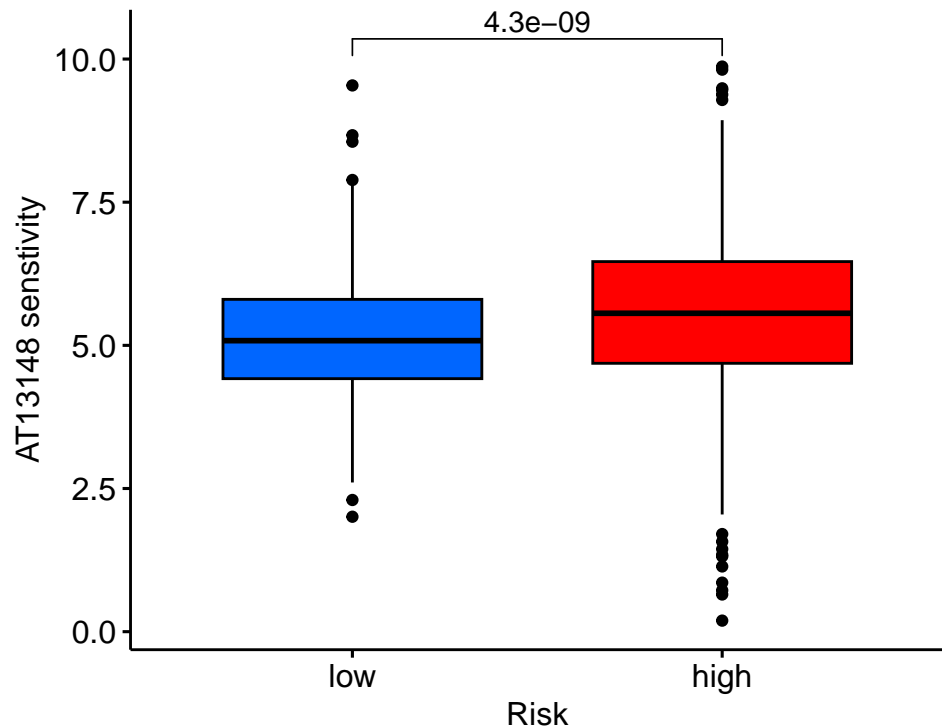

Supplement: Supporting Information 3 — Distinct patterns of drug sensitivity between low-risk and high-risk groups. [file 3423698.f3.zip › Supplementary Material 3/drugSenstivity.AT13148.pdf]

Risk low high

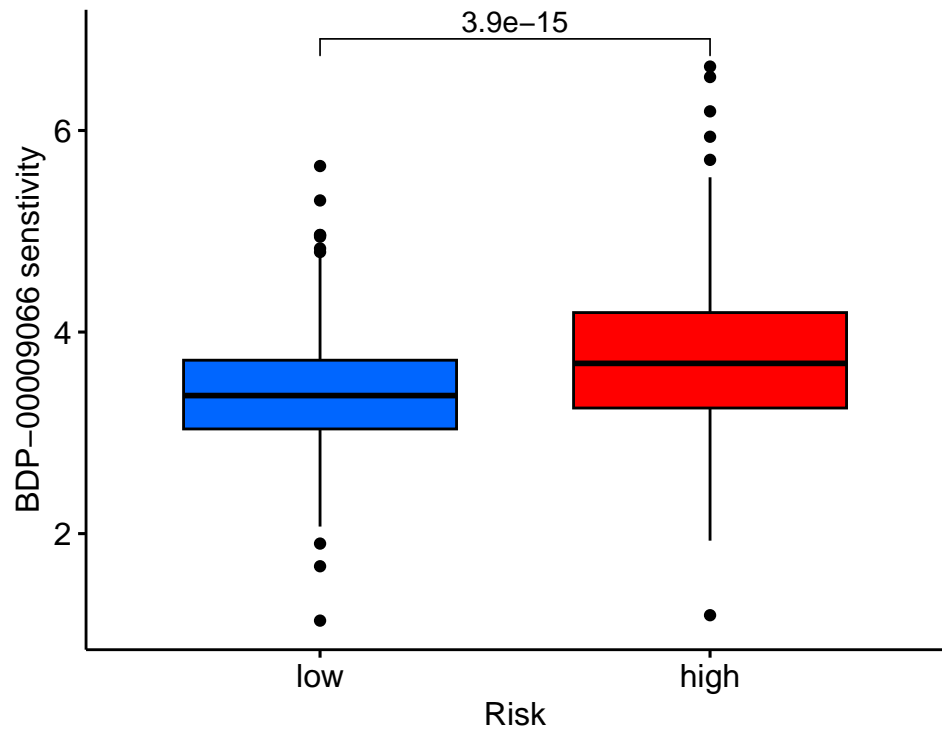

Supplement: Supporting Information 3 — Distinct patterns of drug sensitivity between low-risk and high-risk groups. [file 3423698.f3.zip › Supplementary Material 3/drugSenstivity.BDP-00009066.pdf]

Risk 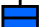 low 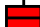 high

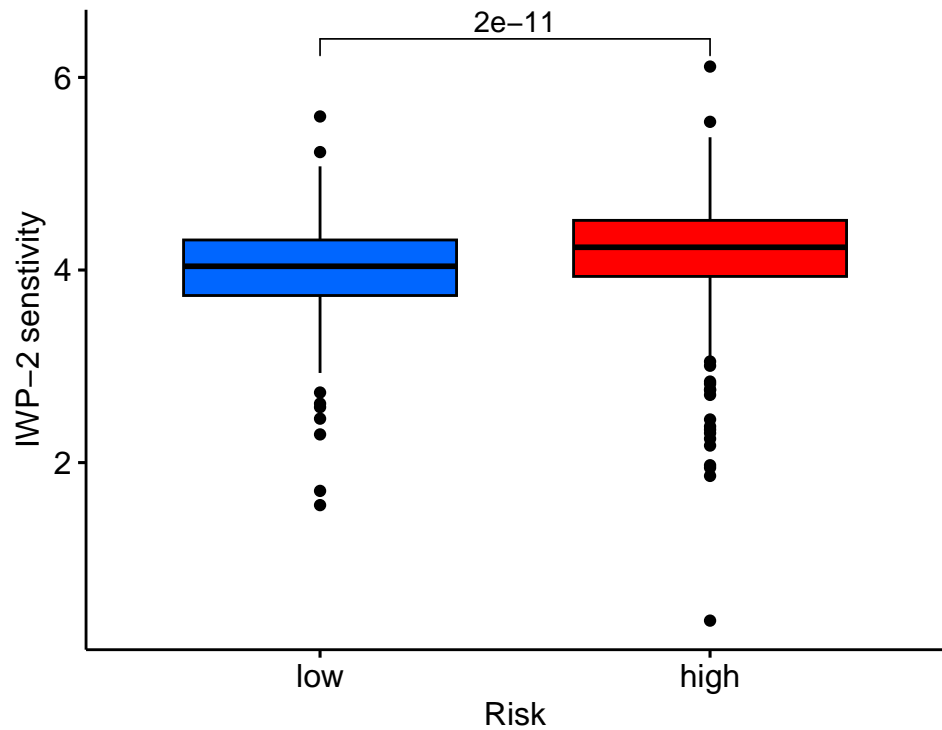

Supplement: Supporting Information 3 — Distinct patterns of drug sensitivity between low-risk and high-risk groups. [file 3423698.f3.zip › Supplementary Material 3/drugSenstivity.IWP-2.pdf]

Risk 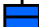 low 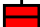 high

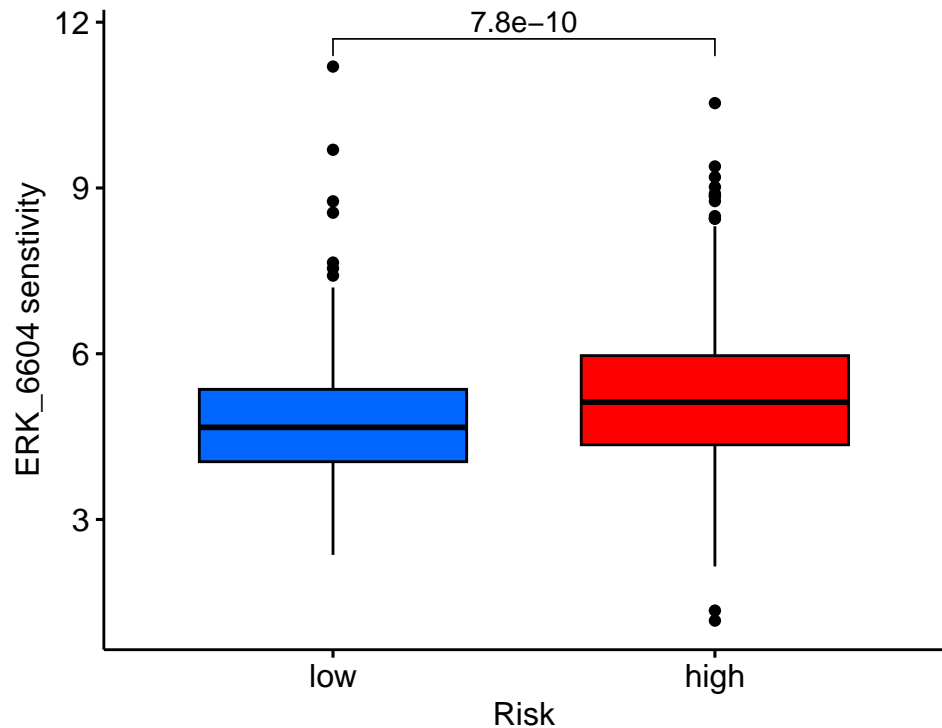

Supplement: Supporting Information 3 — Distinct patterns of drug sensitivity between low-risk and high-risk groups. [file 3423698.f3.zip › Supplementary Material 3/drugSenstivity.ERK_6604.pdf]

Risk 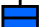 low 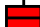 high

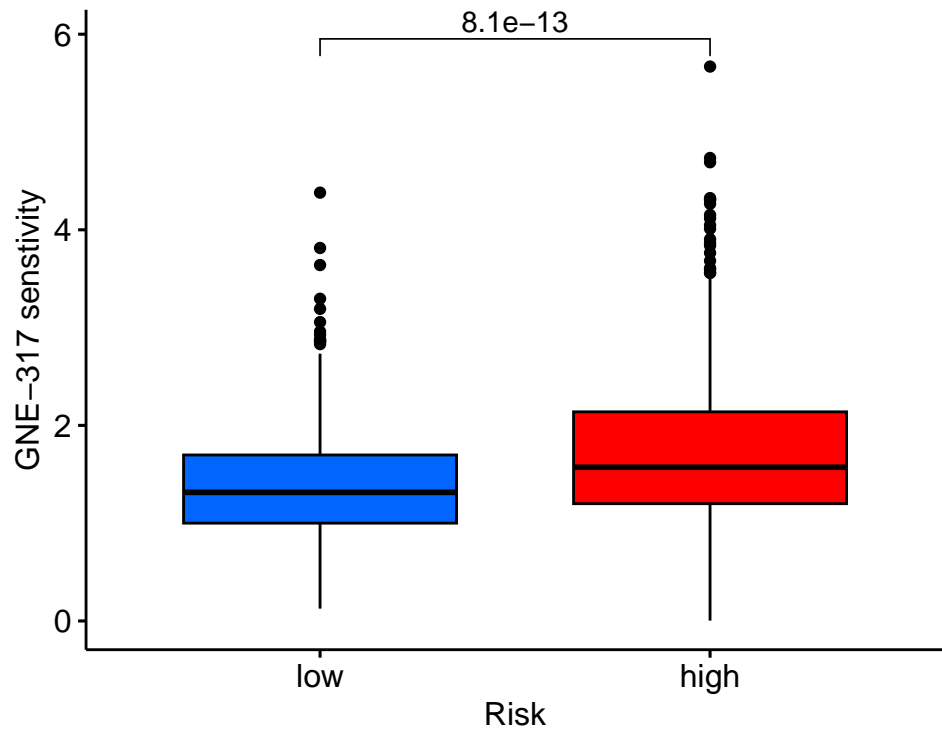

Supplement: Supporting Information 3 — Distinct patterns of drug sensitivity between low-risk and high-risk groups. [file 3423698.f3.zip › Supplementary Material 3/drugSenstivity.GNE-317.pdf]

Risk 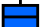 low 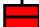 high

$p < 2.22e-16$

MIRA-1 sensitivity

10.0

7.5

5.0

2.5

0.0

low

high

Risk

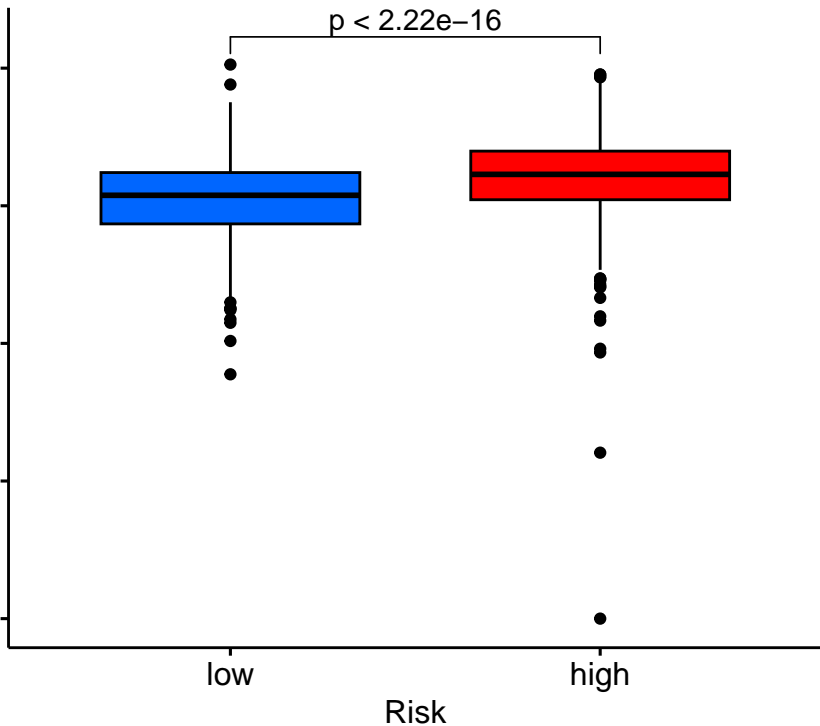

Supplement: Supporting Information 3 — Distinct patterns of drug sensitivity between low-risk and high-risk groups. [file 3423698.f3.zip › Supplementary Material 3/drugSenstivity.MIRA-1.pdf]

Risk 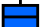 low 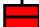 high

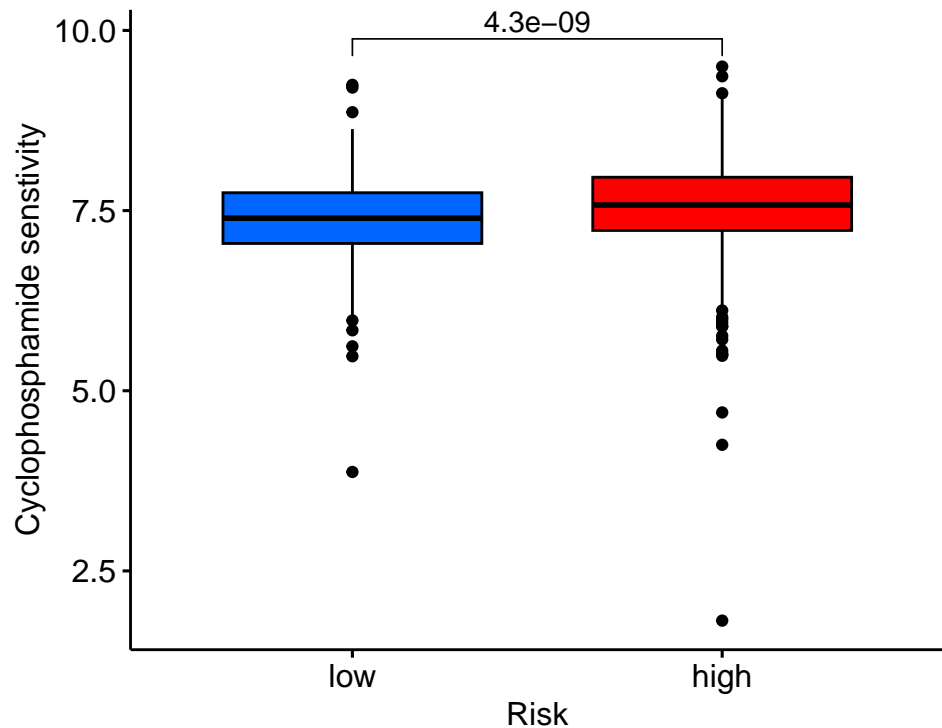

Supplement: Supporting Information 3 — Distinct patterns of drug sensitivity between low-risk and high-risk groups. [file 3423698.f3.zip › Supplementary Material 3/drugSenstivity.Cyclophosphamide.pdf]

Risk 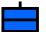 low 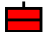 high

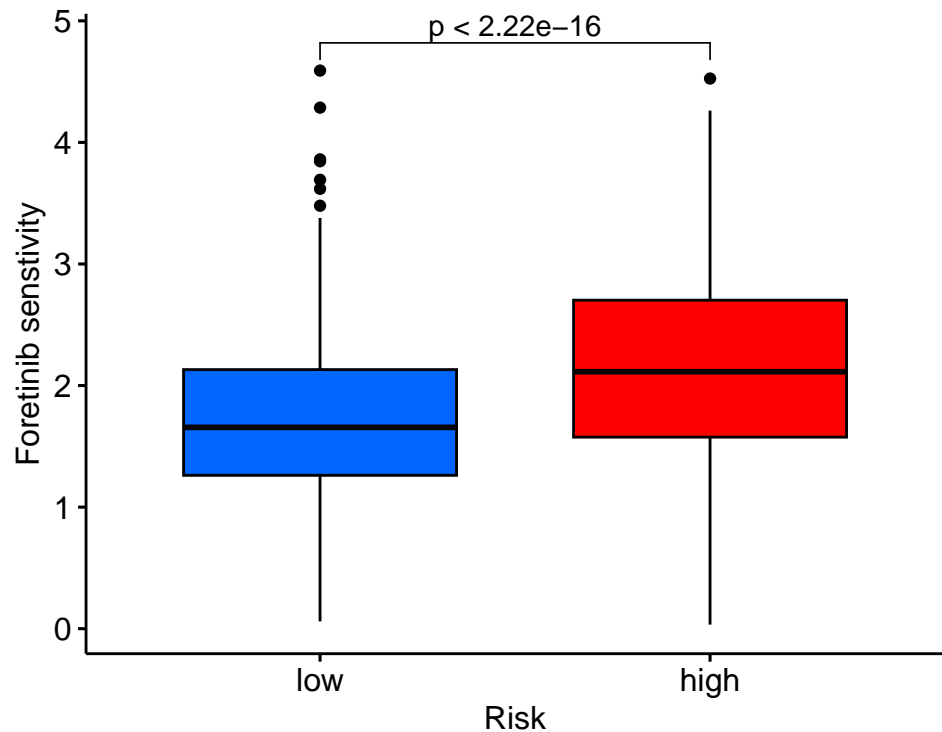

Supplement: Supporting Information 3 — Distinct patterns of drug sensitivity between low-risk and high-risk groups. [file 3423698.f3.zip › Supplementary Material 3/drugSenstivity.Foretinib.pdf]
