# Supplementary figures and images for: The Role of PANoptosis-Related Genes in Predicting Breast Cancer Survival and Immune Prospect (part 2 of 2)
Source: Biomed Res Int. 2025 May 28;2025:3423698. doi: 10.1155/bmri/3423698 (PMC12136870; doi:10.1155/bmri/3423698)

Risk 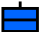 low 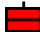 high

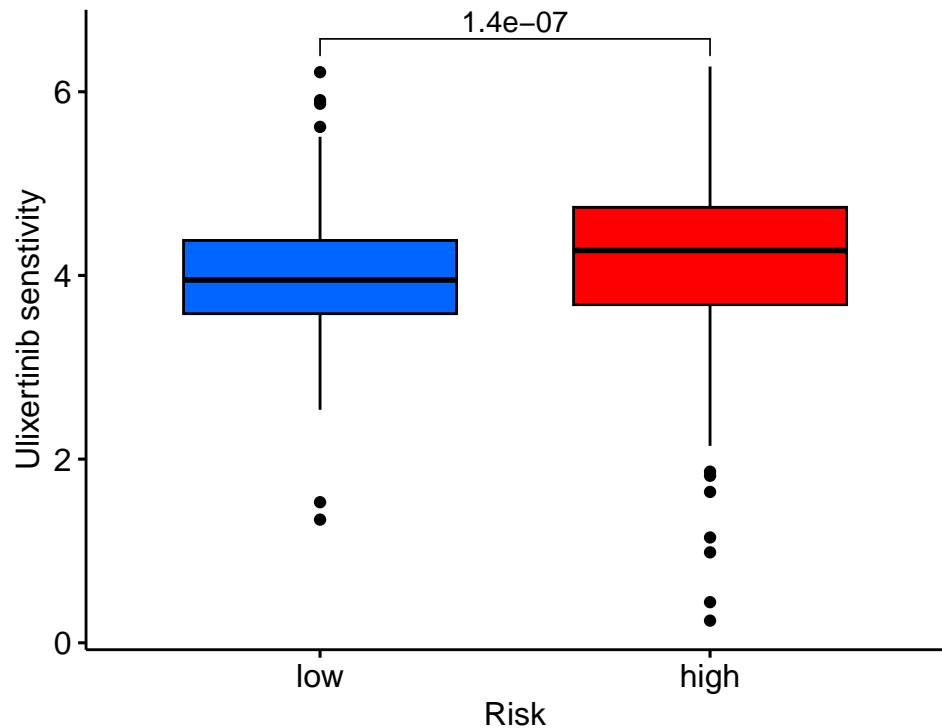

Supplement: Supporting Information 3 — Distinct patterns of drug sensitivity between low-risk and high-risk groups. [file 3423698.f3.zip › Supplementary Material 3/drugSenstivity.Ulixertinib.pdf]

Risk 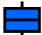 low 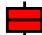 high

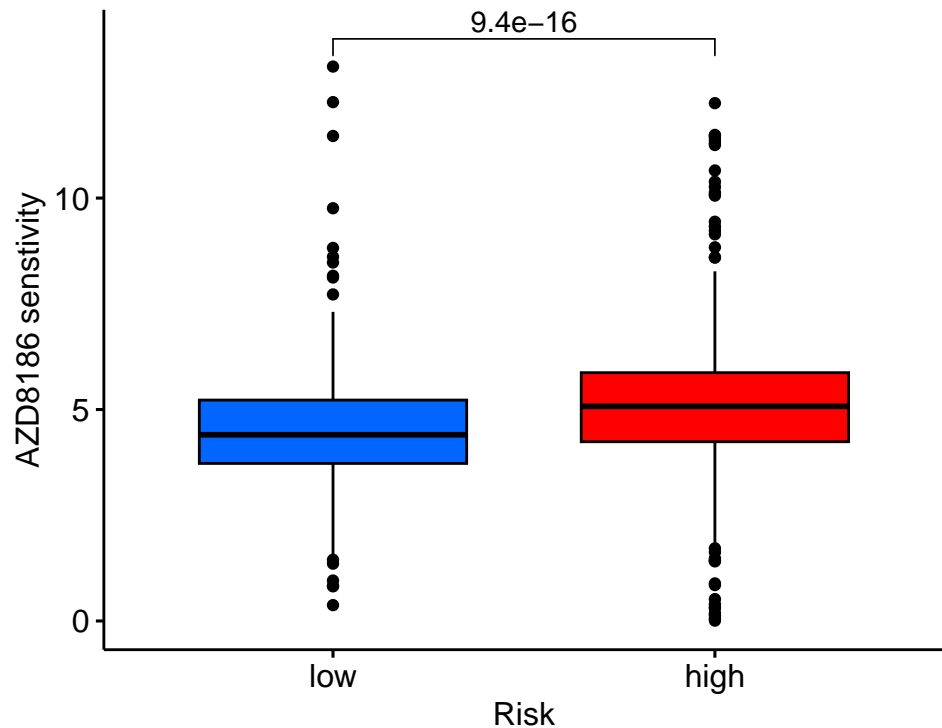

Supplement: Supporting Information 3 — Distinct patterns of drug sensitivity between low-risk and high-risk groups. [file 3423698.f3.zip › Supplementary Material 3/drugSenstivity.AZD8186.pdf]

Risk 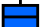 low 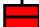 high

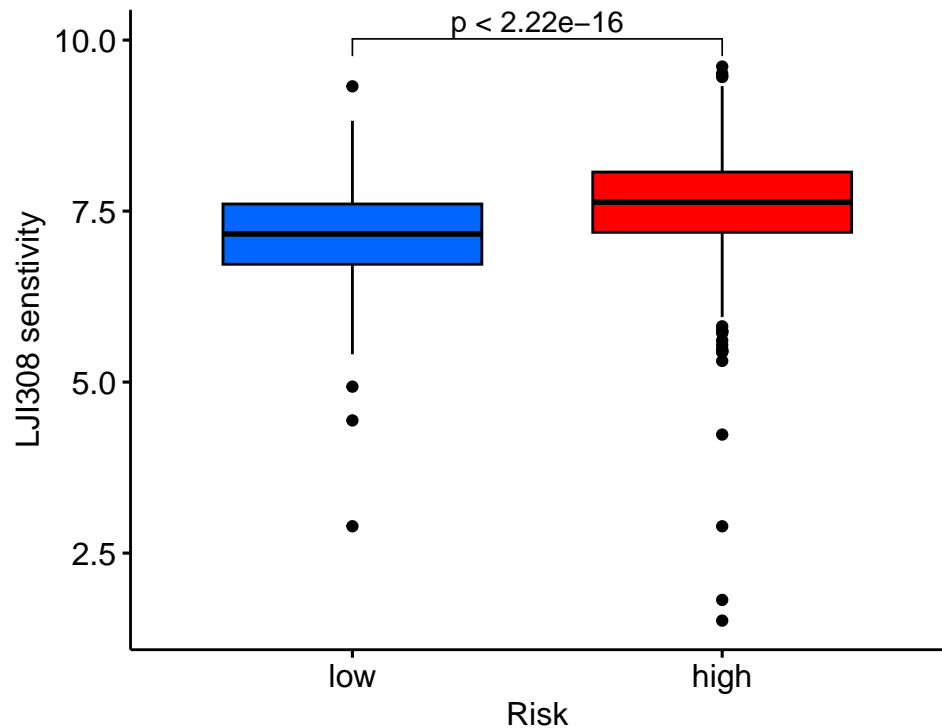

Supplement: Supporting Information 3 — Distinct patterns of drug sensitivity between low-risk and high-risk groups. [file 3423698.f3.zip › Supplementary Material 3/drugSenstivity.LJI308.pdf]

Risk 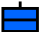 low 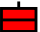 high

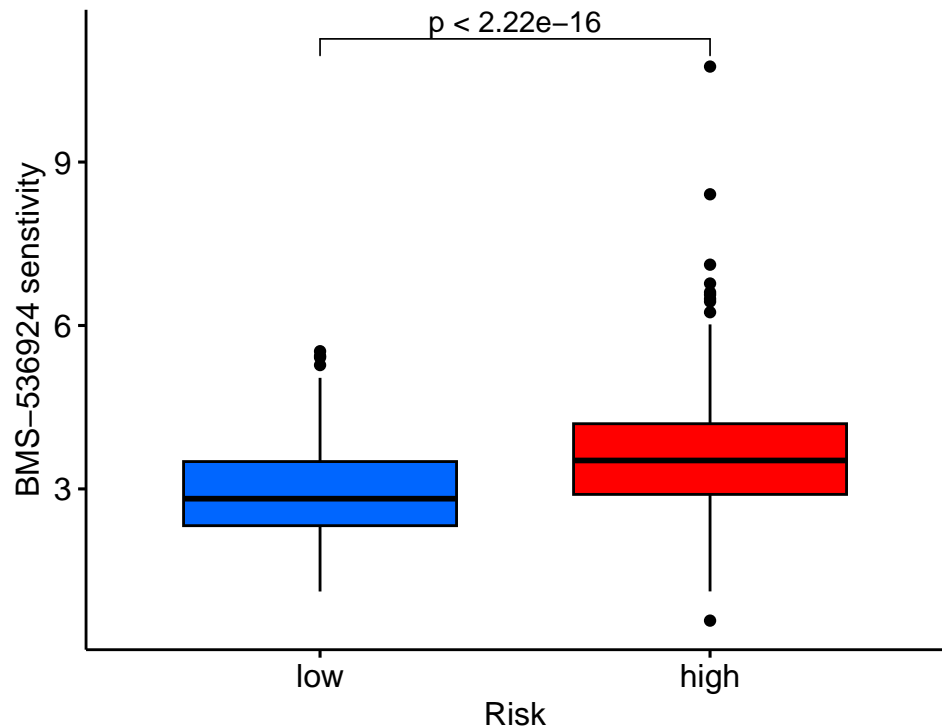

Supplement: Supporting Information 3 — Distinct patterns of drug sensitivity between low-risk and high-risk groups. [file 3423698.f3.zip › Supplementary Material 3/drugSenstivity.BMS-536924.pdf]

Risk 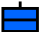 low 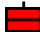 high

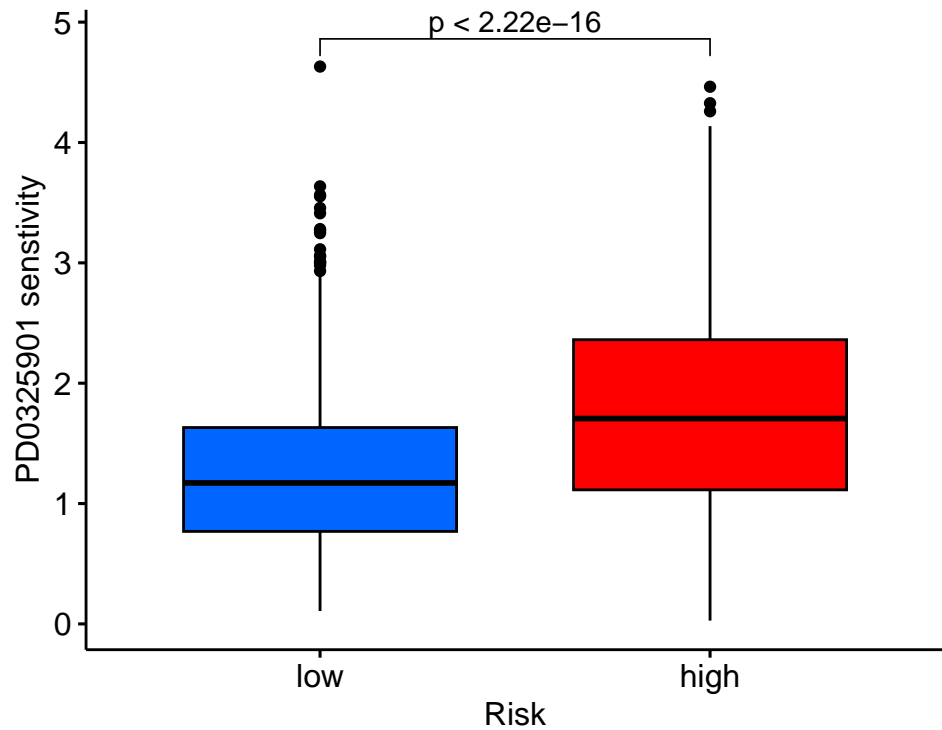

Supplement: Supporting Information 3 — Distinct patterns of drug sensitivity between low-risk and high-risk groups. [file 3423698.f3.zip › Supplementary Material 3/drugSenstivity.PD0325901.pdf]

Risk 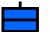 low 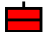 high

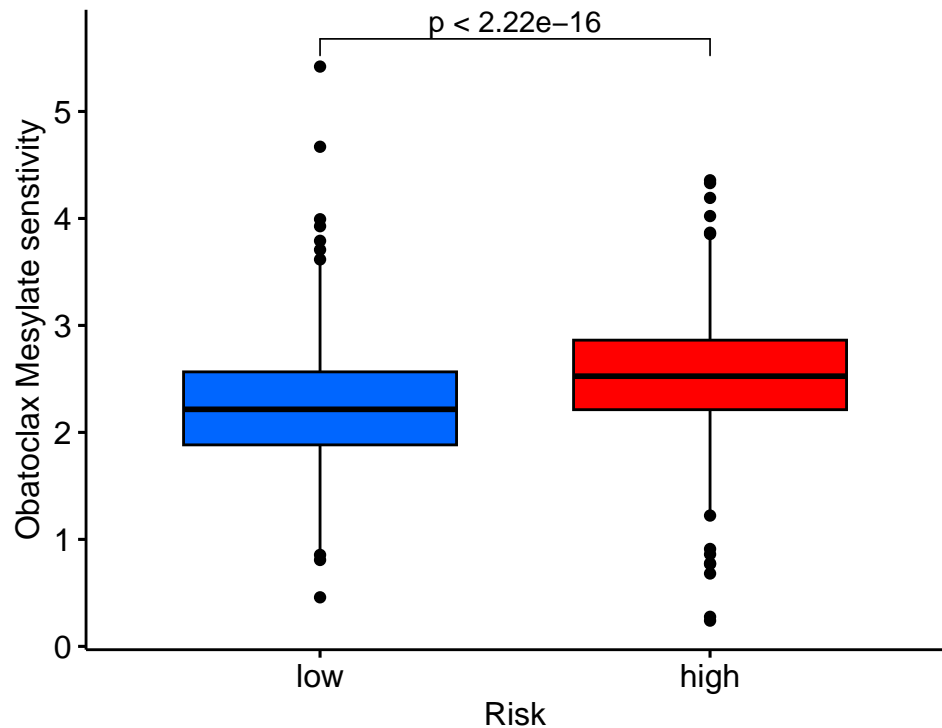

Supplement: Supporting Information 3 — Distinct patterns of drug sensitivity between low-risk and high-risk groups. [file 3423698.f3.zip › Supplementary Material 3/drugSenstivity.Obatoclax Mesylate.pdf]

Risk low high

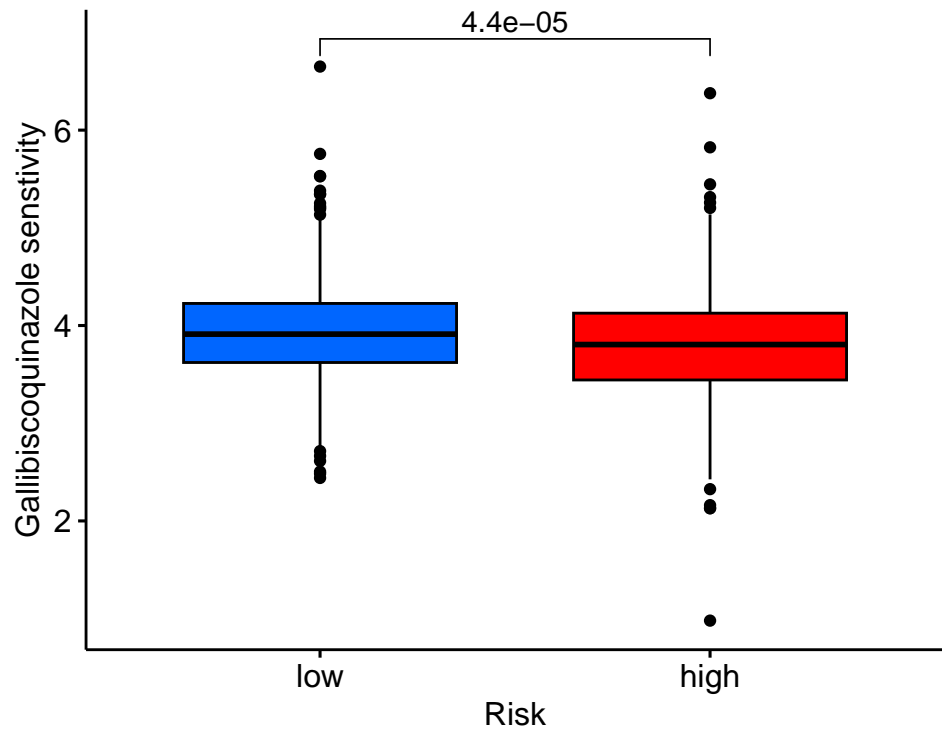

Supplement: Supporting Information 3 — Distinct patterns of drug sensitivity between low-risk and high-risk groups. [file 3423698.f3.zip › Supplementary Material 3/drugSenstivity.Gallibiscoquinazole.pdf]

Risk low high

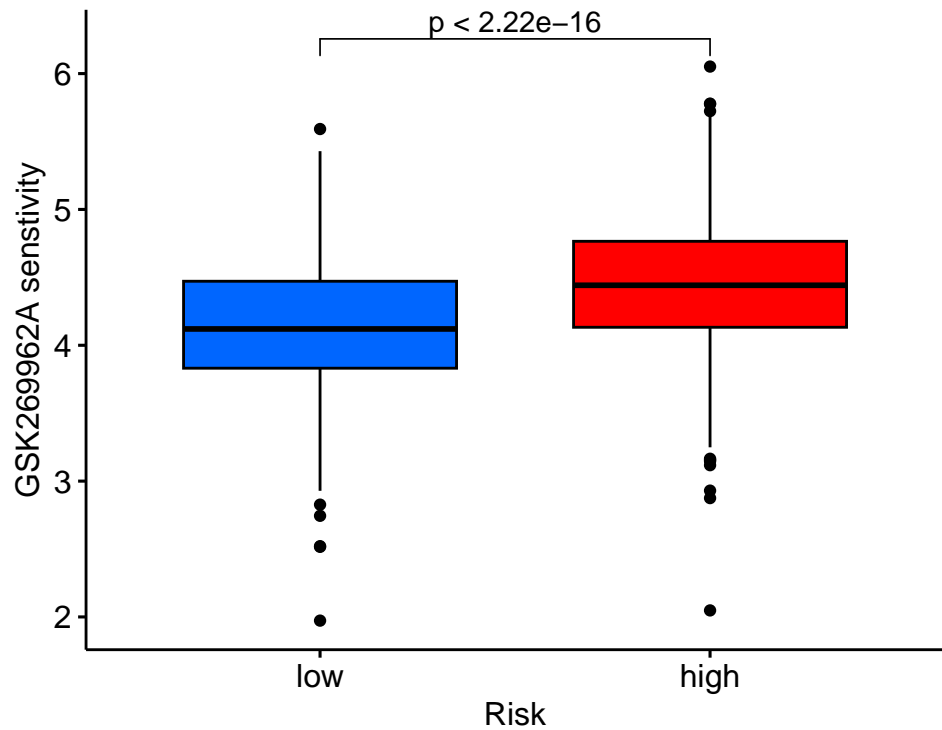

Supplement: Supporting Information 3 — Distinct patterns of drug sensitivity between low-risk and high-risk groups. [file 3423698.f3.zip › Supplementary Material 3/drugSenstivity.GSK269962A.pdf]

Risk 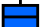 low 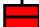 high

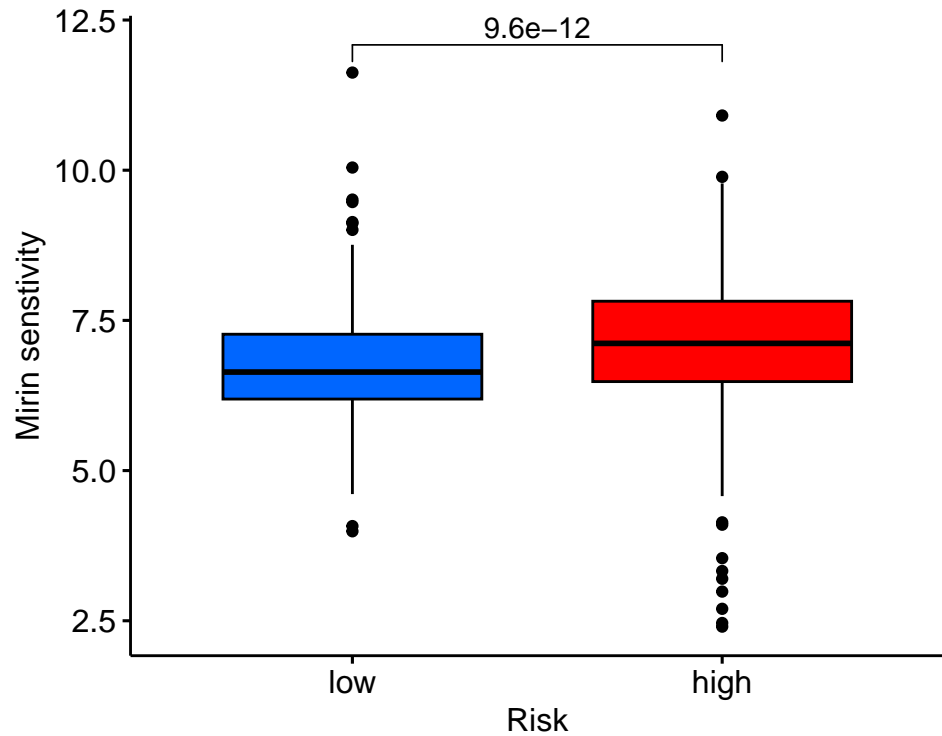

Supplement: Supporting Information 3 — Distinct patterns of drug sensitivity between low-risk and high-risk groups. [file 3423698.f3.zip › Supplementary Material 3/drugSenstivity.Mirin.pdf]

Risk 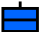 low 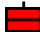 high

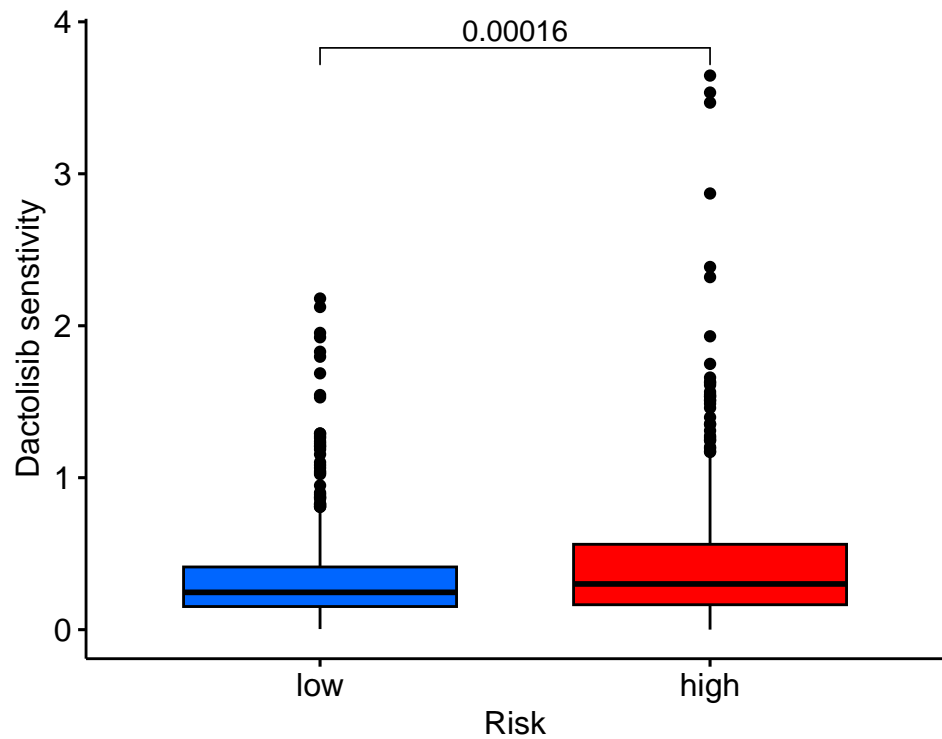

Supplement: Supporting Information 3 — Distinct patterns of drug sensitivity between low-risk and high-risk groups. [file 3423698.f3.zip › Supplementary Material 3/drugSenstivity.Dactolisib.pdf]

Risk 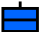 low 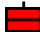 high

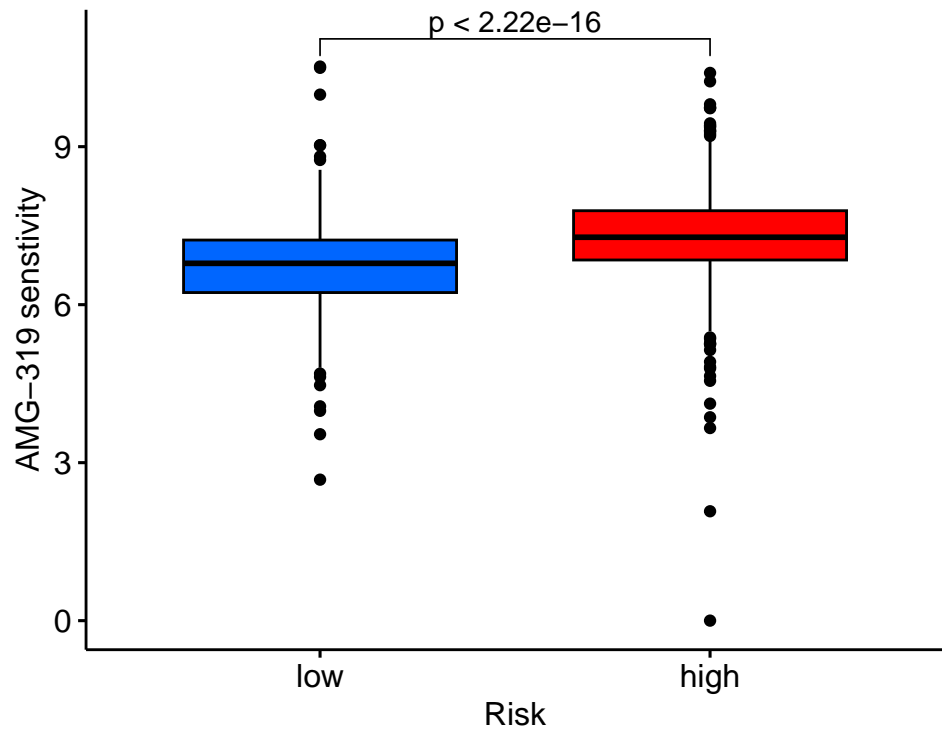

Supplement: Supporting Information 3 — Distinct patterns of drug sensitivity between low-risk and high-risk groups. [file 3423698.f3.zip › Supplementary Material 3/drugSenstivity.AMG-319.pdf]

Risk 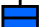 low 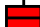 high

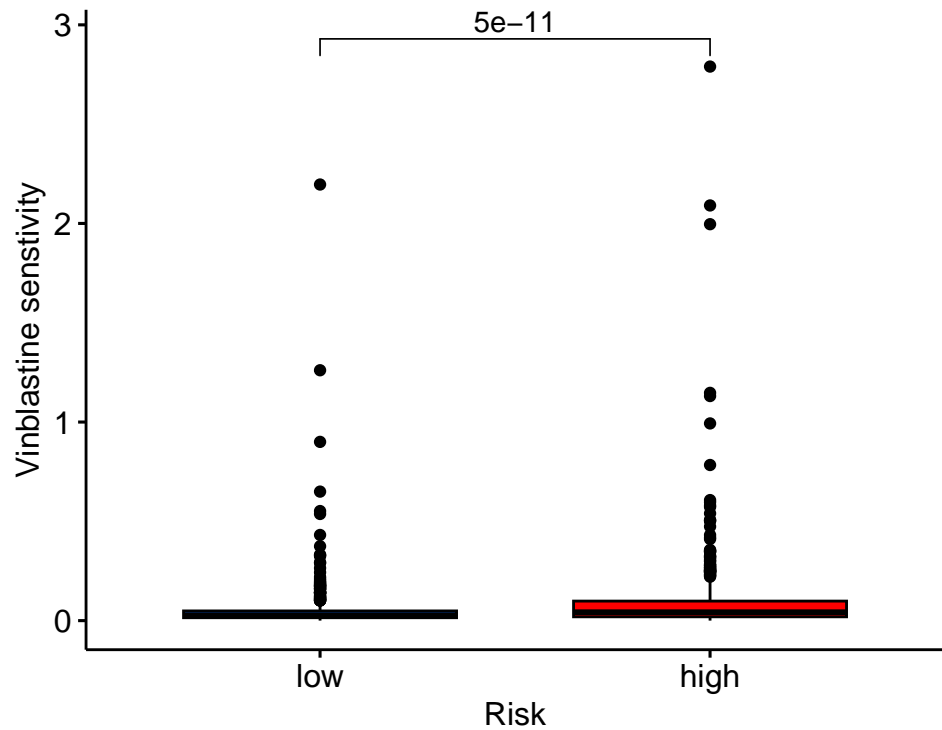

Supplement: Supporting Information 3 — Distinct patterns of drug sensitivity between low-risk and high-risk groups. [file 3423698.f3.zip › Supplementary Material 3/drugSenstivity.Vinblastine.pdf]

Risk 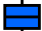 low 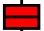 high

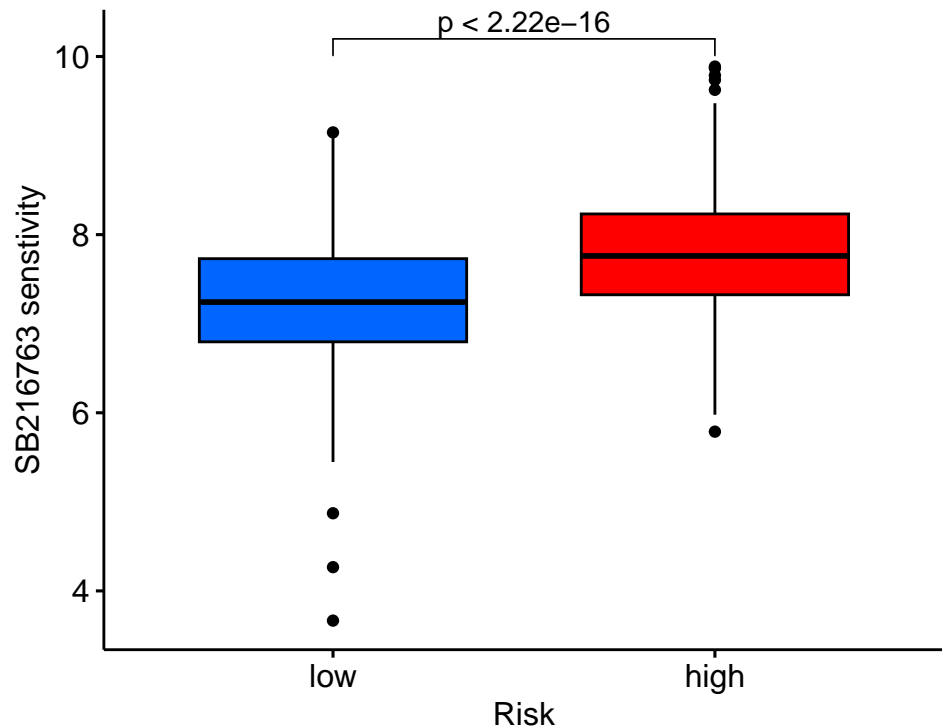

Supplement: Supporting Information 3 — Distinct patterns of drug sensitivity between low-risk and high-risk groups. [file 3423698.f3.zip › Supplementary Material 3/drugSenstivity.SB216763.pdf]

Risk 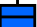 low 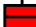 high

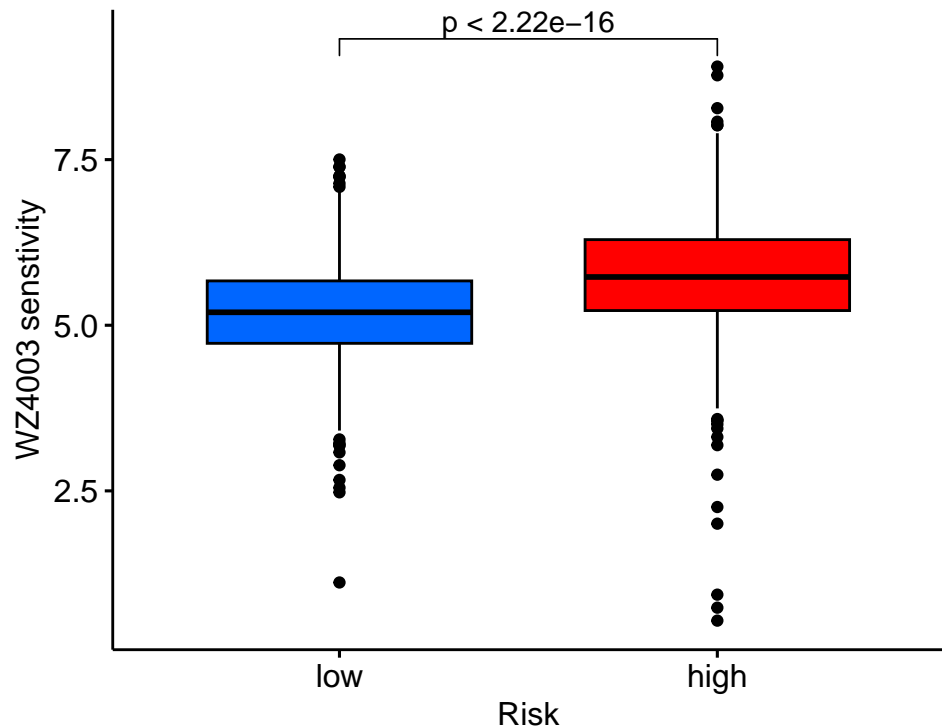

Supplement: Supporting Information 3 — Distinct patterns of drug sensitivity between low-risk and high-risk groups. [file 3423698.f3.zip › Supplementary Material 3/drugSenstivity.WZ4003.pdf]

Risk 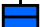 low 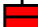 high

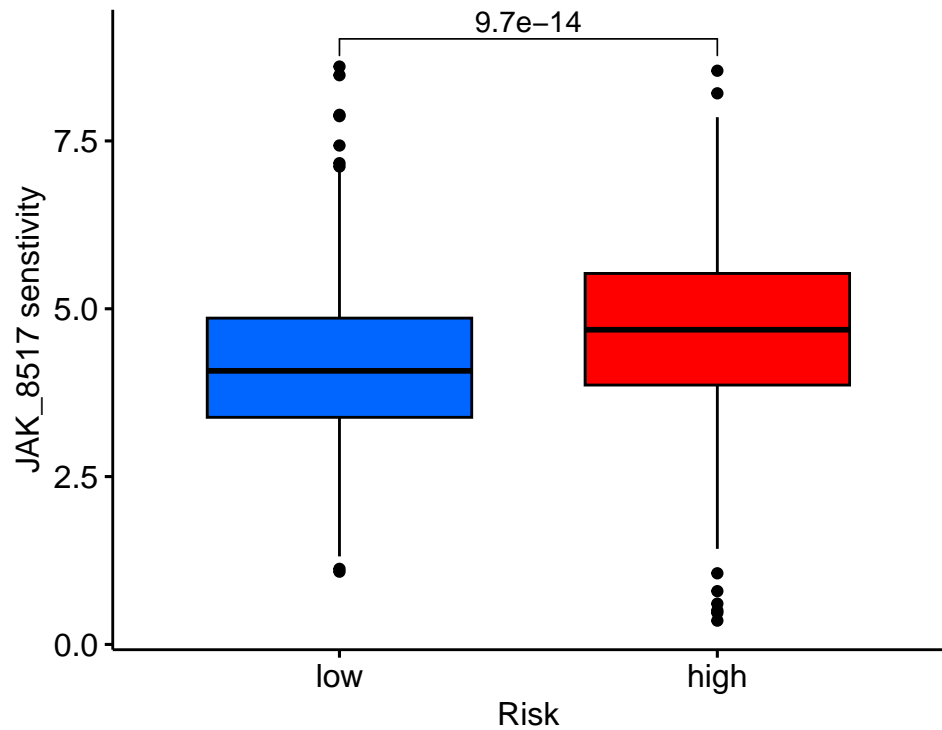

Supplement: Supporting Information 3 — Distinct patterns of drug sensitivity between low-risk and high-risk groups. [file 3423698.f3.zip › Supplementary Material 3/drugSenstivity.JAK_8517.pdf]

Risk 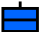 low 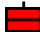 high

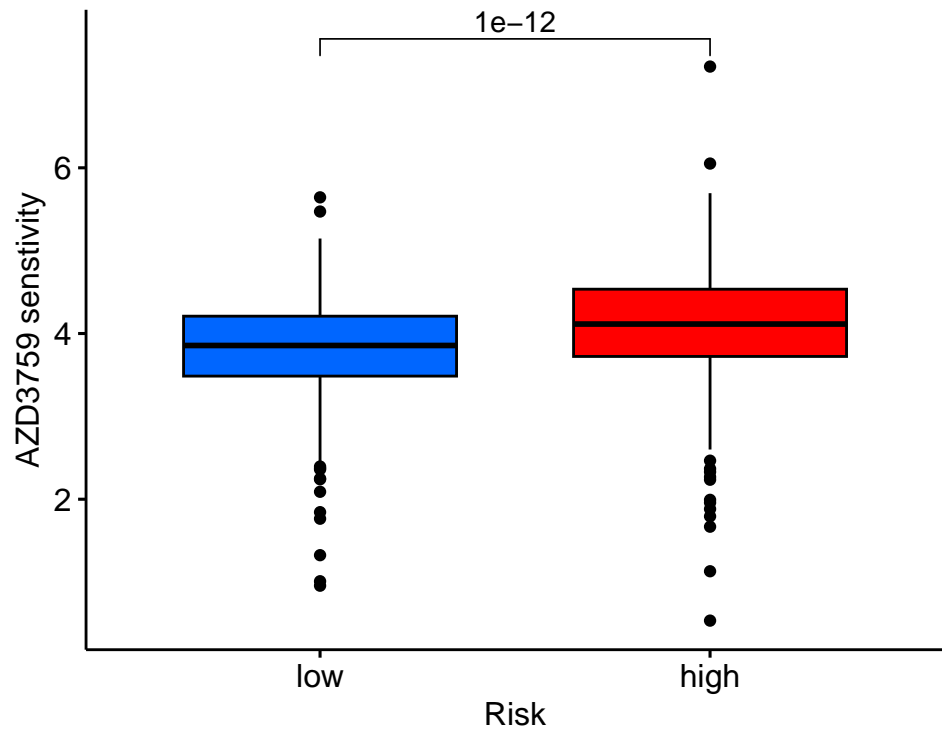

Supplement: Supporting Information 3 — Distinct patterns of drug sensitivity between low-risk and high-risk groups. [file 3423698.f3.zip › Supplementary Material 3/drugSenstivity.AZD3759.pdf]

Risk 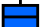 low 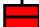 high

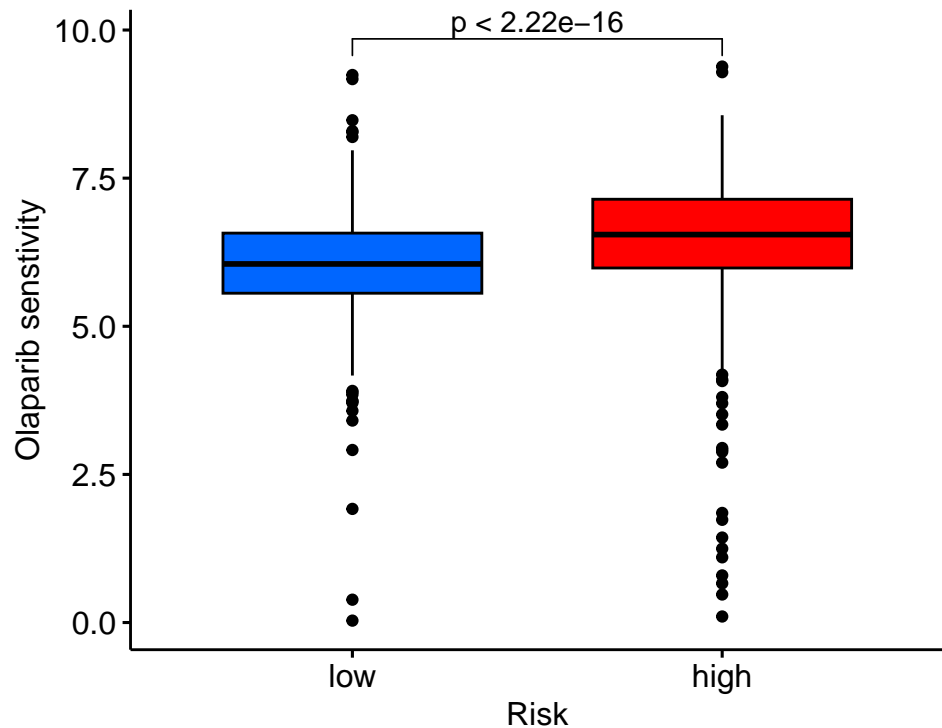

Supplement: Supporting Information 3 — Distinct patterns of drug sensitivity between low-risk and high-risk groups. [file 3423698.f3.zip › Supplementary Material 3/drugSenstivity.Olaparib.pdf]

Risk 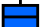 low 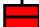 high

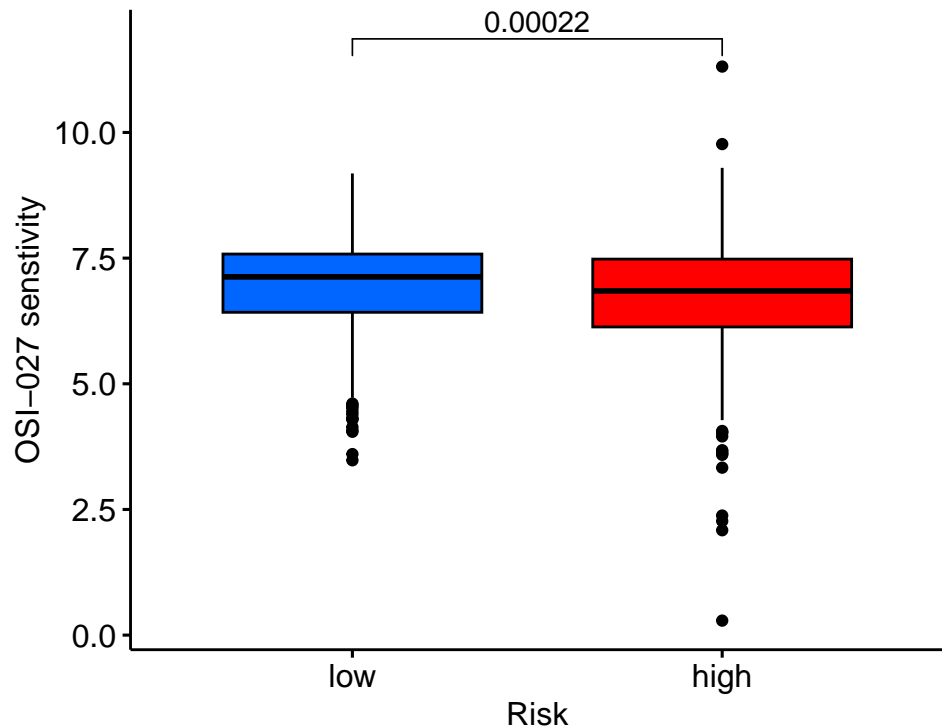

Supplement: Supporting Information 3 — Distinct patterns of drug sensitivity between low-risk and high-risk groups. [file 3423698.f3.zip › Supplementary Material 3/drugSenstivity.OSI-027.pdf]

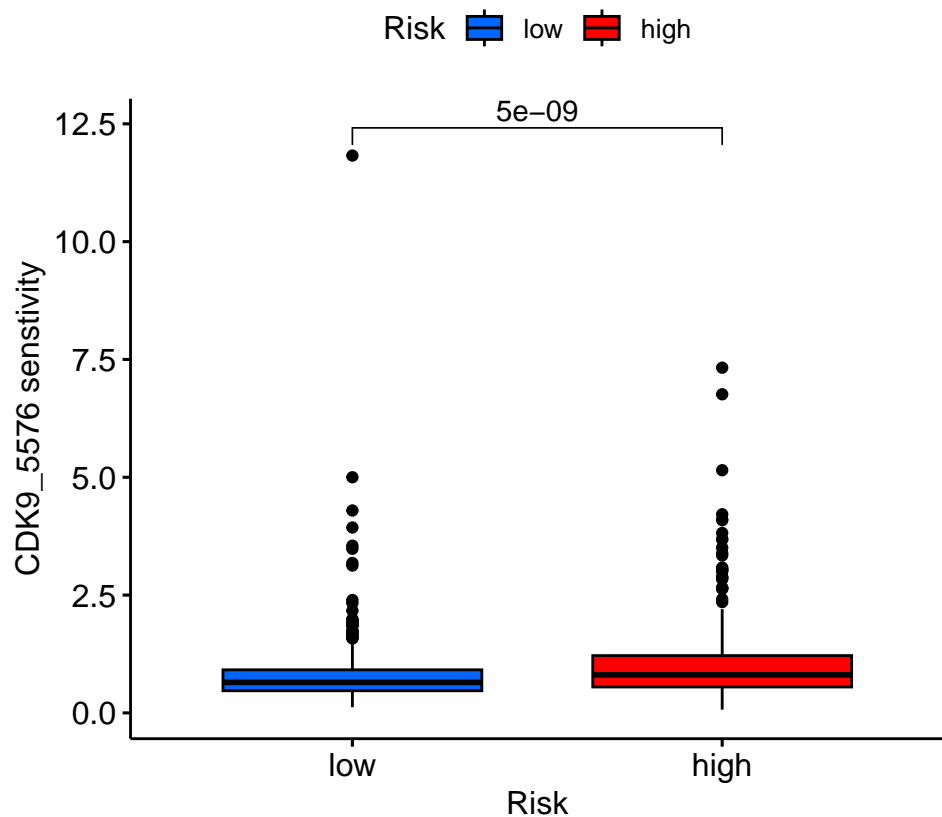

Supplement: Supporting Information 3 — Distinct patterns of drug sensitivity between low-risk and high-risk groups. [file 3423698.f3.zip › Supplementary Material 3/drugSenstivity.CDK9_5576.pdf]

Risk low high

$p < 2.22\text{e-}16$

YK-4-279 sensitivity

low

high

Risk

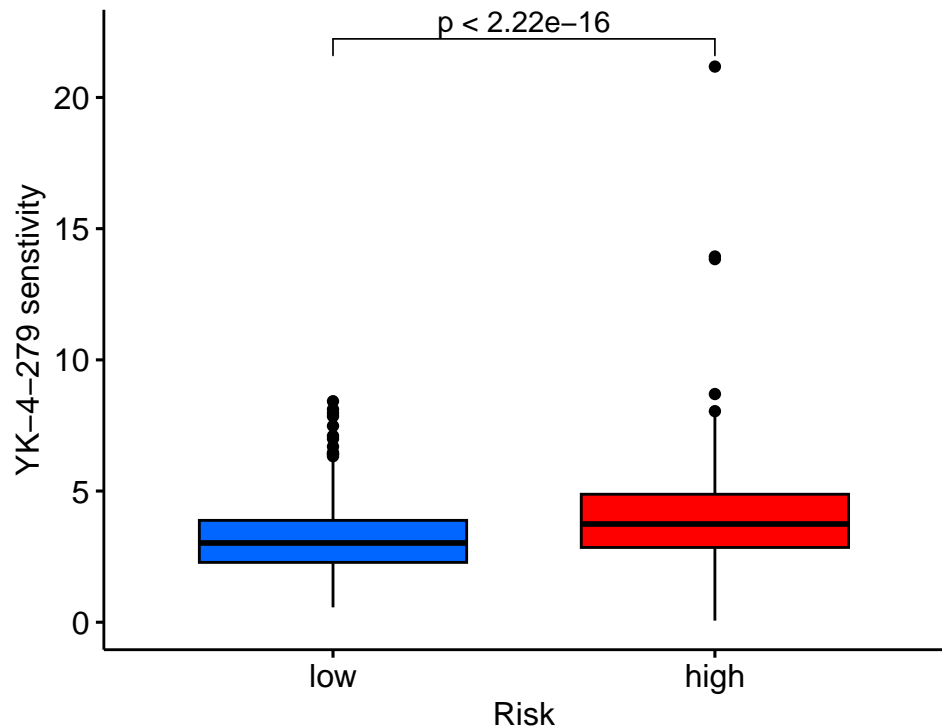

Supplement: Supporting Information 3 — Distinct patterns of drug sensitivity between low-risk and high-risk groups. [file 3423698.f3.zip › Supplementary Material 3/drugSenstivity.YK-4-279.pdf]

Risk 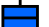 low 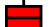 high

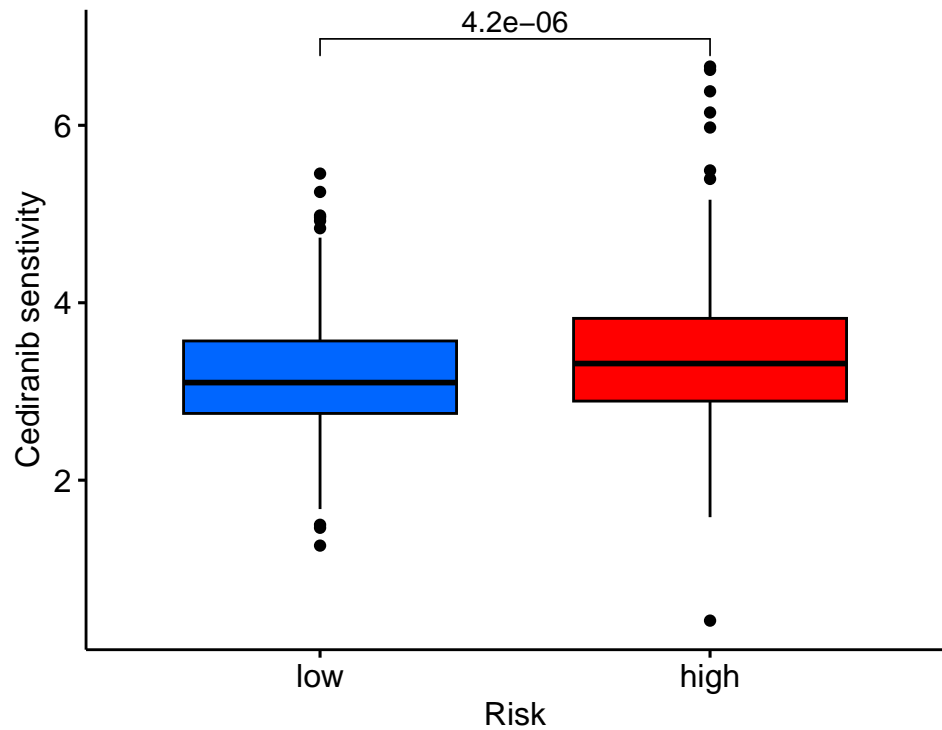

Supplement: Supporting Information 3 — Distinct patterns of drug sensitivity between low-risk and high-risk groups. [file 3423698.f3.zip › Supplementary Material 3/drugSenstivity.Cediranib.pdf]

Risk 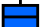 low 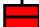 high

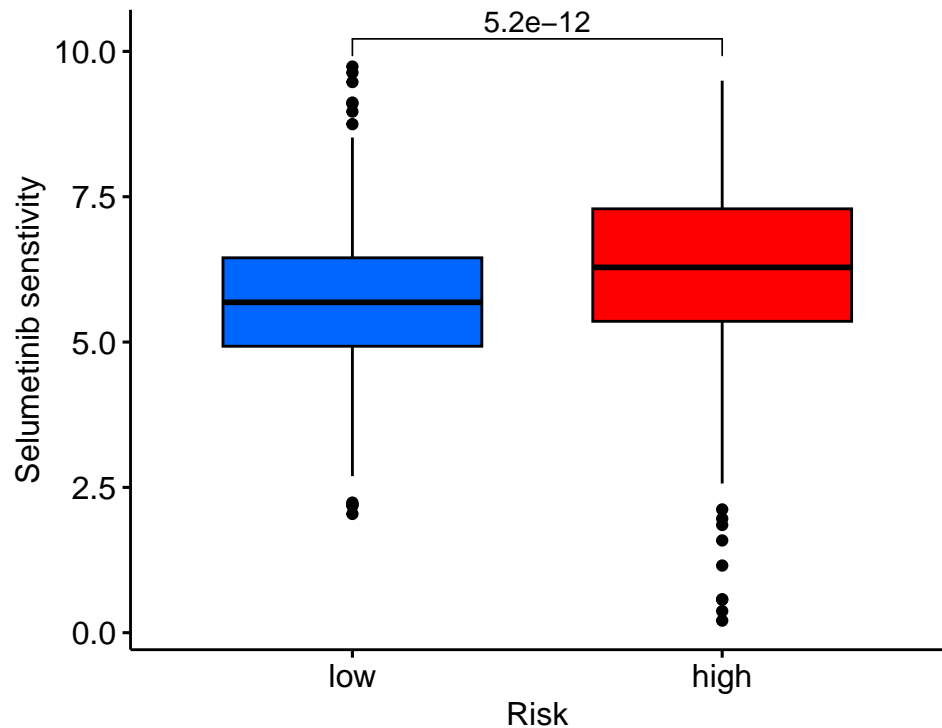

Supplement: Supporting Information 3 — Distinct patterns of drug sensitivity between low-risk and high-risk groups. [file 3423698.f3.zip › Supplementary Material 3/drugSenstivity.Selumetinib.pdf]

Risk 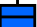 low 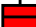 high

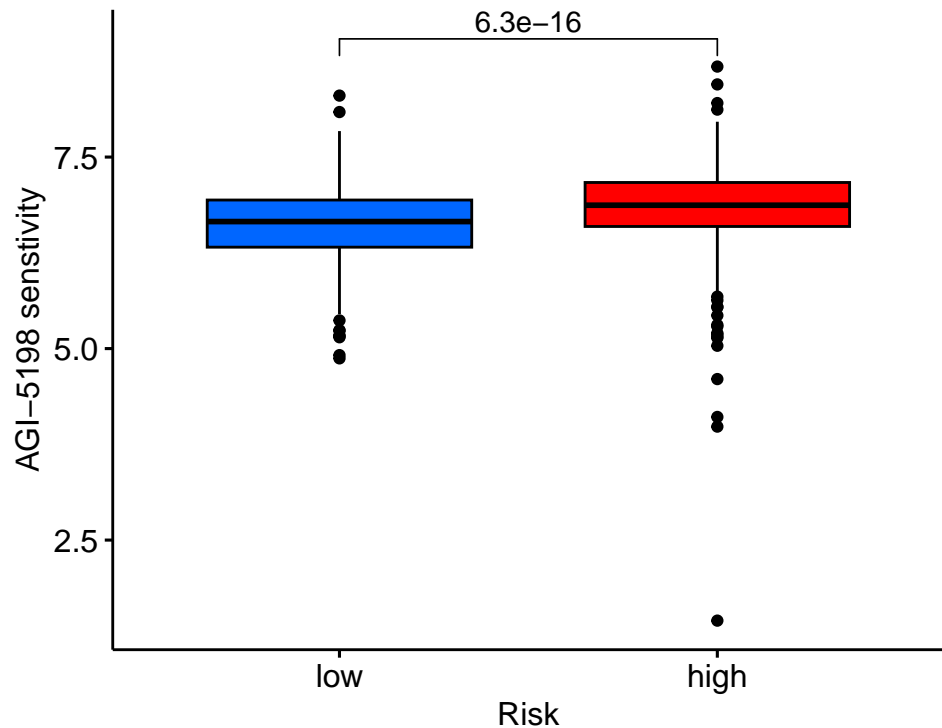

Supplement: Supporting Information 3 — Distinct patterns of drug sensitivity between low-risk and high-risk groups. [file 3423698.f3.zip › Supplementary Material 3/drugSenstivity.AGI-5198.pdf]

Risk 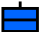 low 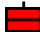 high

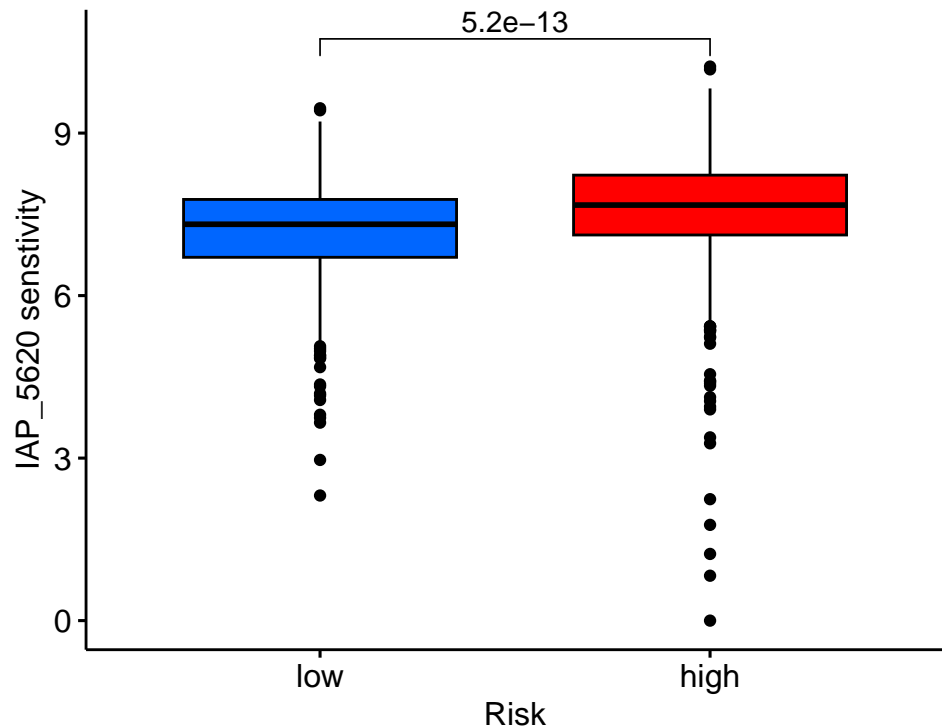

Supplement: Supporting Information 3 — Distinct patterns of drug sensitivity between low-risk and high-risk groups. [file 3423698.f3.zip › Supplementary Material 3/drugSenstivity.IAP_5620.pdf]

Risk 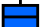 low 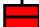 high

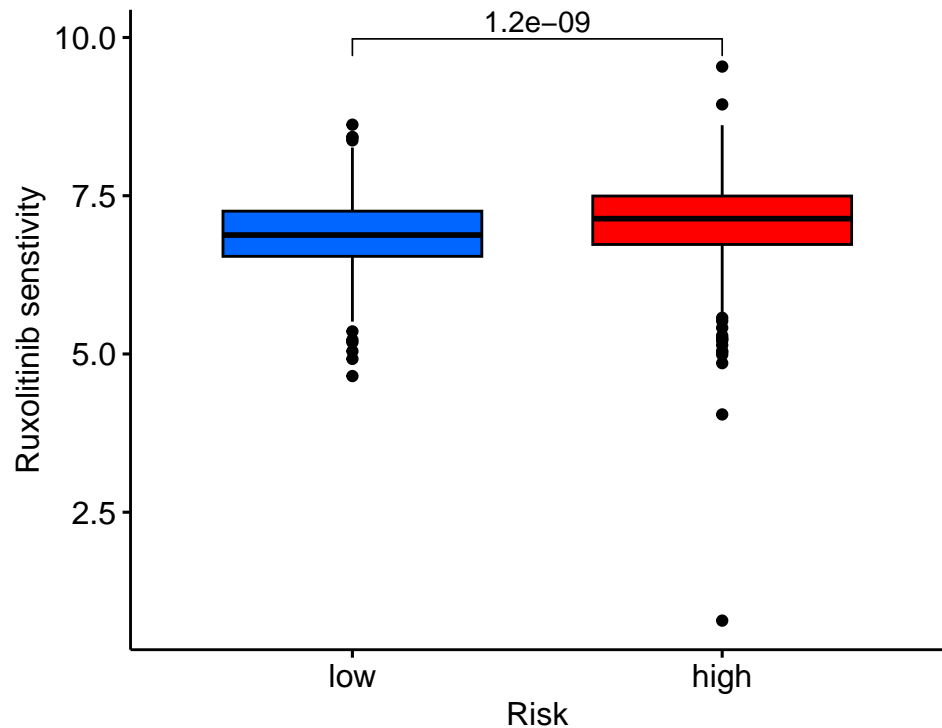

Supplement: Supporting Information 3 — Distinct patterns of drug sensitivity between low-risk and high-risk groups. [file 3423698.f3.zip › Supplementary Material 3/drugSenstivity.Ruxolitinib.pdf]

Risk 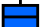 low 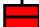 high

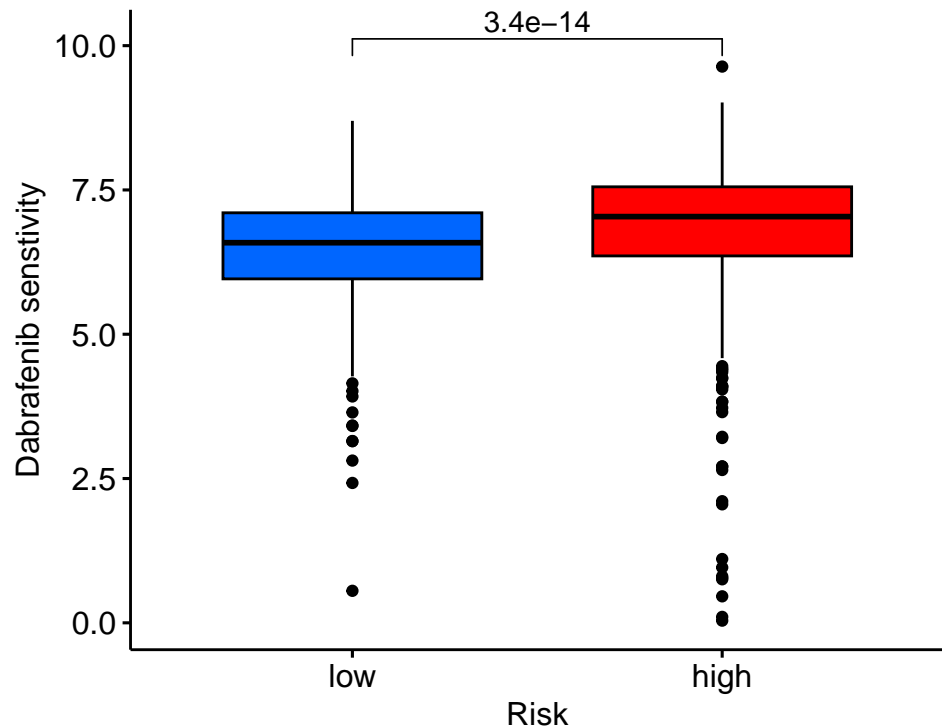

Supplement: Supporting Information 3 — Distinct patterns of drug sensitivity between low-risk and high-risk groups. [file 3423698.f3.zip › Supplementary Material 3/drugSenstivity.Dabrafenib.pdf]

Risk 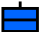 low 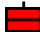 high

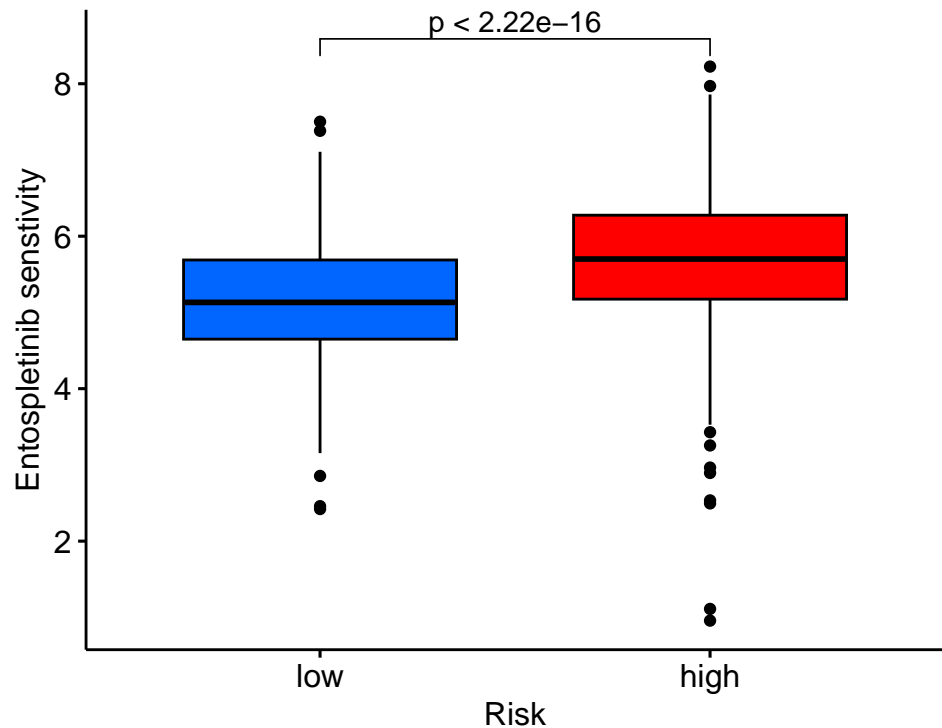

Supplement: Supporting Information 3 — Distinct patterns of drug sensitivity between low-risk and high-risk groups. [file 3423698.f3.zip › Supplementary Material 3/drugSenstivity.Entospletinib.pdf]

Risk 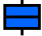 low 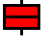 high

$p < 2.22e-16$

Camptothecin sensitivity

10

5

0

low

high

Risk

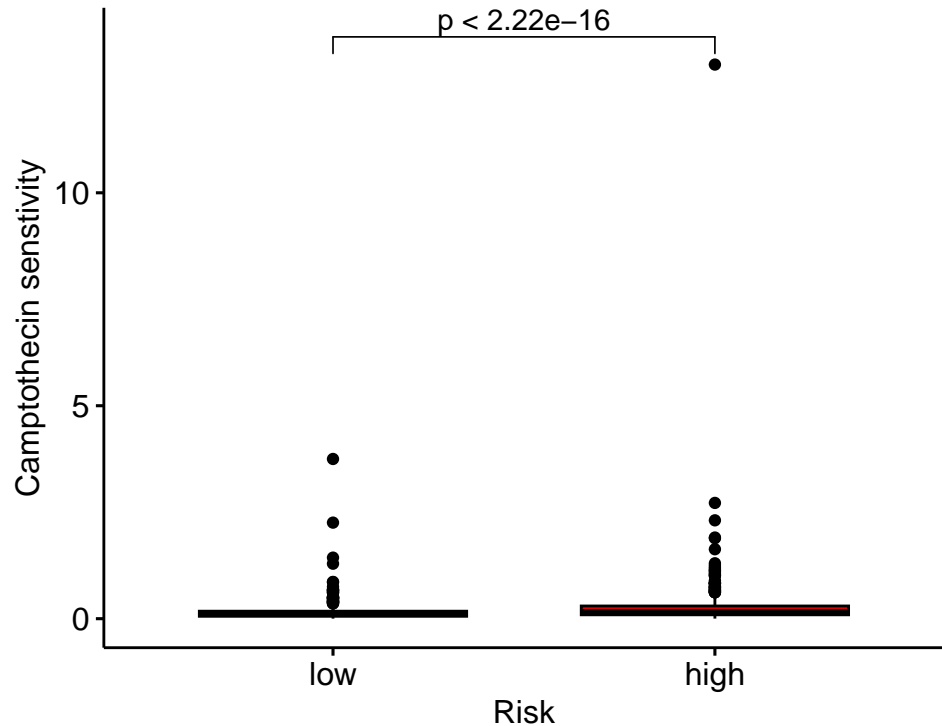

Supplement: Supporting Information 3 — Distinct patterns of drug sensitivity between low-risk and high-risk groups. [file 3423698.f3.zip › Supplementary Material 3/drugSenstivity.Camptothecin.pdf]

Risk 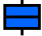 low 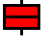 high

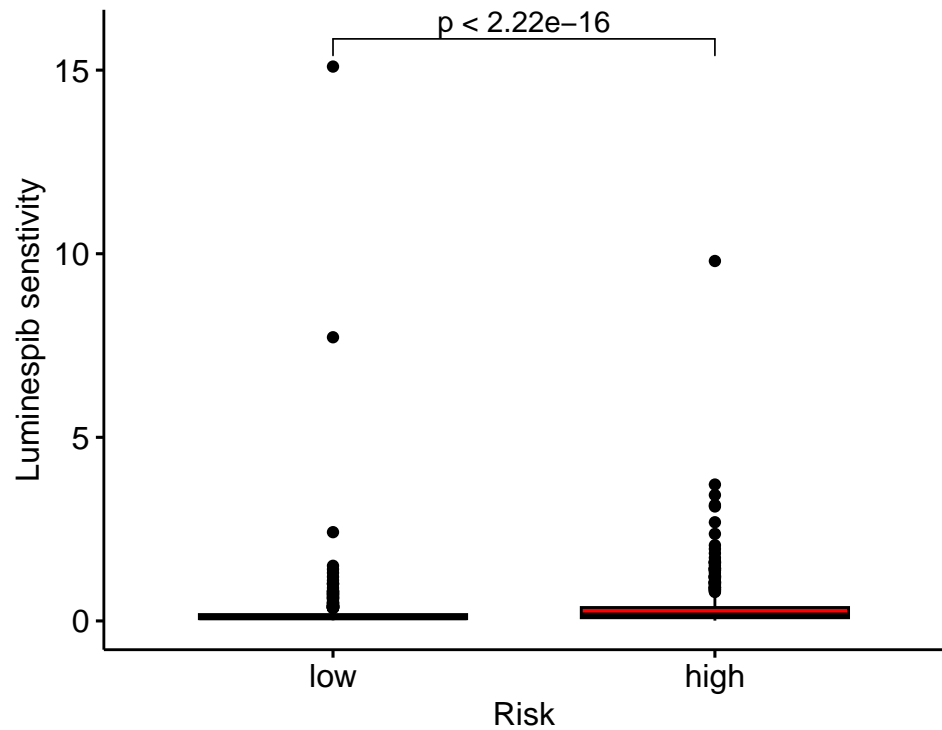

Supplement: Supporting Information 3 — Distinct patterns of drug sensitivity between low-risk and high-risk groups. [file 3423698.f3.zip › Supplementary Material 3/drugSenstivity.Luminespib.pdf]

Risk 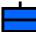 low 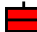 high

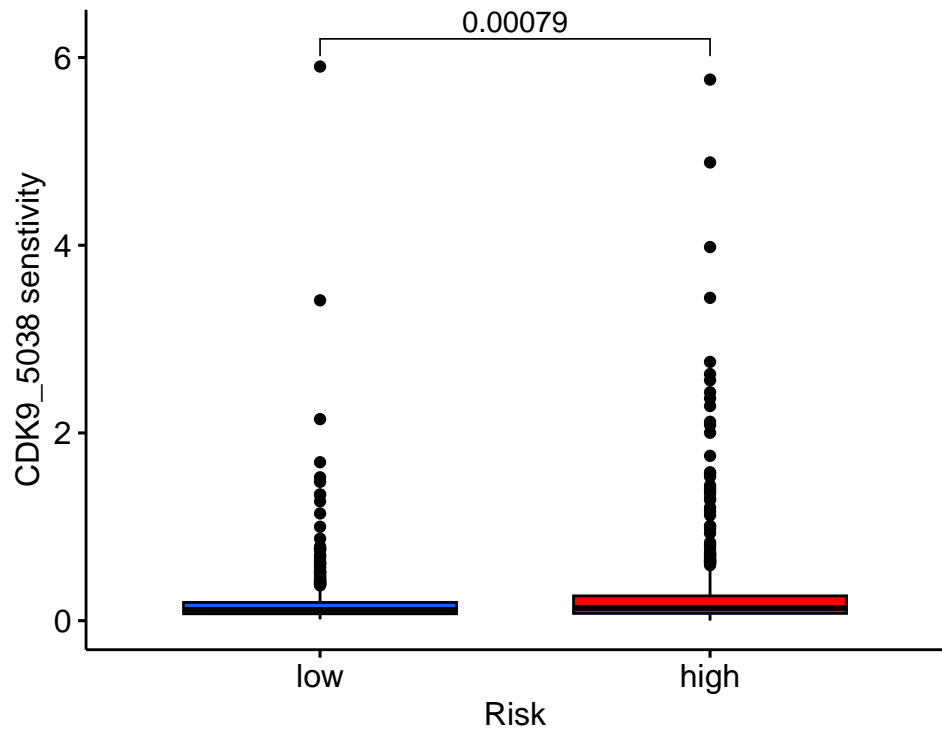

Supplement: Supporting Information 3 — Distinct patterns of drug sensitivity between low-risk and high-risk groups. [file 3423698.f3.zip › Supplementary Material 3/drugSenstivity.CDK9_5038.pdf]

Risk 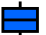 low 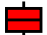 high

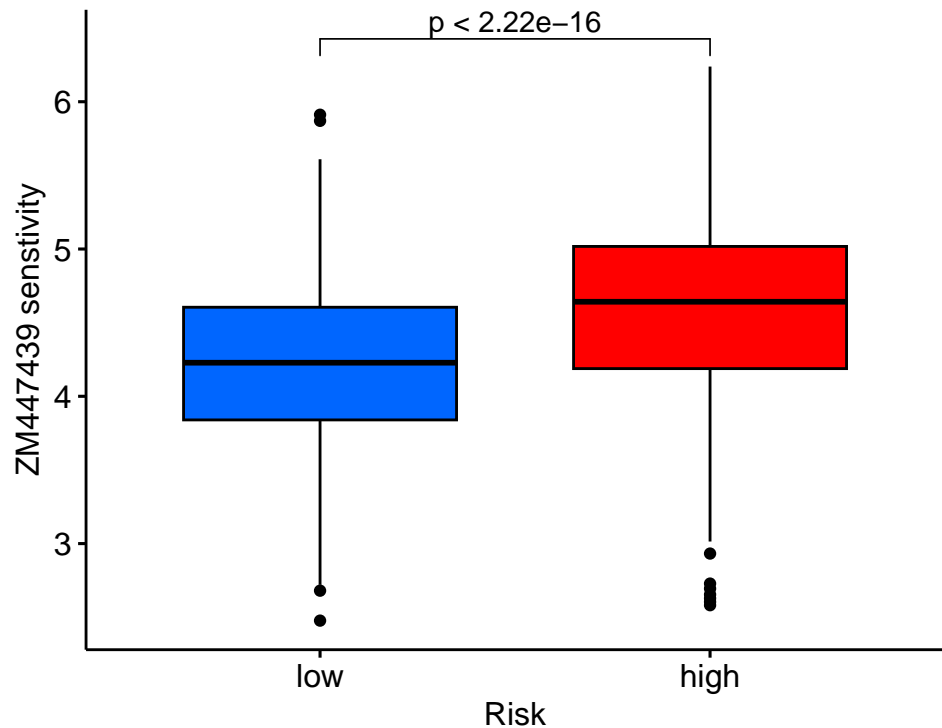

Supplement: Supporting Information 3 — Distinct patterns of drug sensitivity between low-risk and high-risk groups. [file 3423698.f3.zip › Supplementary Material 3/drugSenstivity.ZM447439.pdf]

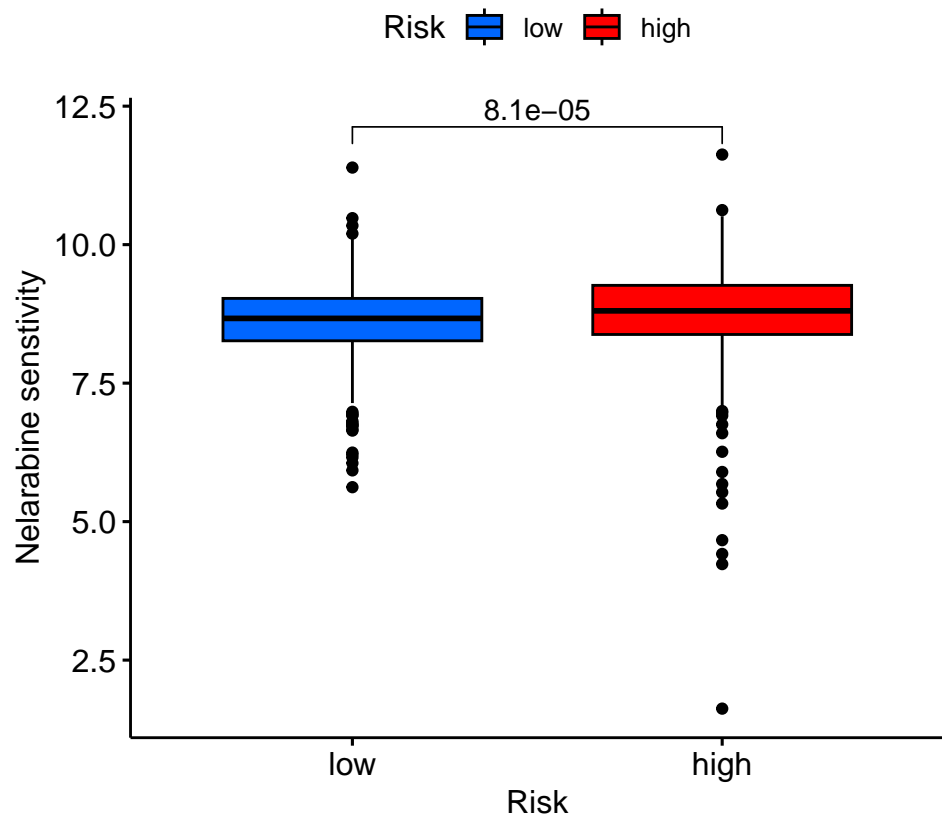

Supplement: Supporting Information 3 — Distinct patterns of drug sensitivity between low-risk and high-risk groups. [file 3423698.f3.zip › Supplementary Material 3/drugSenstivity.Nelarabine.pdf]

EPZ004777 sensitivity

Risk 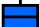 low 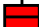 high

$2.4\text{e-}12$

low

high

Risk

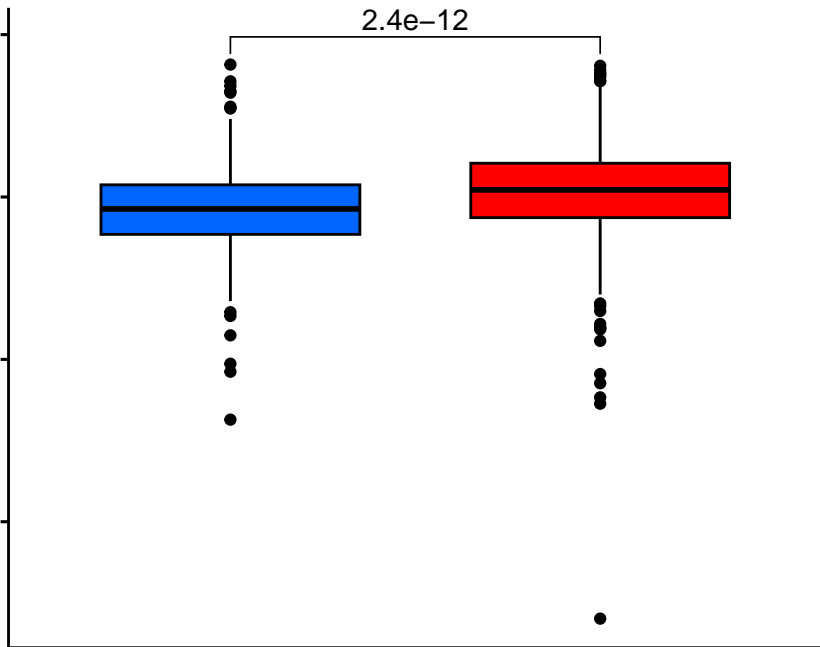

Supplement: Supporting Information 3 — Distinct patterns of drug sensitivity between low-risk and high-risk groups. [file 3423698.f3.zip › Supplementary Material 3/drugSenstivity.EPZ004777.pdf]

Risk 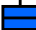 low 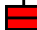 high

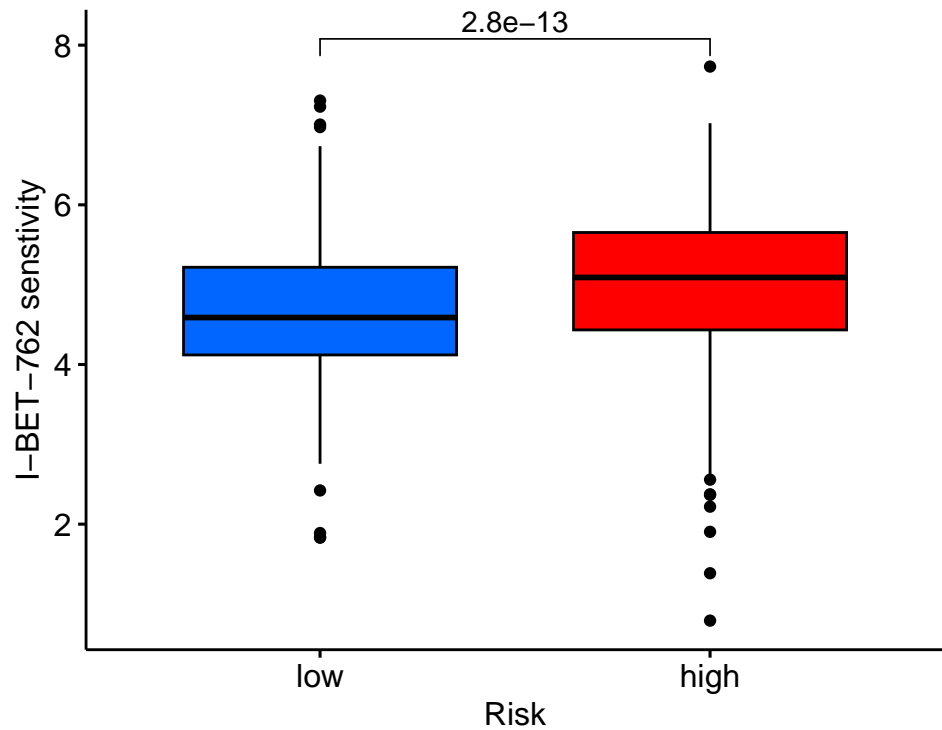

Supplement: Supporting Information 3 — Distinct patterns of drug sensitivity between low-risk and high-risk groups. [file 3423698.f3.zip › Supplementary Material 3/drugSenstivity.I-BET-762.pdf]

Risk 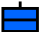 low 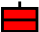 high

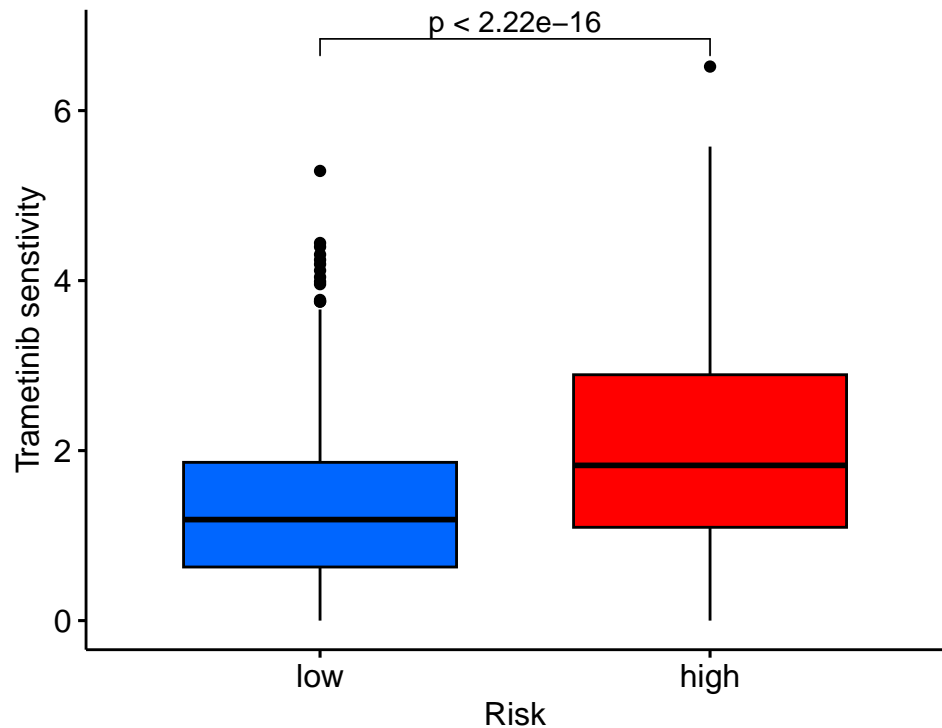

Supplement: Supporting Information 3 — Distinct patterns of drug sensitivity between low-risk and high-risk groups. [file 3423698.f3.zip › Supplementary Material 3/drugSenstivity.Trametinib.pdf]

Risk 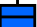 low 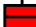 high

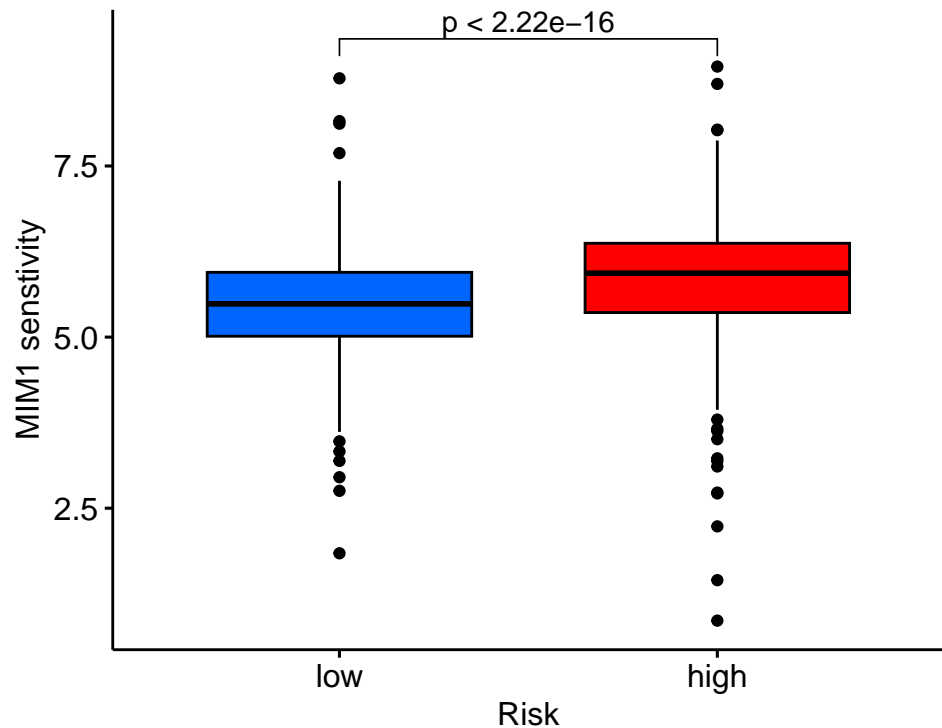

Supplement: Supporting Information 3 — Distinct patterns of drug sensitivity between low-risk and high-risk groups. [file 3423698.f3.zip › Supplementary Material 3/drugSenstivity.MIM1.pdf]

Risk 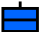 low 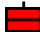 high

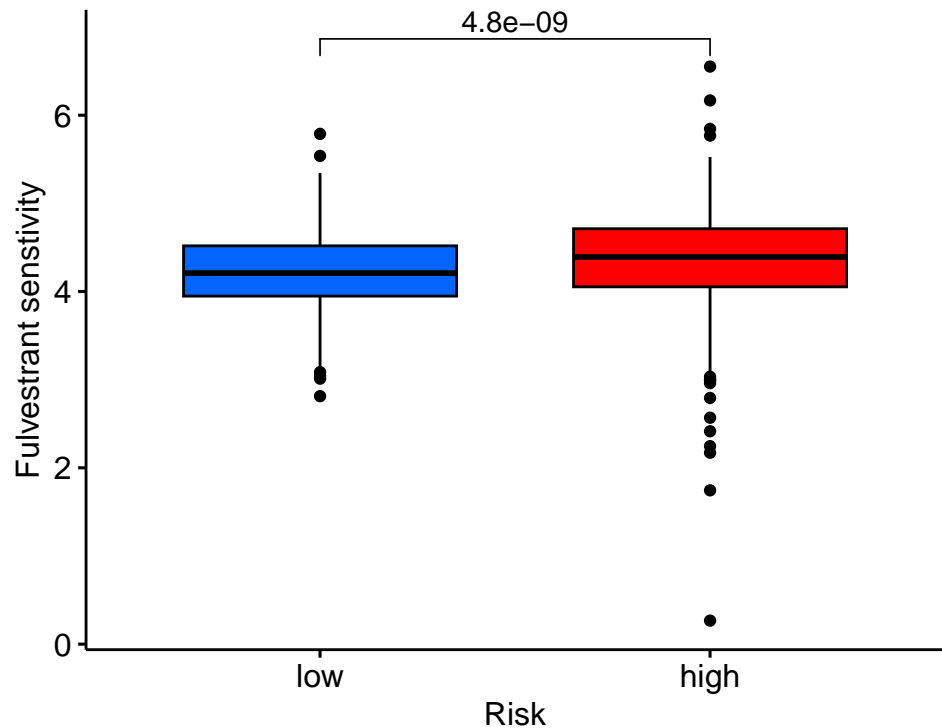

Supplement: Supporting Information 3 — Distinct patterns of drug sensitivity between low-risk and high-risk groups. [file 3423698.f3.zip › Supplementary Material 3/drugSenstivity.Fulvestrant.pdf]

Risk 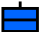 low 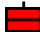 high

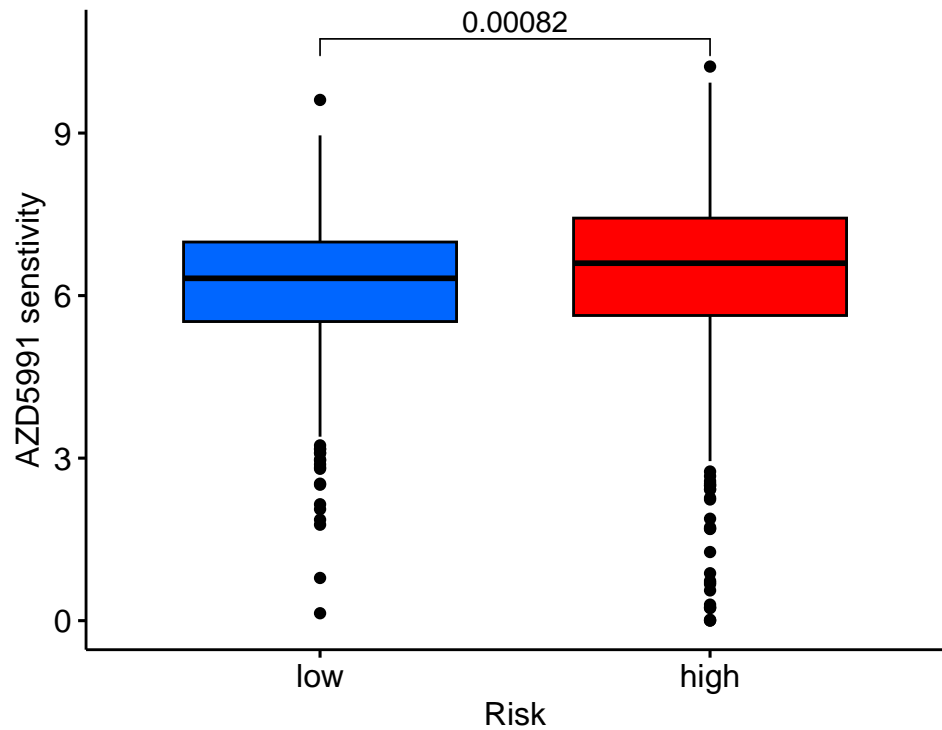

Supplement: Supporting Information 3 — Distinct patterns of drug sensitivity between low-risk and high-risk groups. [file 3423698.f3.zip › Supplementary Material 3/drugSenstivity.AZD5991.pdf]

Risk 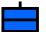 low 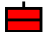 high

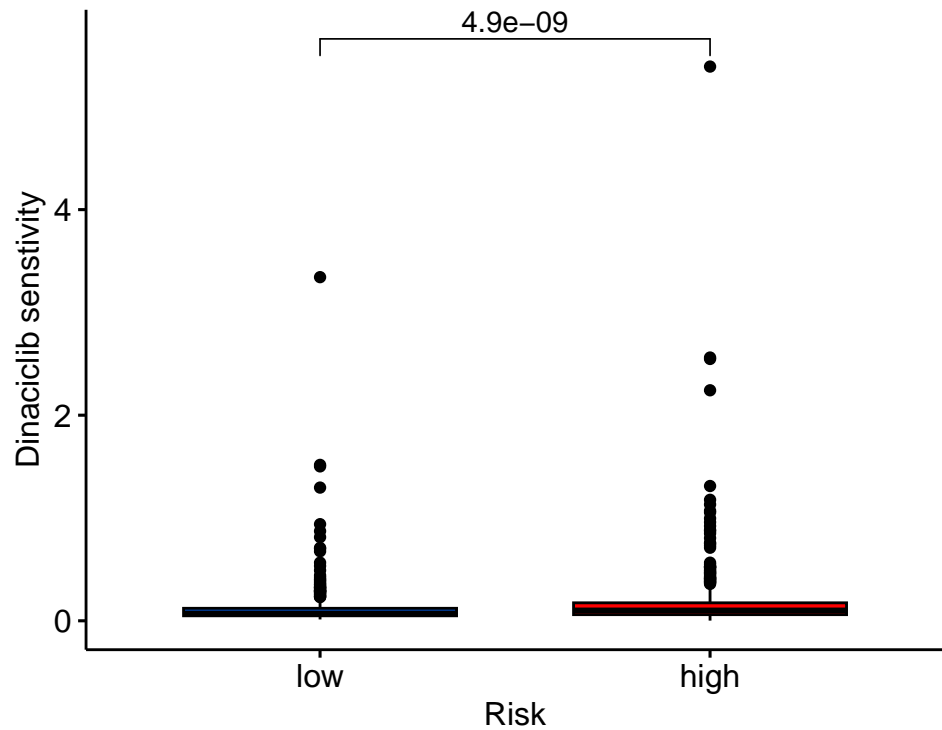

Supplement: Supporting Information 3 — Distinct patterns of drug sensitivity between low-risk and high-risk groups. [file 3423698.f3.zip › Supplementary Material 3/drugSenstivity.Dinaciclib.pdf]

Risk 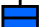 low 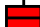 high

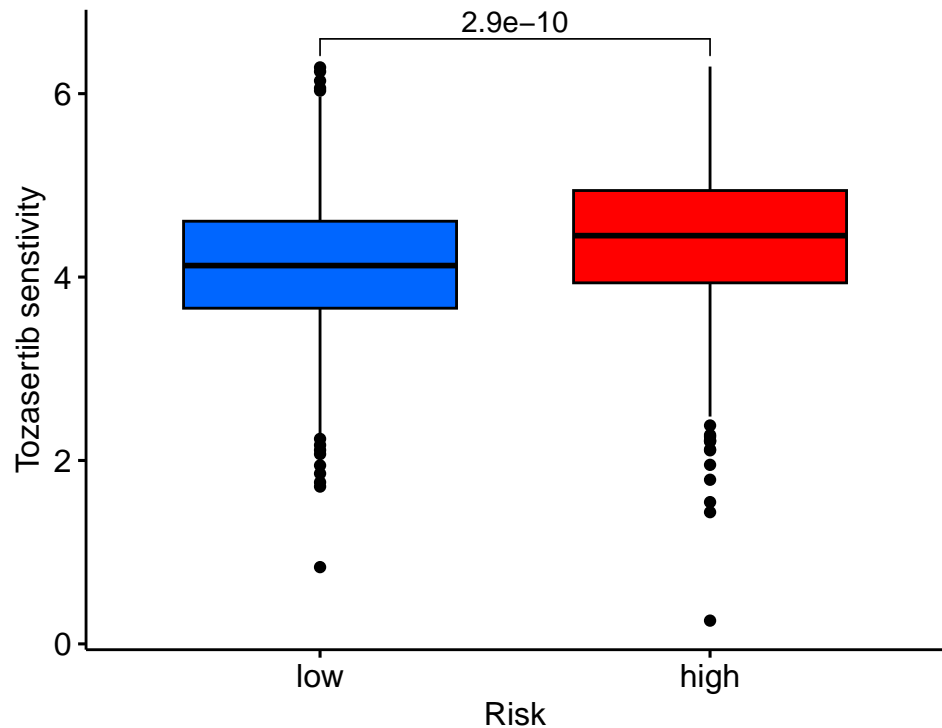

Supplement: Supporting Information 3 — Distinct patterns of drug sensitivity between low-risk and high-risk groups. [file 3423698.f3.zip › Supplementary Material 3/drugSenstivity.Tozasertib.pdf]

Risk low high

$9.7e-14$

WIKI4 sensitivity

12

10

8

6

4

low

high

Risk

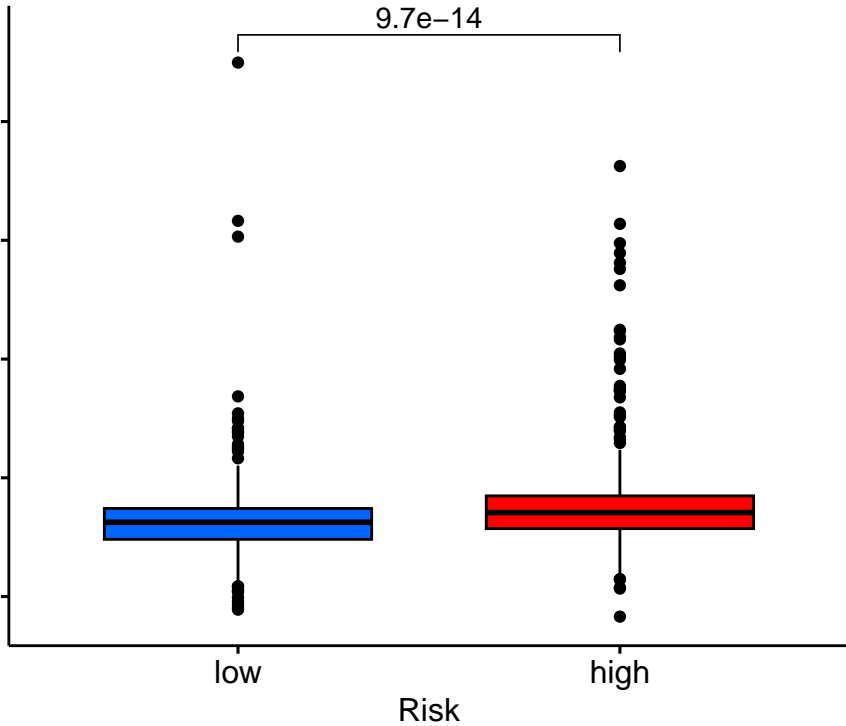

Supplement: Supporting Information 3 — Distinct patterns of drug sensitivity between low-risk and high-risk groups. [file 3423698.f3.zip › Supplementary Material 3/drugSenstivity.WIKI4.pdf]

Risk 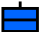 low 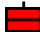 high

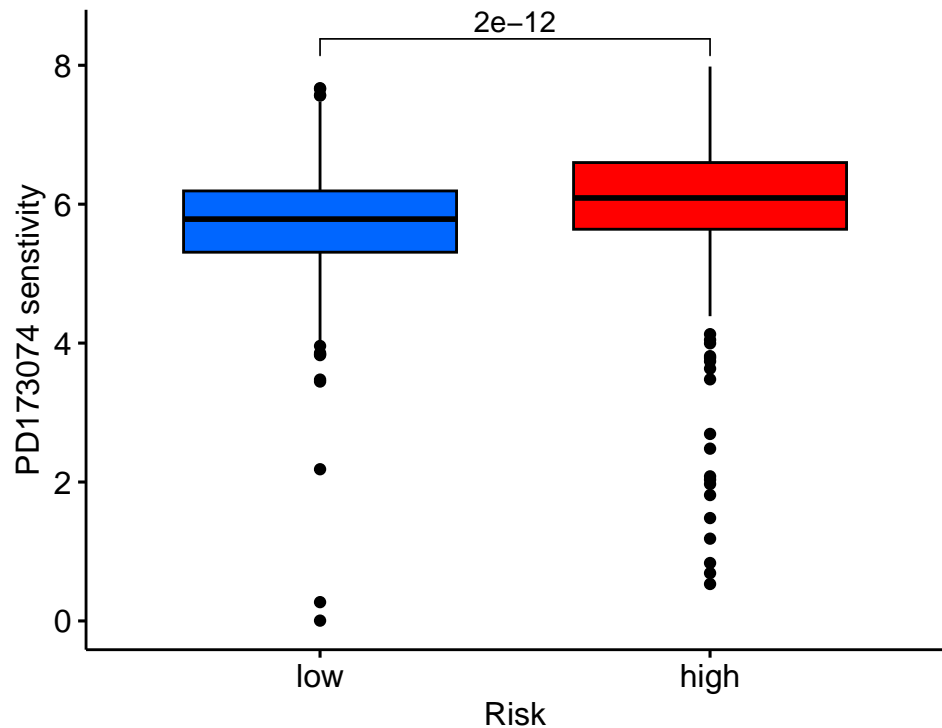

Supplement: Supporting Information 3 — Distinct patterns of drug sensitivity between low-risk and high-risk groups. [file 3423698.f3.zip › Supplementary Material 3/drugSenstivity.PD173074.pdf]

Risk 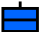 low 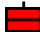 high

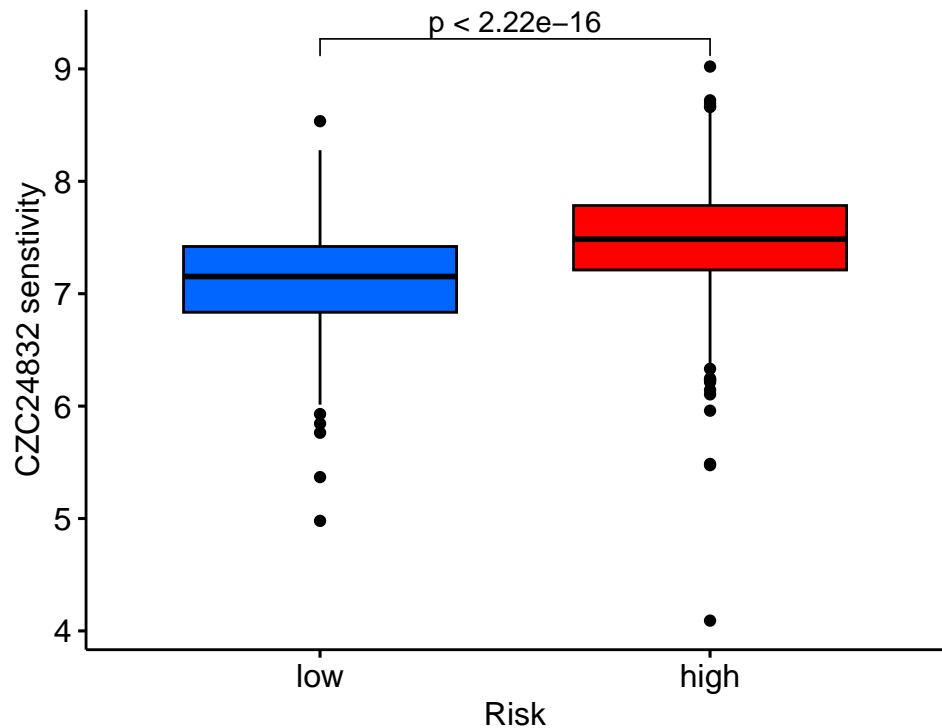

Supplement: Supporting Information 3 — Distinct patterns of drug sensitivity between low-risk and high-risk groups. [file 3423698.f3.zip › Supplementary Material 3/drugSenstivity.CZC24832.pdf]

Risk 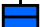 low 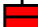 high

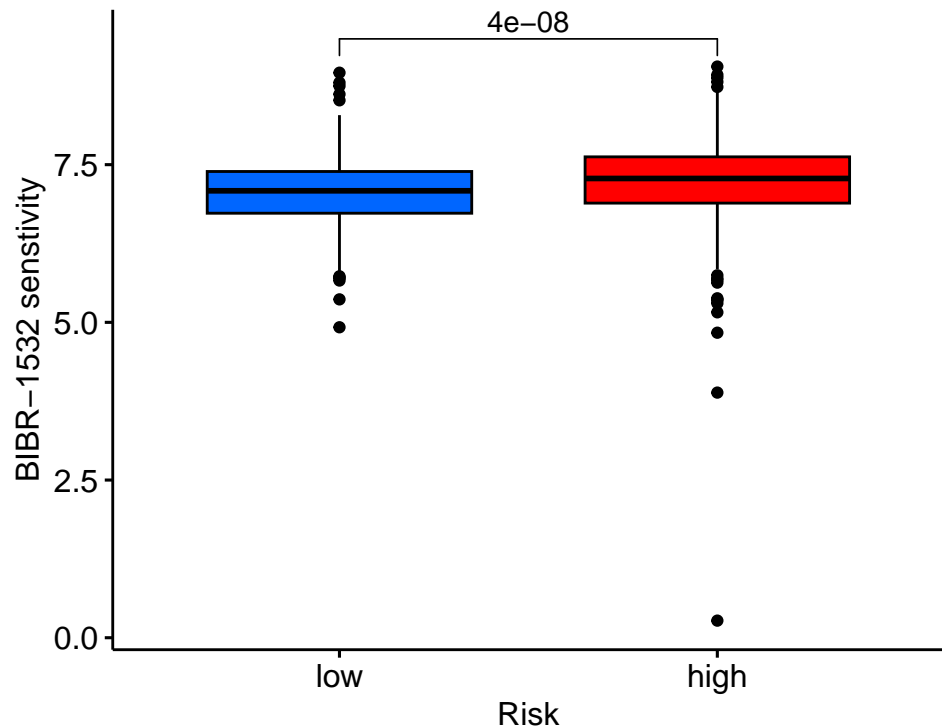

Supplement: Supporting Information 3 — Distinct patterns of drug sensitivity between low-risk and high-risk groups. [file 3423698.f3.zip › Supplementary Material 3/drugSenstivity.BIBR-1532.pdf]

Risk 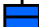 low 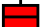 high

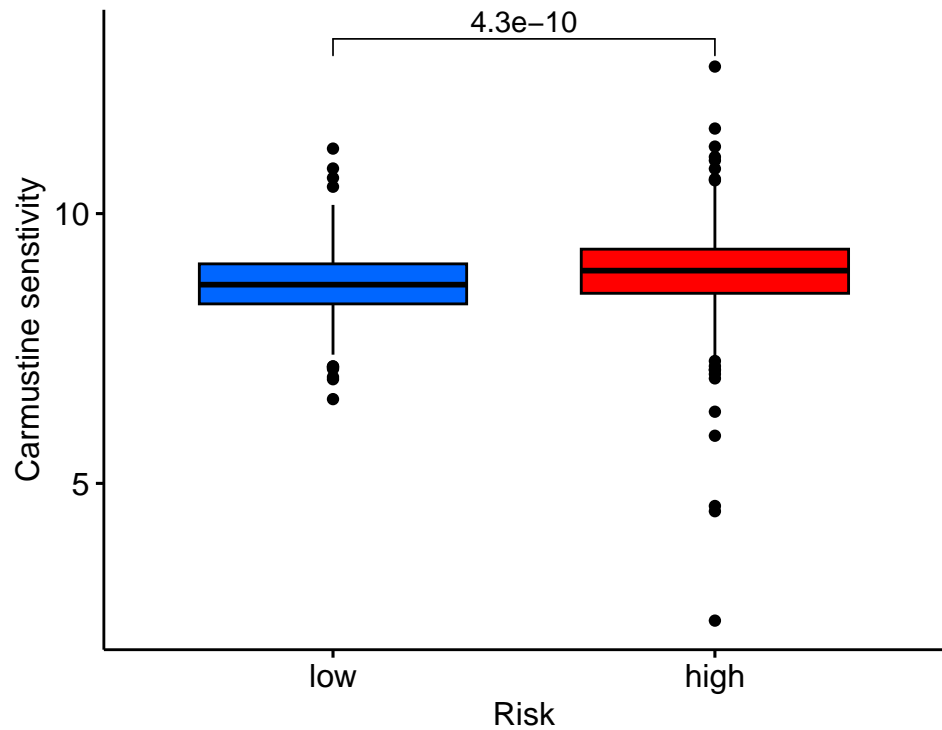

Supplement: Supporting Information 3 — Distinct patterns of drug sensitivity between low-risk and high-risk groups. [file 3423698.f3.zip › Supplementary Material 3/drugSenstivity.Carmustine.pdf]

Risk 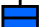 low 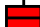 high

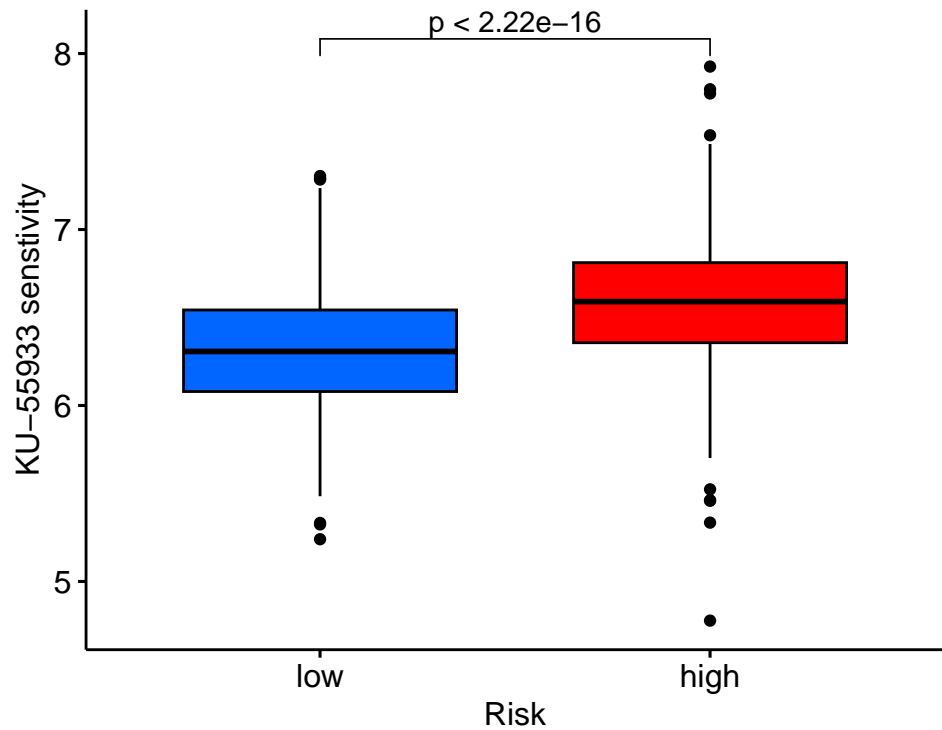

Supplement: Supporting Information 3 — Distinct patterns of drug sensitivity between low-risk and high-risk groups. [file 3423698.f3.zip › Supplementary Material 3/drugSenstivity.KU-55933.pdf]

Risk 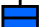 low 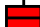 high

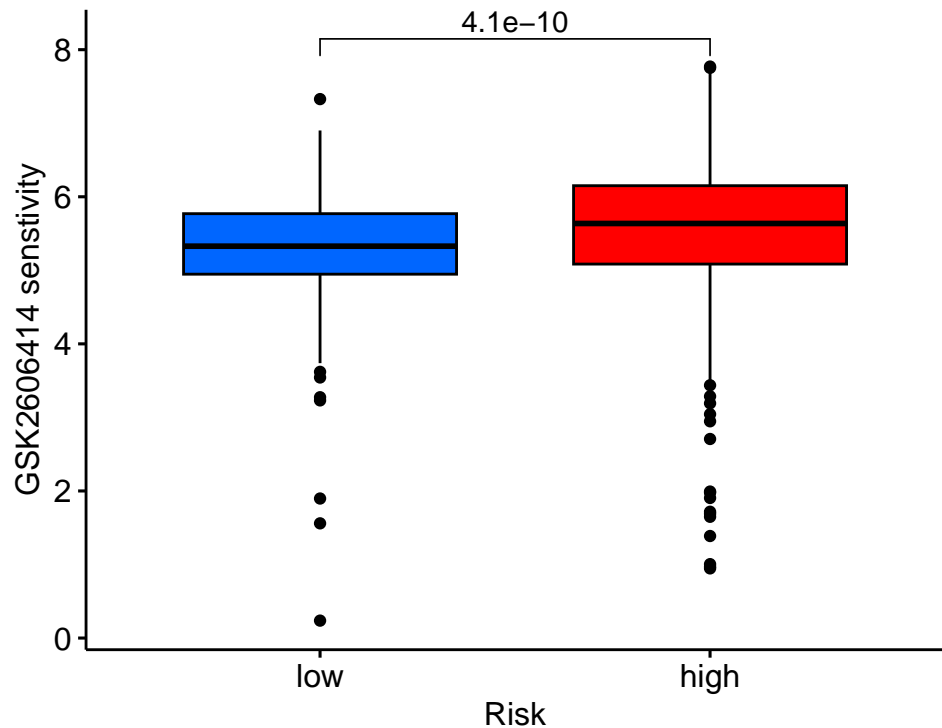

Supplement: Supporting Information 3 — Distinct patterns of drug sensitivity between low-risk and high-risk groups. [file 3423698.f3.zip › Supplementary Material 3/drugSenstivity.GSK2606414.pdf]

Risk 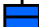 low 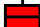 high

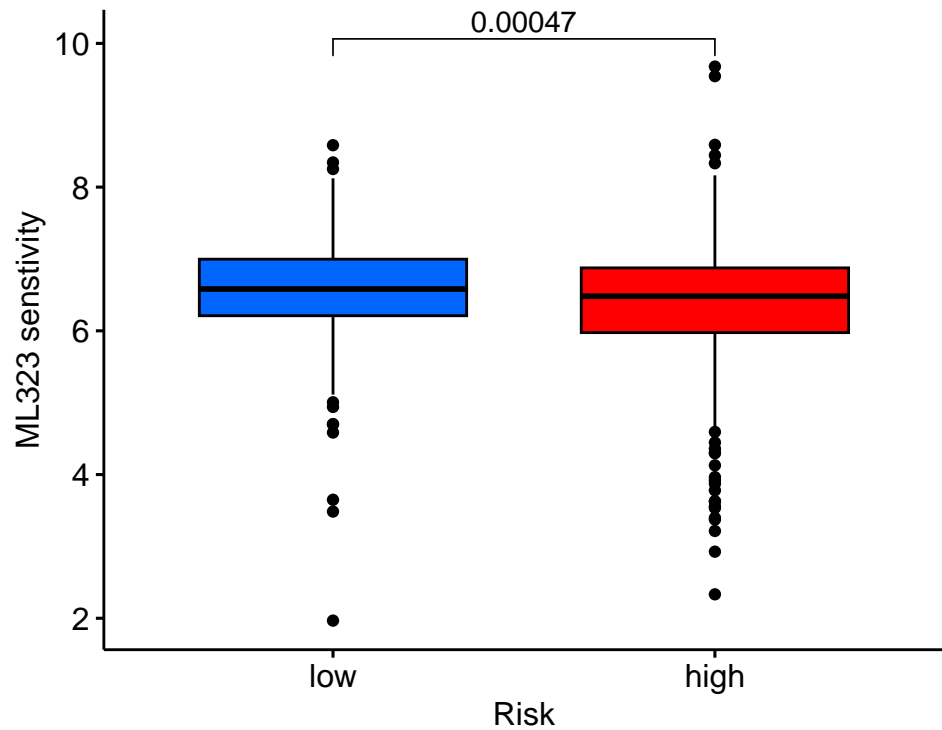

Supplement: Supporting Information 3 — Distinct patterns of drug sensitivity between low-risk and high-risk groups. [file 3423698.f3.zip › Supplementary Material 3/drugSenstivity.ML323.pdf]

Risk low high

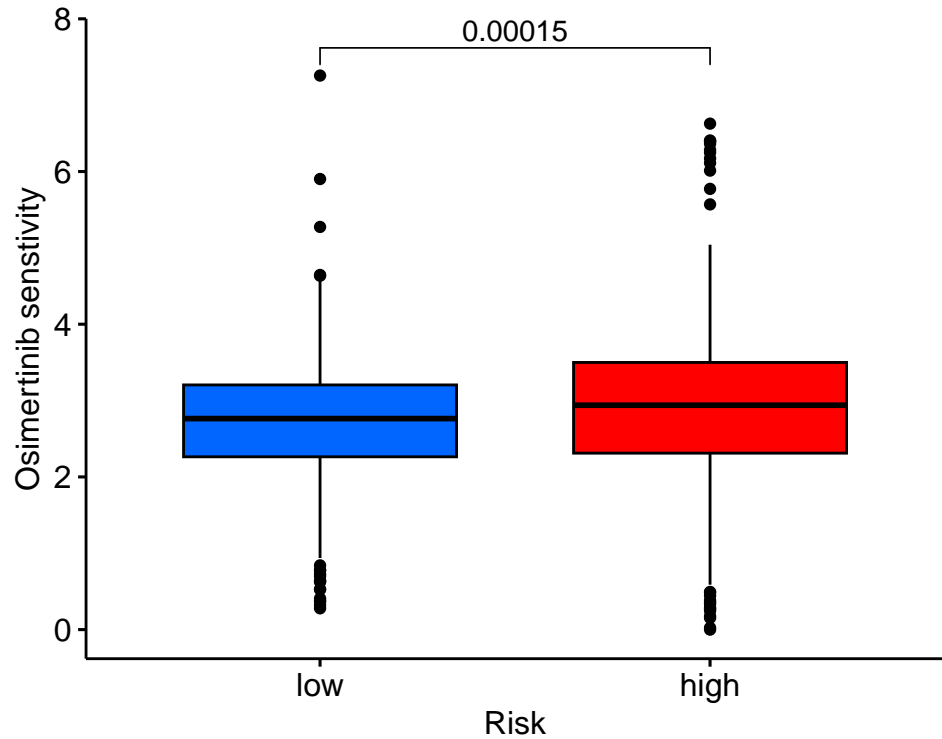

Supplement: Supporting Information 3 — Distinct patterns of drug sensitivity between low-risk and high-risk groups. [file 3423698.f3.zip › Supplementary Material 3/drugSenstivity.Osimertinib.pdf]

Risk 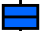 low 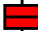 high

5.7e-11

Docetaxel sensitivity

0.6

0.4

0.2

0.0

low

high

Risk

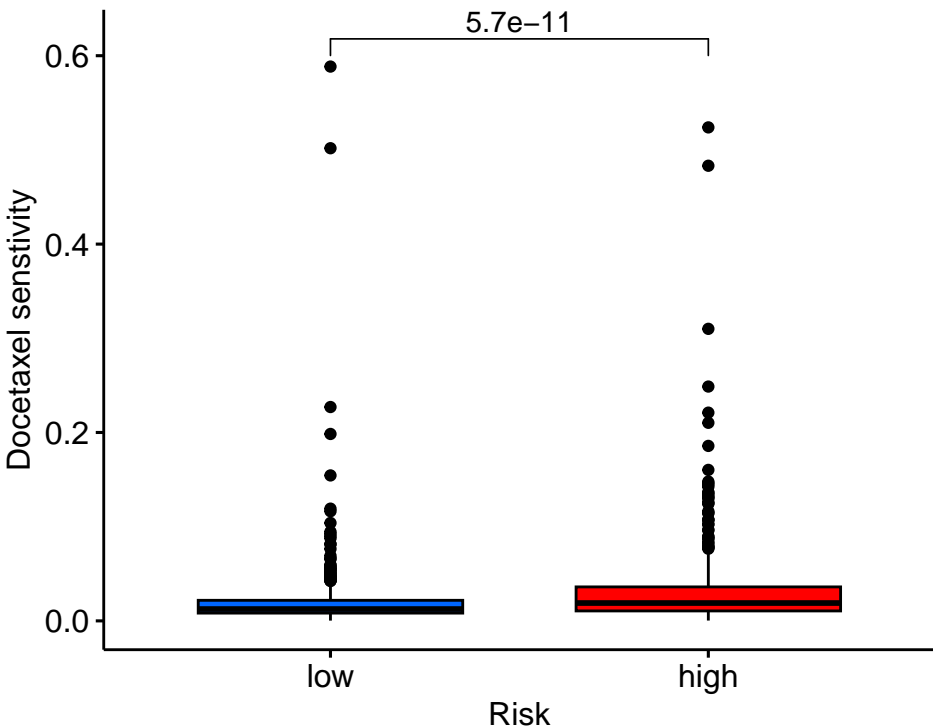

Supplement: Supporting Information 3 — Distinct patterns of drug sensitivity between low-risk and high-risk groups. [file 3423698.f3.zip › Supplementary Material 3/drugSenstivity.Docetaxel.pdf]

Risk low high

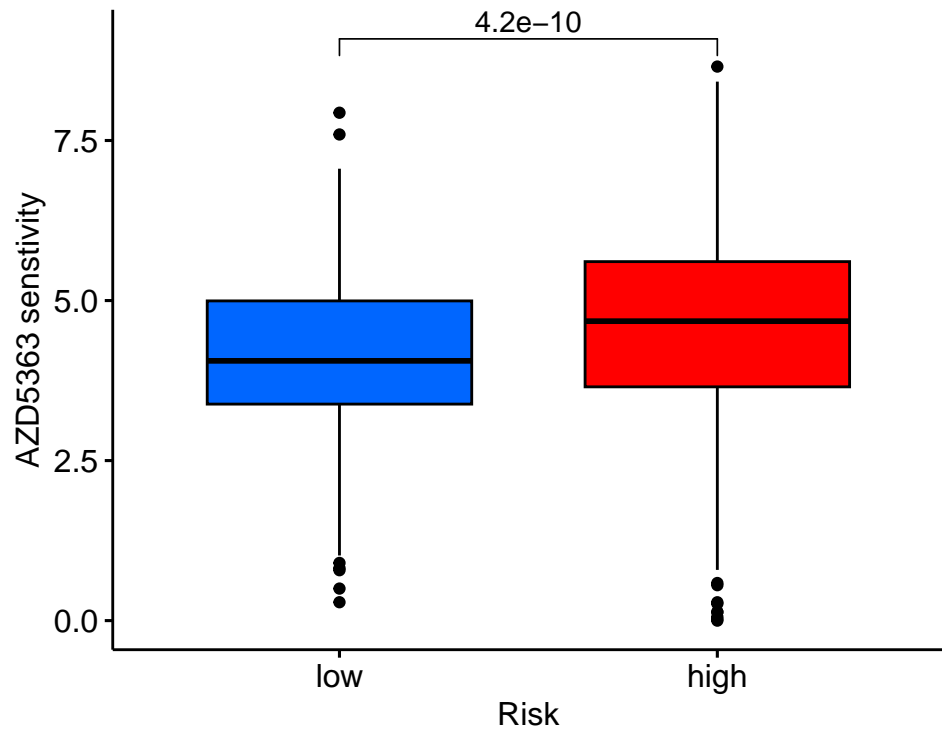

Supplement: Supporting Information 3 — Distinct patterns of drug sensitivity between low-risk and high-risk groups. [file 3423698.f3.zip › Supplementary Material 3/drugSenstivity.AZD5363.pdf]

Risk 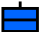 low 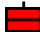 high

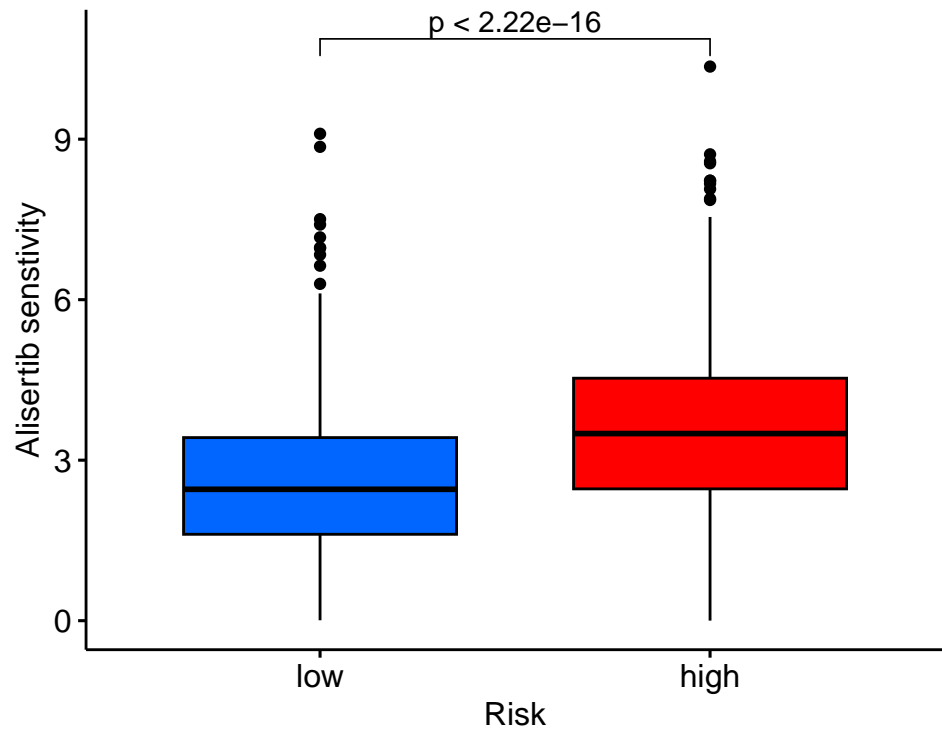

Supplement: Supporting Information 3 — Distinct patterns of drug sensitivity between low-risk and high-risk groups. [file 3423698.f3.zip › Supplementary Material 3/drugSenstivity.Alisertib.pdf]

Risk low high

$p < 2.22\text{e-}16$

AZD7762 sensitivity

low

high

Risk

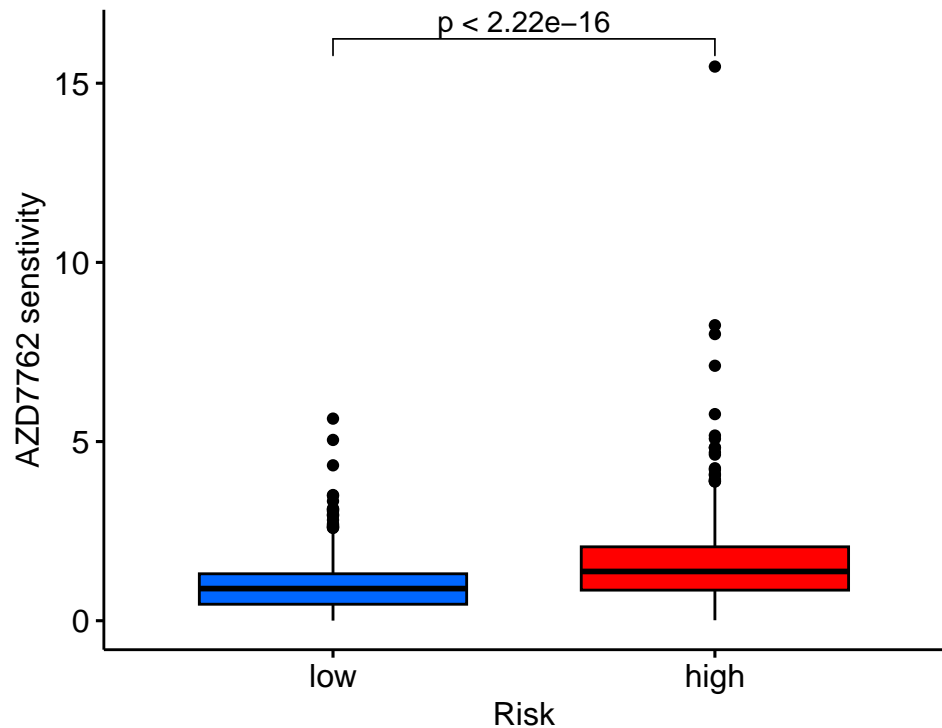

Supplement: Supporting Information 3 — Distinct patterns of drug sensitivity between low-risk and high-risk groups. [file 3423698.f3.zip › Supplementary Material 3/drugSenstivity.AZD7762.pdf]

Risk 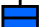 low 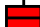 high

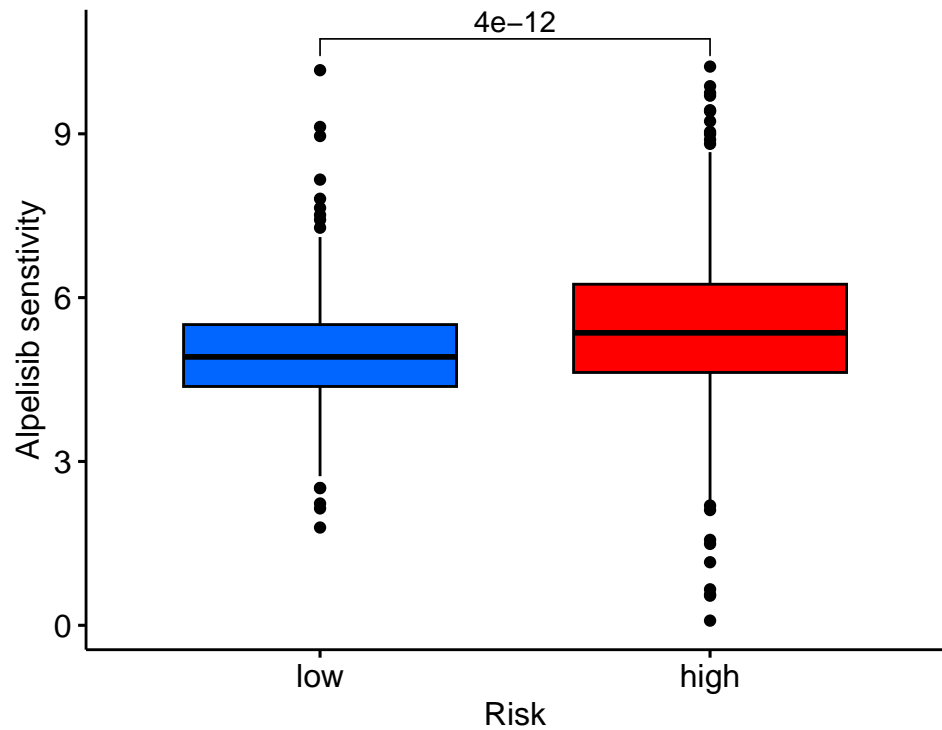

Supplement: Supporting Information 3 — Distinct patterns of drug sensitivity between low-risk and high-risk groups. [file 3423698.f3.zip › Supplementary Material 3/drugSenstivity.Alpelisib.pdf]

Risk low high

1.8e-09

MK-1775 sensitivity

15

10

5

0

low

high

Risk

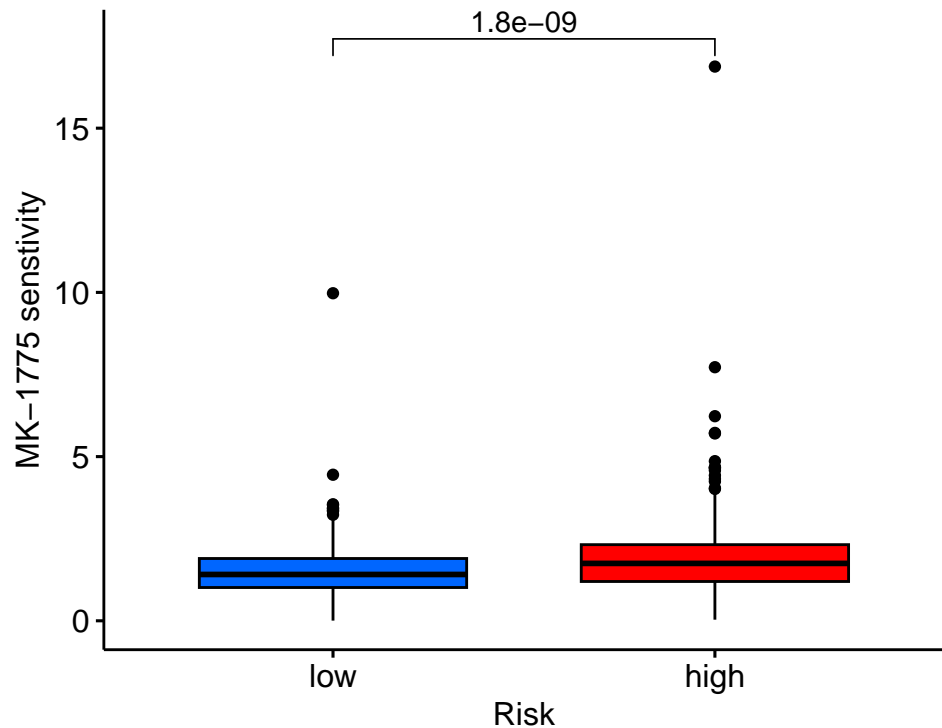

Supplement: Supporting Information 3 — Distinct patterns of drug sensitivity between low-risk and high-risk groups. [file 3423698.f3.zip › Supplementary Material 3/drugSenstivity.MK-1775.pdf]

Risk 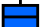 low 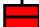 high

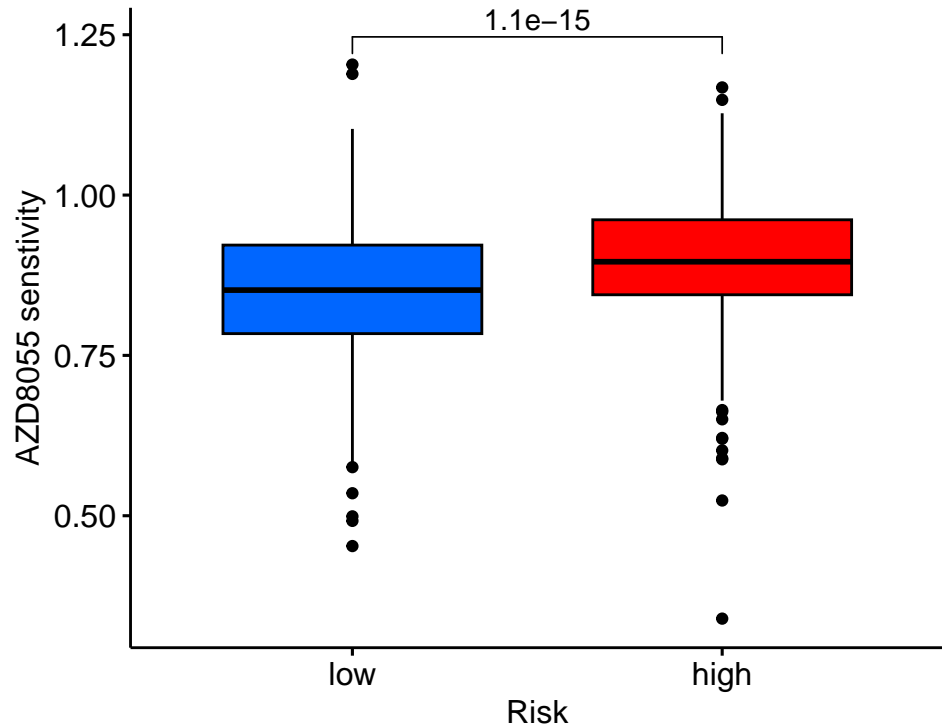

Supplement: Supporting Information 3 — Distinct patterns of drug sensitivity between low-risk and high-risk groups. [file 3423698.f3.zip › Supplementary Material 3/drugSenstivity.AZD8055.pdf]

Risk 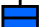 low 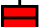 high

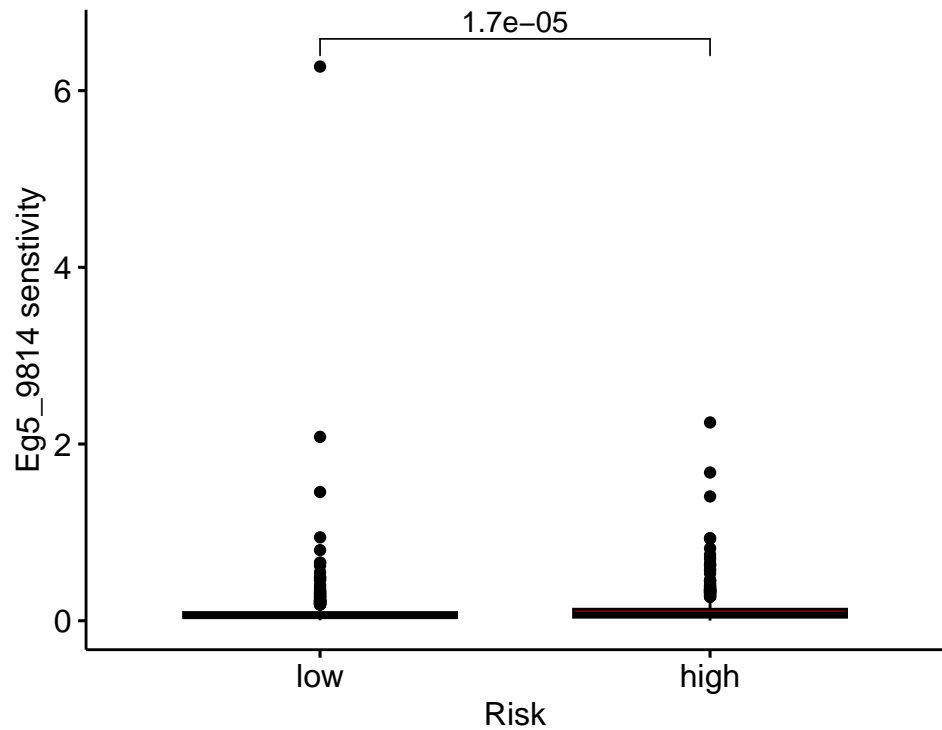

Supplement: Supporting Information 3 — Distinct patterns of drug sensitivity between low-risk and high-risk groups. [file 3423698.f3.zip › Supplementary Material 3/drugSenstivity.Eg5_9814.pdf]

Risk 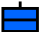 low 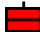 high

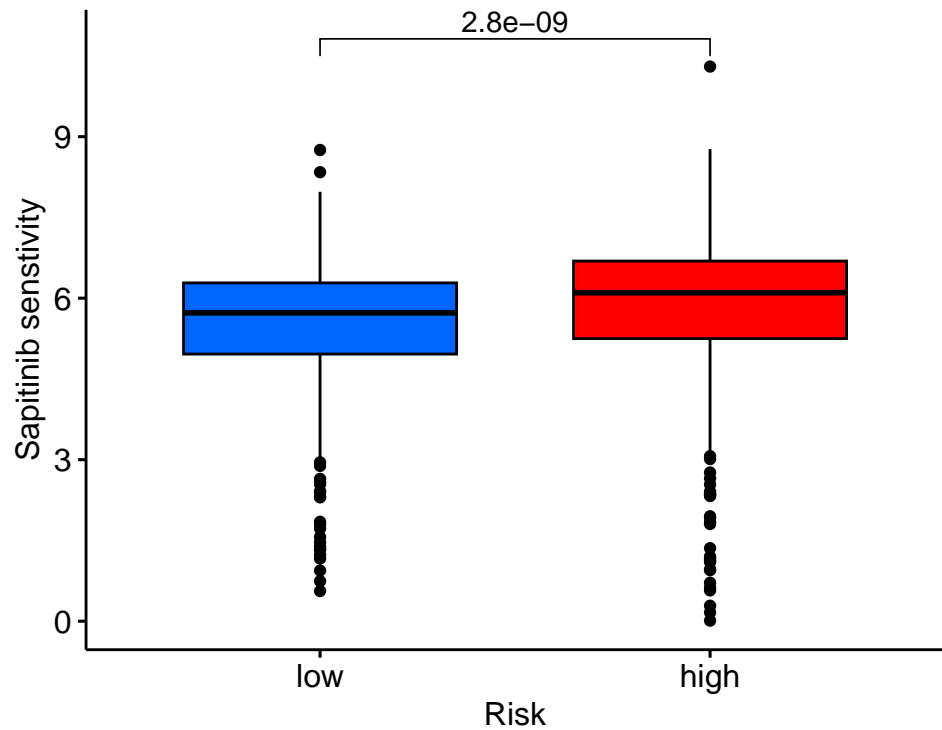

Supplement: Supporting Information 3 — Distinct patterns of drug sensitivity between low-risk and high-risk groups. [file 3423698.f3.zip › Supplementary Material 3/drugSenstivity.Sapitinib.pdf]

Risk 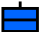 low 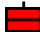 high

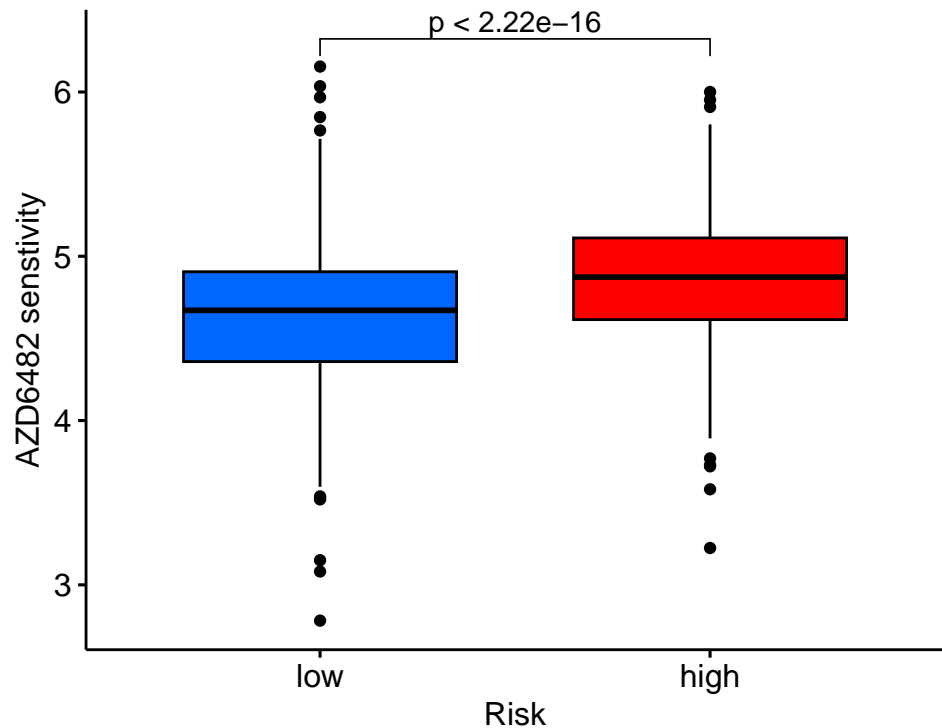

Supplement: Supporting Information 3 — Distinct patterns of drug sensitivity between low-risk and high-risk groups. [file 3423698.f3.zip › Supplementary Material 3/drugSenstivity.AZD6482.pdf]

Risk 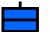 low 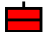 high

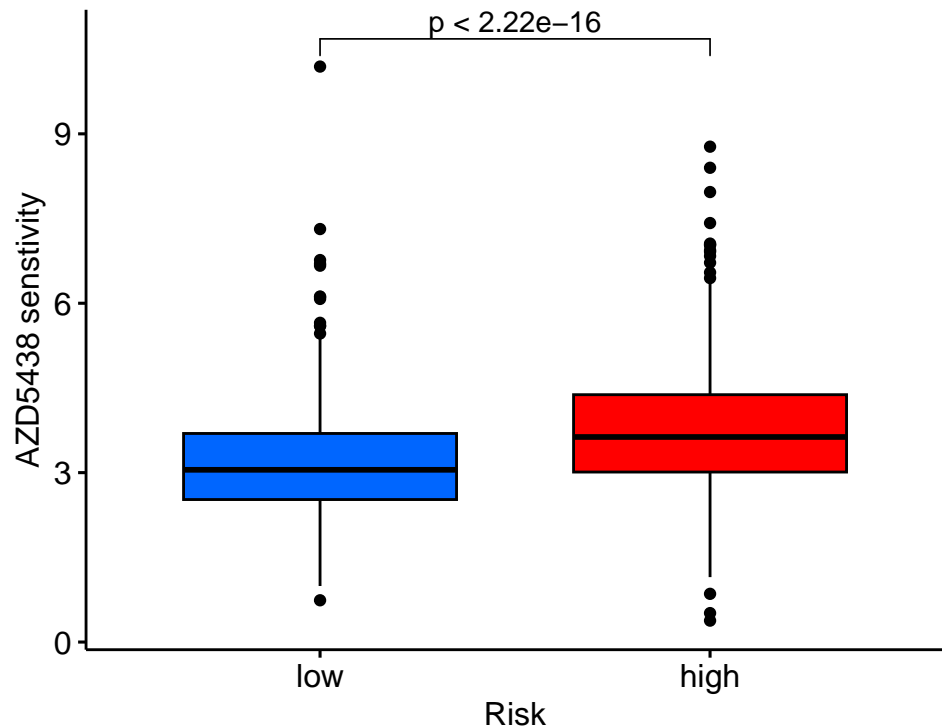

Supplement: Supporting Information 3 — Distinct patterns of drug sensitivity between low-risk and high-risk groups. [file 3423698.f3.zip › Supplementary Material 3/drugSenstivity.AZD5438.pdf]

Risk 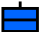 low 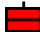 high

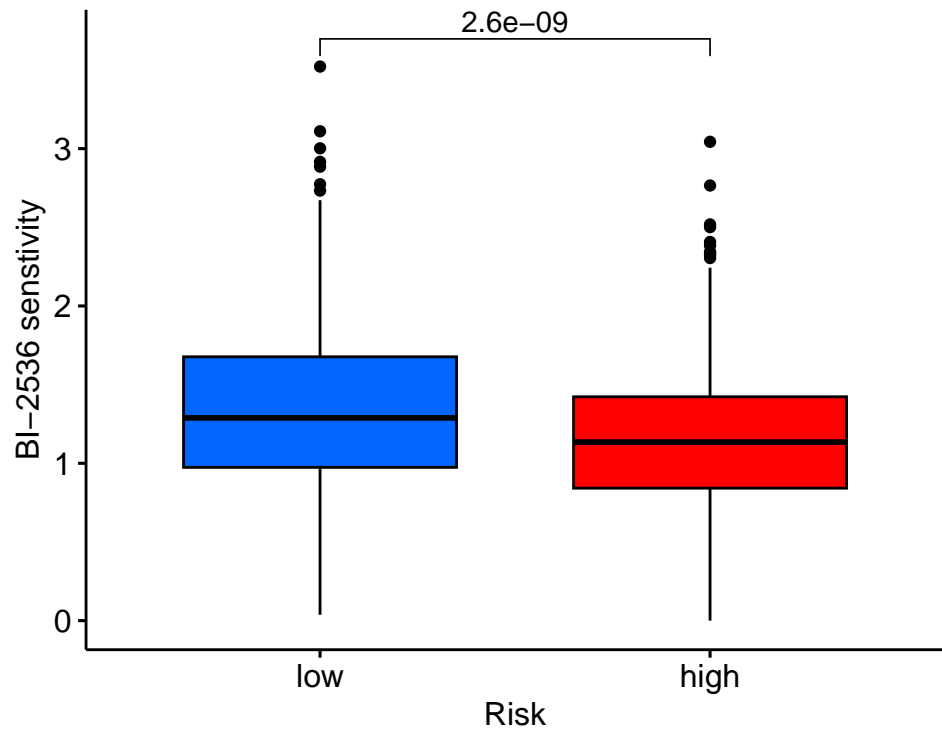

Supplement: Supporting Information 3 — Distinct patterns of drug sensitivity between low-risk and high-risk groups. [file 3423698.f3.zip › Supplementary Material 3/drugSenstivity.BI-2536.pdf]

Risk 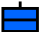 low 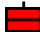 high

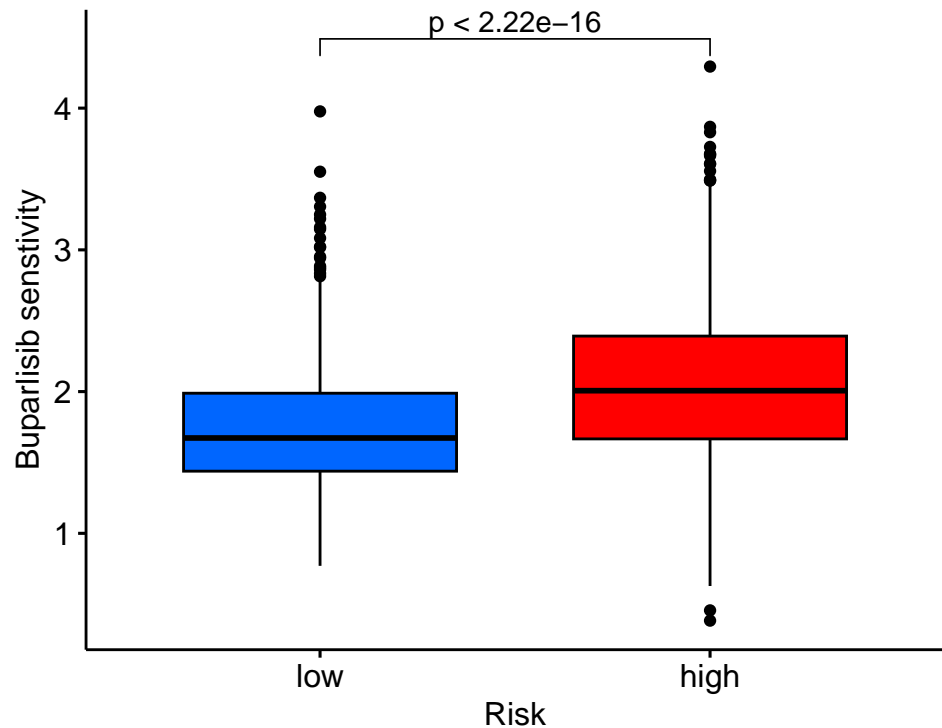

Supplement: Supporting Information 3 — Distinct patterns of drug sensitivity between low-risk and high-risk groups. [file 3423698.f3.zip › Supplementary Material 3/drugSenstivity.Buparlisib.pdf]

Risk 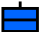 low 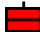 high

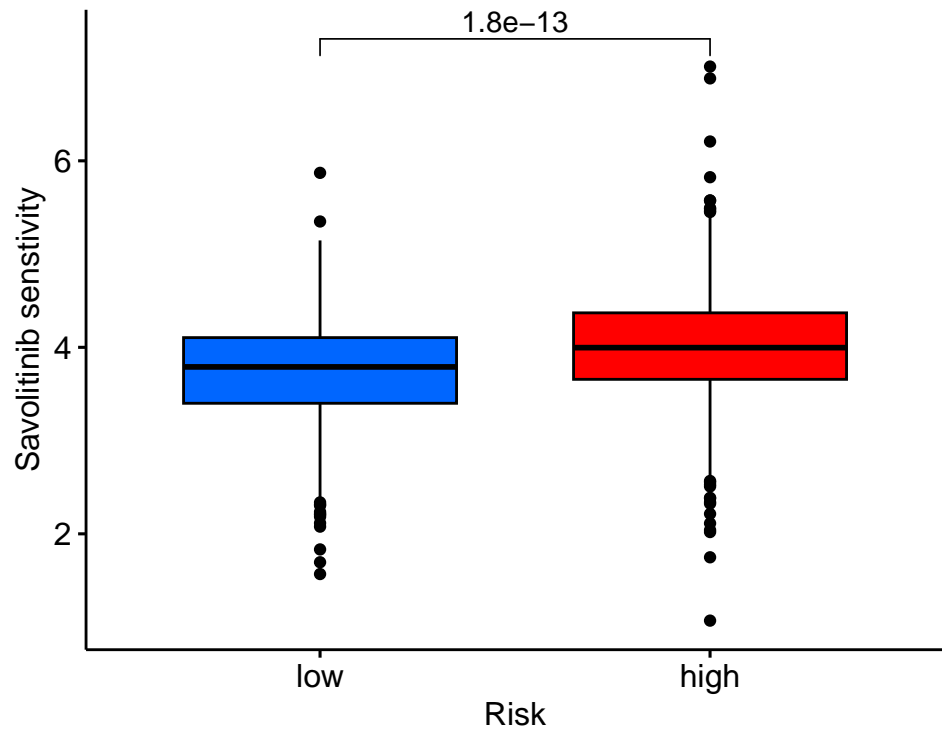

Supplement: Supporting Information 3 — Distinct patterns of drug sensitivity between low-risk and high-risk groups. [file 3423698.f3.zip › Supplementary Material 3/drugSenstivity.Savolitinib.pdf]

Risk 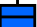 low 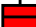 high

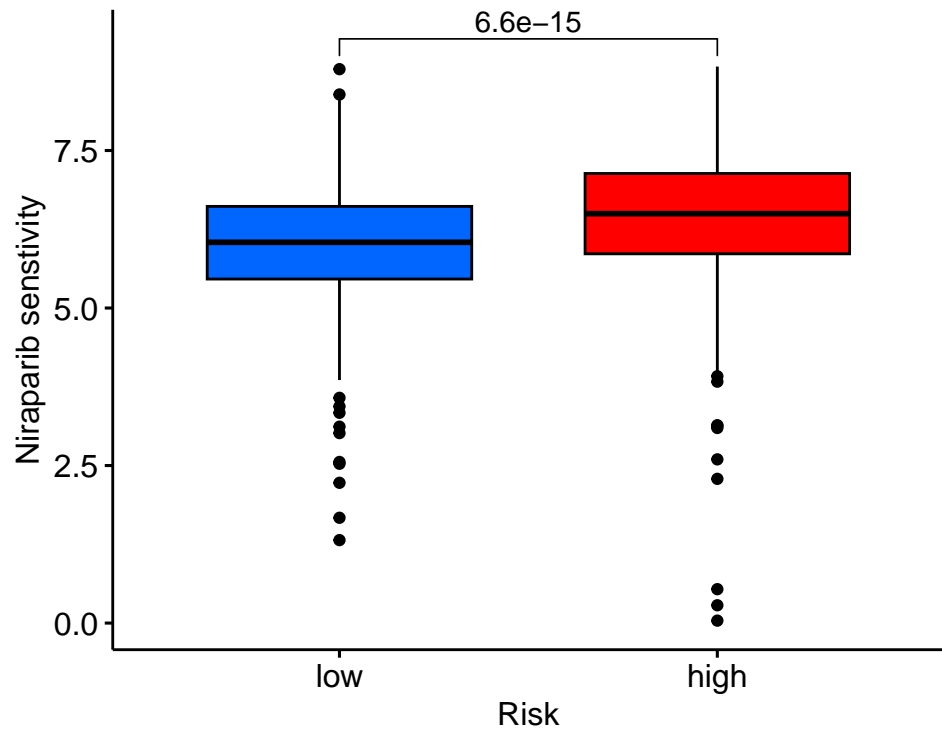

Supplement: Supporting Information 3 — Distinct patterns of drug sensitivity between low-risk and high-risk groups. [file 3423698.f3.zip › Supplementary Material 3/drugSenstivity.Niraparib.pdf]

Risk 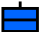 low 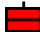 high

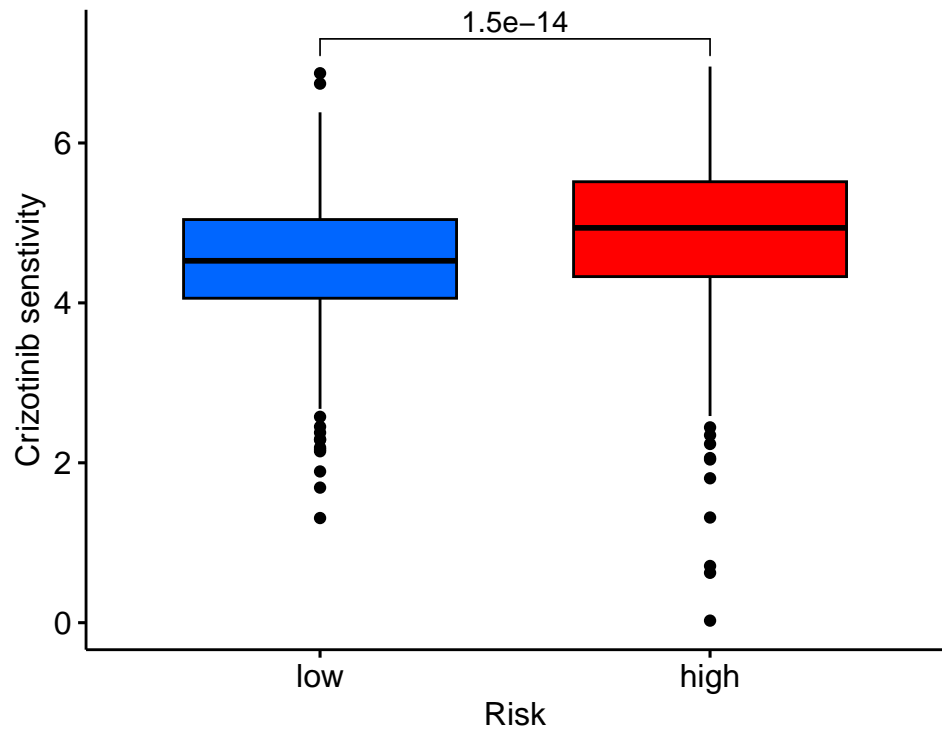

Supplement: Supporting Information 3 — Distinct patterns of drug sensitivity between low-risk and high-risk groups. [file 3423698.f3.zip › Supplementary Material 3/drugSenstivity.Crizotinib.pdf]
